# Nonenhanced Photon Counting CT of the Head: Impact of the keV Level, Iterative Reconstruction and Calvaria on Image Quality in Monoenergetic Images

**DOI:** 10.1007/s00062-023-01331-w

**Published:** 2023-08-17

**Authors:** Arwed Elias Michael, Denise Schoenbeck, Matthias Michael Woeltjen, Jan Boriesosdick, Jan Robert Kroeger, Christoph Moenninghoff, Sebastian Horstmeier, Julius Henning Niehoff, Christoph Kabbasch, Lukas Goertz, Jan Borggrefe

**Affiliations:** 1 Department of Radiology, Neuroradiology and Nuclear Medicine, Johannes Wesling University Hospital, Ruhr University Bochum, Bochum, Germany; 2 Department of Radiology and Neuroradiology, University Hospital of Cologne, Cologne, Germany; 3 Johannes Wesling University Hospital by Muehlenkreiskliniken AöR, Hans-Nolte-Straße 1, 32429 Minden, Germany

**Keywords:** Computed tomography, Photon counting detector, Nonenhanced CT of the head, Virtual monoenergetic images, Iterative reconstruction, Quantum iterative reconstruction

## Abstract

**Purpose:**

Nonenhanced computed tomography (CT) of the head is among the most commonly performed CT examinations. The spectral information acquired by photon counting CT (PCCT) allows generation of virtual monoenergetic images (VMI). At the same time, image noise can be reduced using quantum iterative reconstruction (QIR). In this study, the image quality of VMI was evaluated depending on the keV level and the QIR level. Furthermore, the influence of the cranial calvaria was investigated to determine the optimal reconstruction for clinical application.

**Methods:**

A total of 51 PCCT (NAEOTOM Alpha, Siemens Healthineers, Erlangen, Germany) of the head were retrospectively analyzed. In a quantitative analysis, gray and white matter ROIs were evaluated in different brain areas at all available keV levels and QIR levels with respect to signal, noise, signal-to-noise ratio (SNR), and contrast-to-noise ratio (CNR). The distance to the cranial calvaria of the ROIs was included in the analysis. This was followed by a qualitative reading by five radiologists including experienced neuroradiologists.

**Results:**

In most ROIs, signal and noise varied significantly between keV levels (*p* < 0.0001). The CNR had a focal maximum at 66 keV and an absolute maximum at higher keV, slightly differently located depending on ROI and QIR level. With increasing QIR level, a significant reduction in noise was achieved (*p* < 0.0001) except just beneath the cranial calvaria. The cranial calvaria had a strong effect on the signal (*p* < 0.0001) but not on gray and white matter noise. In the qualitative reading, the 60 keV VMI was rated best.

**Conclusion:**

In nonenhanced PCCT of the head the selected keV level of the VMI and the QIR level have a crucial influence on image quality in VMI. The 60 keV and 66 keV VMI with high QIR level provided optimal subjective and objective image quality for clinical use. The cranial calvaria has a significant influence on the visualization of the adjacent brain matter; currently, this substantially limits the use of low keV VMIs (< 60 keV).

**Supplementary Information:**

The online version of this article (10.1007/s00062-023-01331-w) contains supplementary material, which is available to authorized users.

## Introduction

Nonenhanced computed tomography of the head (NCTH) is one of the most performed CT examinations. Many intracranial pathologies can be diagnosed or ruled out NCTH. Most frequently, NCTH is a standard imaging method for the detection of stroke, intracranial hemorrhages and other neurologic emergencies [1].

In 2021, a milestone in the history of CT was achieved with the introduction of photon counting computed tomography (PCCT) into clinical diagnostics [2]. The technical features and the resulting advantages for imaging have already been explained in several articles (e.g., [3, 4]). Similar to the well-established dual-energy CT (DECT), PCCTs acquires spectral information and therefore, enables the reconstruction of virtual monoenergetic images (VMI), for which the superiority compared to classical polyenergetic reconstruction has already been suggested for many applications in DECT (e.g., [5–9]).

NCTH with the novel PCCT approved for clinical diagnosis has been investigated in a single study that explored which spectrum of VMI is most useful for clinical diagnostics in general [10]. The existing study was limited to quantitative data which reported single artifact indices. Qualitative data were missing. Since this initial study, considerable improvements were provided by the manufacturer, both regarding scanner hardware and postprocessing software. The iterative reconstruction of the PCCT, called quantum iterative reconstruction (QIR), was refined for the application in PCCT. Moreover, the influence of QIR on image quality in CCT has not been investigated yet.

In the present study a comprehensive subjective and objective analysis of the image quality in current PCCT was performed. In addition to the influence of the kilo-electron volt (keV) levels of VMI, the influence of QIR was investigated. Furthermore, it was hypothesized that the thickness of the skull calvaria may have an influence on image quality. As this has not yet been explored, we conducted a quantitative analysis for a detailed assessment. The overall aim of the study was to find the optimal reconstruction for use in clinical practice.

## Material and Methods

### Patient Population

Institutional review board approval was obtained. Informed consent was waived due to the retrospective study design. All patients who had undergone photon counting NCTH at our institutions between July and October 2022 were identified. To exclude confounders, patients with acute or chronic intracranial disease diagnosed on CCT were excluded; these criteria included intracranial hemorrhages, infarcts, masses, extensive leukoencephalopathy, edema of any type, and implants/foreign objects. Similarly, examinations with motion artifacts were excluded. The data of the included patients were anonymized.

### CT Protocol and Image Acquisition

All CT scans were performed using the clinically approved photon-counting CT (NAEOTOM Alpha, software version Syngo CT VA50, Siemens Healthineers, Erlangen, Germany) with a spiral CT protocol. All patients were examined in supine position with moderate flexion in the cervical spine to perform axial acquisition in the orbitomeatal plane. After the topogram was made, a lens shield was applied. Single collimation was 0.4 mm, total collimation 38.4 mm, and pitch factor was 0.55 with a rotation time of 0.5 s. Tube voltage was 120 kVp, and tube current was modulated due to the manufacturer’s program of dose modulation. The matrix size was 512 × 512, and the field of view (FOV) was optimized for the individual head size. The reconstruction kernel QR36 was used for the spectral data sets. The primary data were further processed without and with all possible levels of quantum iterative reconstruction (QIR, levels 0–4). The manufacturer-specific spectral workstation (Syngo.Via, VB60_B version, Siemens Healthineers) was used to analyze the datasets. The images were reconstructed in axial view with a slice thickness of 3 mm and a slice increment of 3 mm.

### Quantitative Image Analysis

Overall, 16 different ROIs were used for the quantitative data analysis; these included ROIs from similar preceding studies [11, 12]. 9 ROIs involved the gray and white matter of the neocortex below the calvaria at a distance of 5–20 mm. 6 ROIs involved the basal ganglia, the thalamus and the immediately adjacent white matter in each case. Finally, the last ROI was placed in the pons between the petrous bones to provide a measure of artifacts analogous to the preliminary studies [10] (for a detailed description of the ROIs including accompanying figures, please refer to the Supplemental Material).

**Fig. 1:**
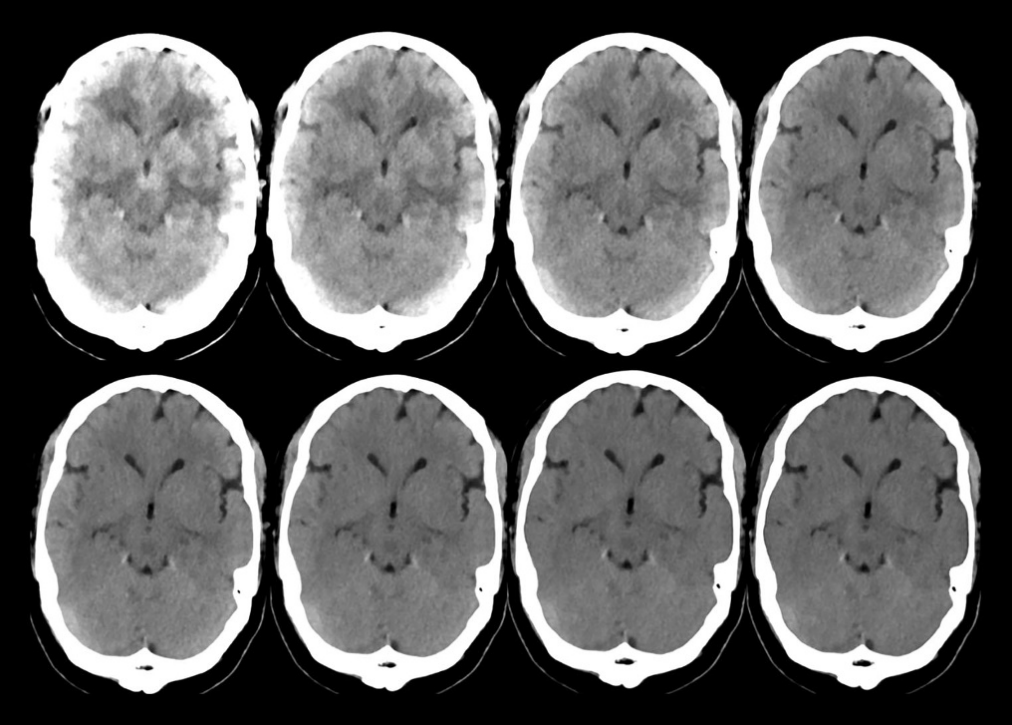
An exemplary selection of virtual monoenergetic images of an axial reconstruction of a NCTH for an overview. From top left to bottom right: 40 keV, 50 keV, 60 keV, 70 keV, 80 keV, 90 keV, 120 keV, 150 keV. The window settings are in all cases Center 40/Width 70. *keV* kilo electron Volt

Within the spectral datasets of all levels of the QIR (levels 0–4), each ROI was set identically. A total of 151 virtual monoenergetic reconstructions (VMI) with kilo electron Volt (keV) levels from 40 keV to 190 keV in 1keV steps were prepared (for an exemplary selection see Fig. 1). For each ROI, both the average density and its standard deviation in Hounsfield Units were measured in every QIR level and every VMI.

Parameters for assessing image quality were determined as described previously, in order to allow for comparison of study results [10, 13]. The signal of the ROIs was defined as the mean density/attenuation in Hounsfield units (HU). Noise was defined as the standard deviation (SD) of ROIs in HU. The SD of the ROI in the pons between the petrous bones was referred to as the posterior fossa artifact index (PFAI) [12]. The signal-to-noise ratio (SNR) was calculated by dividing the mean density of the ROI (signal) by the corresponding SD of the ROI (noise). The contrast-to-noise (CNR) ratio was calculated as the quotient of the difference of the mean density of two adjacent ROIs with gray matter (GM) and white matter (WM) and the square root of the sum of the variance of both ROIs [10]:

$$CNR=\frac{\mathrm{mean}_{GM}-\mathrm{mean}_{WM}}{\sqrt{{SD}_{GM}^{2}+{SD}_{WM}^{2}}}$$

### Qualitative Image Analysis

The image quality of specific VMIs (keV: 40, 50, 60, 66, 70, 75, 90, 100, selected based on the quantitative results to include the VMI with the maximum signal and CNR) in QIR level 4 was evaluated by 5 radiologists: 2 experienced neuroradiologists with 17 and 12 years of experience, 2 experienced general radiologists with 8 and 6 years of experience and 1 resident with 2 years of experience. The quality of gray-white matter differentiation, the extent of noise and the general clinical usability were rated using 5‑point Likert scales (from 1 = difficult, uncertain diagnosis to 5 = excellent, fully diagnostic). The five readers were blinded to the keV level and other algorithms used for postprocessing. Every VMI was presented with equal standard windowing using a commonly accepted setting (Center 40/Width 80), as there is currently no evidence for the optimal window settings of different VMI in NCTH.

### Additional Measurements

After evaluation of the quantitative measurements the influence of the cranial calvaria was investigated for its causality. For general assessment of calvarial artifacts we identified two patients with craniectomy shortly before reimplantation in the available period. In the NCTH of these 2 patients, 10 ROIs each with a size of 4 mm were placed on the side with cranial calvaria with a distance of approximately 5–10 mm and on the side of craniectomy 10 ROIs were placed at 5–10 mm from the dura. In both hemispheres only brain parenchyma was included that was unharmed.

### Statistical Analysis

Data processing and statistical analyses were performed using the statistical software R (Version 4.1.0, [14]) and RStudio (Version 2022.07.1 + 554, Posit PBC, Boston, USA). The Shapiro–Wilk test was applied to test for normal distribution, the Levene test to check for homoscedasticity. When considering the individual ROIs, a one-way ANOVA or the Friedman test as the corresponding nonparametric method was used to investigate whether the image parameters of the individual keV levels differed. Using the Bonferroni method, the resulting *p*-values were corrected. For post hoc testing with selected keV levels, e.g., the keV level with the maximum or minimum value of a parameter and the adjacent keV levels as well as a representative selection of the spectrum, the paired T‑test or the nonparametric Wilcoxon signed rank test were used. Again, the correction of the *p*-values was done using the Bonferroni method.

For qualitative analysis, appropriate nonparametric procedures were used (Friedman test and Wilcoxon test). If not stated otherwise, all data are presented as mean ± standard deviation.

## Results

### Patient Population and Radiation Dose

After identification of all NCTH in the abovementioned time period and exclusion of the examinations following the described criteria, 51 patients (25 men, 26 women) were included in this study. The mean age was 70.5 ± 17.4 years (range 22–93 years).

The average effective tube current was 271.2 ± 21 mA, the mean CTDIvol was 48.8 ± 3.7 mGy and the mean dose length product was 803.7 ± 76.1 mGy*cm.

### Quantitative Analysis: keV Level

#### Signal

In all cortical ROIs and the adjacent white matter, a high signal was found in the low keV levels, which then decreased monotonically with increasing keV. The differences between the keV levels were significant (Friedman test: corrected *p* < 0.0001). The QIR did not significantly influence the tissue density. In the gray matter 20 mm below the calotte, the maximum signal was 51.12 ± 6.20 HU in the 40 keV VMI in the reconstruction with fourth iterative level (Q4). In post hoc testing, the difference to the signal in the 41 keV VMI (50.31 ± 5.63 HU) was significant (corrected *p* < 0.0001). The signal of the white matter showed similar results but was generally lower than the signal of the gray matter. With increasing keV level, this difference became smaller. In the 40 keV VMI at QIR level 4, the white matter signal 20 mm below the calvaria was 42.23 ± 6.91 HU and again in post hoc testing the difference to the 41 keV VMI was significant (corrected *p* < 0.0001). The alteration of the signal of the gray matter of the deep nuclear areas was analogous to that of the cortical gray matter 20 mm beneath the calvaria (exemplified in the caudate head 51.33 ± 4.54 HU in 40 keV Q4). There was no significant difference between the signal values in the different keV levels in the white matter adjacent to the inferior caudate head and the thalamus (Friedman test: corrected *p* > 0.05).

#### Noise

In the cortical ROIs, noise decreased with increasing keV level, and there was a significant difference between the individual VMIs (Friedman test: corrected *p* < 0.0001). With QIR the noise decreased significantly (see later) but with increasing keV level this decrease became smaller. As an example, in the gray matter 20 mm below the calvaria, the lowest noise in QIR level 0 was found at 190 keV (1.25 ± 3.07 HU). In post hoc testing there was a significant difference to lower keV levels starting at 185 keV (corrected *p* = 0.02). In QIR level 4, the lowest noise was found in the 189 keV VMI (1.04 ± 0.93 HU). In the ROIs of the deep nuclear regions and the adjacent white matter, the changes of the noise were similar among the keV levels. In the ROI between the petrous bones in the pons, also known as posterior fossa artifact index (PFAI), the noise dropped significantly (Friedman test: corrected *p* < 0.0001) with increasing keV. The higher the QIR level, the more the minimum of the noise was moved towards lower keV levels (in QIR level 0 at 189 keV, in QIR level 1 at 169 keV, in QIR level 2 at 157 keV, in QIR level 3 at 144 keV and in QIR level 4 at 129 keV), e.g., in QIR level 4, the maximum PFAI was measured at 40 keV VMI (10.13 ± 1.97 HU), while the lowest PFAI was 1.47 ± 2.51 HU at 129 keV. It is noticeable that in all ROIs there were focal accentuation of noise reduction found between 60 keV and 70 keV, which leaves a small dip in the otherwise exponentially decreasing curve.

#### SNR

The signal to noise ratio (SNR) was lowest at low keV levels and continuously increased with higher keV levels (Friedman test: corrected *p* < 0.0001). Depending on the extent of QIR, the maximum SNR was found in different regions of the higher keV levels. In Q4, the maximum SNR was for most ROIs in the range of 120–150 keV, e.g., in the gray matter of the posterior thalamus it was measured at 142 keV in Q4 with 93.55 ± 30.09. In post hoc testing, the difference to the lower keV level was significant up to 140 keV (corrected *p* = 0.01), and to the higher keV from 143 keV on (corrected *p* < 0.0001). The focal decrease in noise between 60 and 70 keV was reflected in the focal increase of the SNR in this range.

#### CNR

**Fig. 2:**
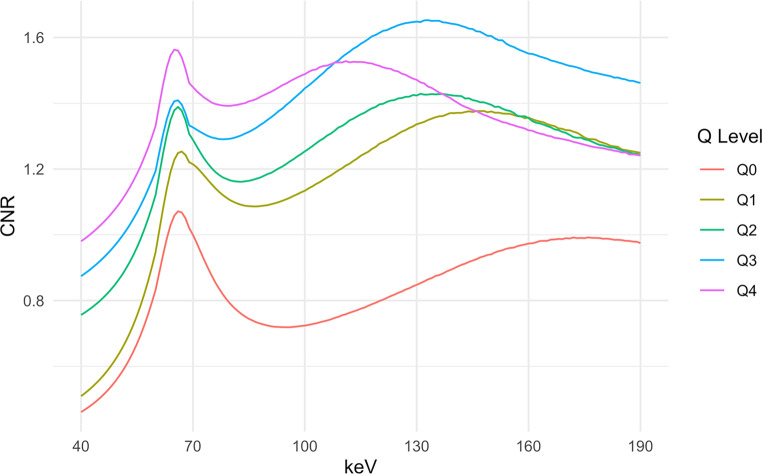
Plot of the average CNR of the ROIs in gray and white matter 15 mm below the calvaria (ROI 6 and ROI 7) as a function of keV level at different levels of QIR. *CNR* contrast-to-noise-ratio, *Q* *Level* quantum iterative reconstruction level. At this distance to the calvaria, two peaks of CNR are found. The first peak is between 60 and 70 keV, the second peak is in the higher range, the exact position depending on the level of QIR

Regarding the CNR, there were significant differences between the different keV levels in all QIR levels (Friedman test: corrected *p* < 0.0001). Directly below the calvaria, the CNR was highest at 1.29 ± 0.98 in the 40 keV Q3. At 5 mm and 10 mm below the calvaria, the CNR was highest between 60 and 70 keV; 5 mm below the calvaria in the 65 keV Q3 reconstructions (0.91 ± 0.6), 10 mm below the calvaria in the 64 keV Q4 reconstructions (1.30 ± 0.71). With increasing distance from the cranial calvaria, the maximum of the CNR shifted to a higher range of keV leading to a two-peaked curve (see Fig. 2). The first peak was between 60 and 70 keV; the focal reduction of noise in this range resulted in a focal increase of the CNR. After this focal maximum, the CNR then continued to increase until it reached an absolute maximum. Exemplarily, this absolute maximum at the caudate head in Q3 was 4.74 ± 0.59 at 141 keV, and the first maximum was at 66 keV in Q4 (2.42 ± 0.71, please also refer to the supplemental material for a more detailed presentation of the results of the analysis of signal, noise, SNR and CNR including more figures).

### Quantitative Analysis: QIR

Based on the results of the quantitative analysis of the VMI quality parameters as a function of keV level, 4 keV levels were chosen for the analysis of noise as a function of QIR: 40 keV, 66 keV, 100 keV, and 190 keV. These keV levels contain the VMI with the maximum signal, CNR and minimum noise and an intermediate level. As stated above, the noise decreased with increasing keV. For all ROIs except the ROI immediately below the cranial calvaria, there was a significant reduction in noise (Friedman test: corrected *p* < 0.0001, in post hoc testing corrected *p* < 0.0001) at low keV levels with each level of QIR. For example, in the white matter 10 mm below the cranial calvaria (ROI 5), the noise in the 40 keV VMI was 13.56 HU (95% confidence interval [CI] 12.86–15.26 HU) in Q0, 9.74 HU (95% CI 9.13–10.35 HU) in Q1, 8.28 HU (95% CI 7.78–8.78 HU) in Q2, 7.16 HU (95% CI 6.72–7.60 HU) in Q3, and finally 5.63 HU (95% CI 5.30–5.96 HU) in Q4. In the 66 keV and 100 keV the noise itself and also the reduction of the noise was much smaller but still significant. In the 190 keV VMI there was no significant difference of the noise between the different levels of the QIR (Friedman test: corrected *p* > 0.05). In some ROIs even increasing noise with increasing level of QIR was noticeable (e.g., in the white matter 15 mm below the cranial calvaria, ROI 7: 190 keV Q0 0.54 ± 0.24 to Q4 0.55 ± 0.26, see also Table 1).

**Table 1:** Noise as a function of quantum iterative reconstruction (QIR) at 40 keV, 66 keV, 100 keV and 190 keV

ROI	keV	Q0	Q1	Q2	Q3	Q4
GM cortical 10 mm	40	12.79 [11.96; 13.63]	9.67 [9.06; 10.27]	8.24 [7.74; 8.75]	6.93 [6.44; 7.43]	5.48 [5.09; 5.88]
	66	4.96 [4.61; 5.3]	3.97 [3.6; 4.34]	3.51 [3.17; 3.84]	3.01 [2.75; 3.27]	2.66 [2.4; 2.92]
	100	2.39 [2.06; 2.73]	1.99 [1.59; 2.4]	1.84 [1.48; 2.2]	1.6 [1.3; 1.9]	1.46 [1.16; 1.76]
	190	1.63 [1.23; 2.03]	1.44 [1; 1.87]	1.47 [1.07; 1.86]	1.33 [1; 1.66]	1.29 [0.96; 1.61]
GM caudate head	40	15.65 [15.11; 16.18]	12.08 [11.4; 12.77]	10.34 [9.81; 10.87]	8.06 [7.56; 8.56]	6.5 [6.19; 6.81]
	66	5.27 [4.95; 5.6]	4.08 [3.84; 4.32]	3.46 [3.22; 3.71]	2.78 [2.59; 2.98]	2.23 [2.1; 2.37]
	100	2.06 [1.95; 2.18]	1.51 [1.39; 1.63]	1.35 [1.23; 1.47]	1.12 [1.01; 1.23]	0.96 [0.83; 1.08]
	190	0.78 [0.65; 0.9]	0.66 [0.53; 0.79]	0.7 [0.56; 0.84]	0.72 [0.58; 0.86]	0.71 [0.58; 0.83]

Data presented as mean [95 % confidence interval]. All levels of QIR are listed. *Q0* no iterative reconstruction, *Q1* first level of QIR, *Q2–Q4* second to fourth level of QIR. As an example, a cortical ROI (GM cortical 10 mm: gray matter 10 mm below the calvaria, ROI 4) and a ROI in the deep nuclear regions (GM caudate head: gray matter in the superior caudate head, ROI 10) are given.

*keV* kilo electron Volt, *QIR* quantum iterative reconstruction, *GM* gray matter

An exception regarding the general influence of the QIR is the ROI directly below the cranial calvaria. In this particular ROI there is no significant reduction of noise with increasing QIR at any of the keV levels, likewise no trend is visible.

### Quantitative Analysis: Cranial Calvaria

**Fig. 3:**
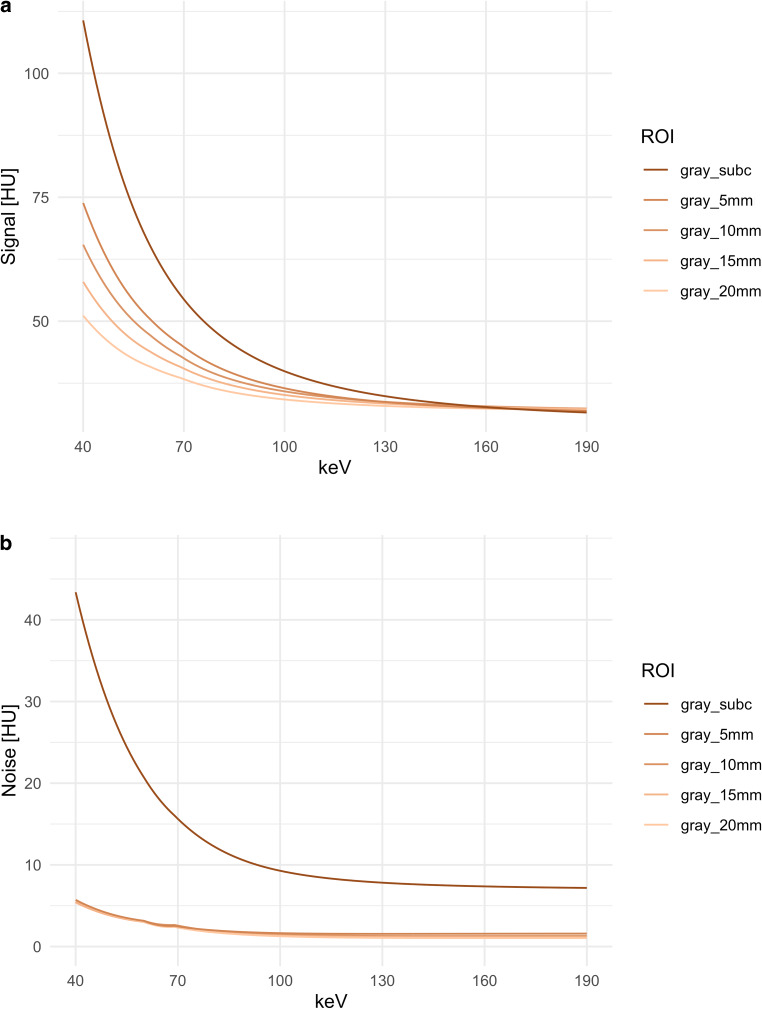
Signal (**a**) and noise (**b**) as a function of distance from the cranial calvaria. *gray_subc* gray matter directly beneath the calvaria, *gray_5–20* *mm* gray matter with 5, 10, 15, and 20 mm distance to the calvaria. The signal shows a monotonous decrease with increasing distance to the calvaria, 20 mm below the calvaria the signal course then resembles that of the deep gray matter and the adjacent white matter, respectively. The noise, on the other hand, is very highly pronounced immediately below the cranial calvaria; from 5 mm below the calvaria, it is much lower and does not drop further with increasing distance

As a function of distance from the cranial calvaria, there were significant differences in the behavior of signal and noise of the different ROIs across the different keV levels (Friedman test: corrected *p* < 0.0001). The QIR has no influence here. For the signal in the low and medium keV levels the proximity to the calvaria leads to a significant increase of the signal (see Fig. 3a). At high keV there are no significant differences in this respect and at the end of the keV spectrum (> 150 keV) there is even a tendency towards a lower signal. In a linear model there is a very weak (adjusted r^2^ = 0.078) but significant (*p* = 0.0267) relationship between the calvarial thickness and the difference in signal between the ROI in the gray matter 5 mm below the calvaria and the reference ROI in the superior caudate head at 40 keV Q4. In the measurements of patients with hemicraniectomy, gray matter signal below the calvaria averaged 78.34 ± 19.78 HU, on the side without calvaria only 56.28 ± 4.67 HU. The difference between the variances was significant (Levene test *p* < 0.0001). The difference between the means was also significant (Welch test *p* < 0.0001).

The noise, on the other hand, showed a completely different pattern (see Fig. 3). Immediately below the calvaria, noise was significantly increased (Friedman test: corrected *p* < 0.0001) but starting with a distance of 5 mm and more away from the calvaria, there was no further relationship between noise and distance to the calvaria. Thus, we were able to quantify the width of the calvarial artifacts which amounted to 5 mm. In the linear model of calvaria thickness and noise about 5 mm below the calvaria, there was no significant correlation. The data of the patients with hemicraniectomy showed no significant difference of attenuation between the hemispheres (*p* = 0.07); however, there was only a tendency to higher values in the presence of the cortical bone.

### Qualitative Analysis: Reading

**Fig. 4:**
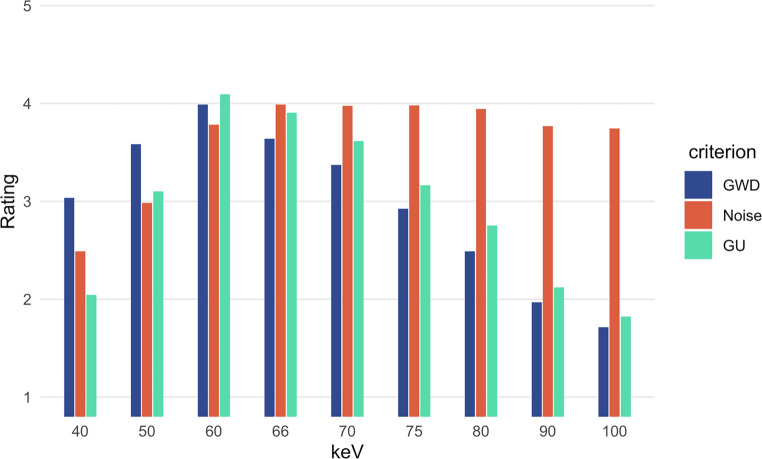
Results of the qualitative analysis. Ratings of gray-white matter differentiation (GWD), noise, and general clinical usability (GU) in VMI with a choice of kilo electron Volt (keV) levels (keV: 40, 50, 60, 66, 70, 75, 90, 100) on a 1–5 Likert scale (from 1 = difficult, uncertain diagnosis to 5 = excellent, fully diagnostic). The best results were found at 60 keV

The ratings of gray and white matter differentiation, image noise, and the general usability for clinical purpose differed significantly (Friedman test: corrected *p*  0.0001). For gray-white matter differentiation, the 60 keV VMI were rated best with 3.98 ± 0.91 (see Fig. 4). In post hoc testing, the difference to the following 50 keV and 66 keV was also significant (corrected *p*  0.0001 in each case). For noise, the 66 keV VMI were rated best (3.99 ± 0.95). In post hoc testing both small differences to the 70 keV VMI the 60 keV VMI were significant. For general usability, the 60 keV VMI again performed best in the reading (4.09 ± 0.87), with significant differences also in post hoc testing (both corrected *p*  0.0001).

## Discussion

This study systematically evaluated virtual monoenergetic imaging and quantum iterative reconstruction for optimal brain tissue depiction in nonenhanced photon counting CT of the head.

In the literature there are only two publications which investigated the image quality of NCTH with photon counting CT. Pourmorteza et al. [15] evaluated the image quality of a preclinical PCCT in comparison to a conventional CT scanner with energy integrating detector. Here, the quality of the VMI was not investigated. In the recent study by Michael et al. [10] a selection of VMI was compared with polyenergetic reconstruction (PER), which seemed to show advantages of VMI images for the enhanced depiction of gray-white matter contrasts. The further development of polyenergetic reconstructions, which lacks algorithms for the reduction of beam hardening artifacts, is currently not foreseeable and may be neglected in favor of the development of VMI by the manufacturer. Thus, the focus remained on the VMI.

The simple description of contrast and noise failed to provide conclusive information on image quality that allowed to provide a matching subjective and objective image quality in earlier studies [12]. Thus, we decided to provide a more comprehensive analysis of the VMI spectrum in PCCT performed in this study. This provided detailed approach is novel with respect to the detail of quantification and proved to provide a paramount depiction of image quality. Most importantly, the quantitative results of the more complex analysis allowed reflection of the detail necessary to be in line with the subjective results. Following the results of our study, the 60 keV and 66 keV VMIs can now be objectively considered as optimal VMIs for general clinical use, the results of quantitative analysis and the reading provided conclusive and similar results in this respect. Overall, the optimal keV spectrum was comparable to dual energy methods as reported for dual layer CT [12] and dual source CT [13], so that the basic observations of VMI in dual energy CT appear to apply to PCCT as well. Whether separate VMIs can be helpful for individual pathologies such as acute ischemia or hemorrhage needs to be investigated in follow-up studies.

The quantum iterative reconstruction in PCCT has been examined in studies for low dose ultrahigh-resolution CT of the lung [16], for abdominal imaging in general [17] and specifically for the detection of liver lesions [18], and also for the hip [19]. No data are currently available for head and neck imaging. The data presented here show that QIR provides a significant reduction of noise with each level of QIR in the low keV range.

Artifacts caused by the cranial bone have always been a challenge in CT imaging of the skull, especially in the posterior fossa between the petrous bones. In their work on image quality of VMI in dual layer CT, Neuhaus et al. also proposed an artifact index for the area directly below the cranial calvaria [12]. A more extensive analysis is not available; however, the relationship between the signal and the distance to the cranial calvaria is also evident in the figures in Neuhaus et al. [12]. In the current study we examined the influence of the cranial calvaria on signal and noise of the brain parenchyma which was provided as a function of distance from the cranial calvaria. This approach is novel. Previous studies mainly referred to the artifacts of the posterior fossa (e.g., [13, 20]).

In the difficult space of the brain parenchyma up to 20 mm below the cranial calvaria, the results of this study show a significant artificial increase of the signal in low keV VMI. The noise, on the other hand, is increased only immediately below the cranial calvaria. The signal increase is particularly pronounced in low keV VMI and leads to an inhomogeneous representation of the brain parenchyma. As gray and white matter are equally affected by the signal increase, CNR tends to be lower near the cranial calvaria. These glaring artifacts limit the use of low keV reconstructions and oppose the use of the full high contrast low keV spectrum. The consideration of this phenomenon could be helpful in the development of algorithms for homogenization of the parenchyma representation. The artefacts below the calvaria emphasize that quantitative assessments of head CT need to reflect the gray and white matter with ROIs depending on their position and not, as usually conducted in other studies, as combined measures.

The limitations of the present study lie in the retrospective design. It is also not possible to answer which reconstructions are optimal for the detection of distinct pathologies. Here, our study can only provide a starting point for further investigations. We conclude that in nonenhanced PCCT of the head VMI provided the best image quality with 60–66 keV. Glaring artifacts below the calvaria limit the use of low keV monoenergetic images (40–55 keV). Quantitative iterative reconstructions (QIR) effectively reduced image noise in the brain without limitation of image quality, providing the best results in the highest QIR reconstruction modes. Our proposed quantitative method for the assessment of image quality within brain tissue correlated well with the subjective analysis of the independent readers.

### Supplementary Information

In the supplementary material, further and very detailed presentations of the analyses shown in the article are offered. In additional tables, all calculated parameters of the image quality for each ROI can also be accessed.
