# Supplementary material for: Nonenhanced Photon Counting CT of the Head: Impact of the keV Level, Iterative Reconstruction and Calvaria on Image Quality in Monoenergetic Images
Source: Clin Neuroradiol. 2023 Aug 17;34(1):75–83. doi: 10.1007/s00062-023-01331-w (PMC10881631; doi:10.1007/s00062-023-01331-w)
Supplement: Supplementary file 1 — In the supplementary material, further and very detailed presentations of the analyses shown in the article are offered. In additional tables, all calculated parameters of the image quality for each ROI can also be accessed. [file 62_2023_1331_MOESM1_ESM.docx]

This document provides Supplemental Material for the paper titled:

**Nonenhanced Photon Counting CT of the Head: Impact of the keV Level, Iterative Reconstruction and Calvaria on Image Quality in Monoenergetic Images**

# Abbreviations

| PCCT | Photon Counting Computed Tomography |
| --- | --- |
| DECT | Dual Energy Computed Tomography |
| VMI | Virtual Monoenergetic Image |
| QIR | Quantum Iterative Reconstruction |

| gray_subc | ROI (1): cortical gray matter below the calvaria |
| --- | --- |
| gray_5mm | ROI (2): cortical gray matter 5 mm below the calvaria |
| white_5mm | ROI (3): cortical white matter 5 mm below the calvaria |
| gray_10mm | ROI (4): cortical gray matter 10 mm below the calvaria |
| white_10mm | ROI (5): cortical white matter 10 mm below the calvaria |
| gray_15mm | ROI (6): cortical gray matter 15 mm below the calvaria |
| white_15mm | ROI (7): cortical white matter 15 mm below the calvaria |
| gray_20mm | ROI (8): cortical gray matter 20 mm below the calvaria |
| white_20mm | ROI (9): cortical white matter 20 mm below the calvaria |
| gray_caputhigh | ROI (10): gray matter in the superior caudate head |
| white_anthigh | ROI (11): white matter adjacent to ROI (6) in the superior internal capsule |
| gray_caputlow | ROI (12): gray matter in the inferior caudate head |
| white_antlow | ROI (13): white matter adjacent to ROI (8) in the anterior callosum |
| gray_thal | ROI (14): gray matter in the posterior thalamus |
| white_post | ROI (15): white matter in the posterior internal capsule |
| pons | ROI (16): white matter in the pons between the petrous bones |
| Q0 | Level 0 of QIR: no iterative reconstruction |
| Q1 | Level 1 of QIR |
| Q2 | Level 2 of QIR |
| Q3 | Level 3 of QIR |
| Q4 | Level 4 of QIR, maximal iterative reconstruction |
| cnr_cortical_subc | CNR directly below the calvaria: gray matter ROI (1) and white matter ROI (3) |
| cnr_cortical_5mm | CNR 5 mm below the calvaria: gray matter ROI (2) and white matter ROI (3) |
| cnr_cortical_10mm | CNR 10 mm below the calvaria: gray matter ROI (4) and white matter ROI (5) |
| cnr_cortical_15mm | CNR 15 mm below the calvaria: gray matter ROI (6) and white Matter ROI (7) |
| cnr_cortical_20mm | CNR 20 mm below the calvaria: gray matter ROI (8) and white Matter ROI (9) |
| cnr_caput_high | CNR at the superior caudate head: gray matter ROI (10) and white Matter ROI (11) |
| cnr_caput_low | CNR at the inferior caudate head: gray matter ROI (12) and white Matter ROI (13) |
| cnr_thalamus | CNR at the thalamus: gray matter ROI (14) and white Matter ROI (15) |
|  |  |

# Material and Methods

**In this section a more detailed description of material and methods with corresponding figures is provided.**

### 3.1 Patient Population

Institutional review board approval was obtained. Informed consent was waived due to the retrospective study design. All patients who had undergone nonenhanced CT of the head at our institution between July and October 2022 were identified. Each CT was performed with a clinical protocol and with medical indication. To exclude confounders, patients with acute or chronic intracranial disease diagnosed on CCT were excluded; these criteria included intracranial hemorrhages, infarcts, masses, extensive leukoencephalopathy, edema of any type, and implants/foreign objects. Similarly, examinations with motion artifacts were excluded. The data of the included patients were anonymized.

### 3.2 CT Protocol and Image Acquisition

All CT scans were performed using the clinically-approved photon-counting CT (NAEOTOM Alpha, software version Syngo CT VA50, Siemens Healthineers, Erlangen, Germany) with a predefined spiral CT protocol. All patients were examined in supine position with moderate flexion in the cervical spine to perform axial acquisition in the orbitomeatal plane. After the topogram was made, a lens shield was applied. Single collimation was 0.4 mm, total collimation 38.4 mm, and pitch factor was 0.55 with a rotation time of 0.5 s. Tube voltage was 120 kVp, and tube current was modulated due to the manufacturer’s program of dose modulation. The matrix size was 512 × 512, and the field of view (FOV) was optimized for the individual head size. The reconstruction kernel QR36 was used for the spectral data sets. The primary data were further processed without and with all possible levels of Quantum Iterative Reconstruction (QIR, levels 0 – 4); the current clinically used beam-hardening reduction algorithm was automatically applied. The manufacturer-specific spectral workstation (Syngo.Via, VB60_B version, Siemens Healthineers, Erlangen, Germany) was used to analyze the datasets. The images were reconstructed in axial view with a slice thickness of 3 mm and a slice increment of 3 mm.

### 3.3 Quantitative Image Analysis

Sixteen different ROIs were used for the quantitative data analysis; these included ROIs from similar preceding studies (Michael, Boriesosdick, Schoenbeck, Lopez-Schmidt, et al., 2022; Neuhaus et al., 2017). Nine ROIs involved the gray and white matter of the neocortex below the calvaria. At a location with an appropriate gyrus in terms of length and orientation and in a slice at least 20 mm above the skull base and up to just supraventricular, the diameter of the cranial calvaria was measured. Then the first ROI (1) was placed in the gray matter just below the calvaria. ROI (2) was placed in the gray matter 5 mm below the calvaria on an orthogonal running in the tangent of the calvaria at the location of the selected gyrus, and ROI (3) was placed in the adjacent white matter 5 mm below the calvaria. ROI (4) and (5) were analogously placed in the gray and white matter, respectively, at 10 mm from the cranial calvaria, ROI (6) and (7) at 15 mm, ROI (8) and (9) at 20 mm (figure 1).


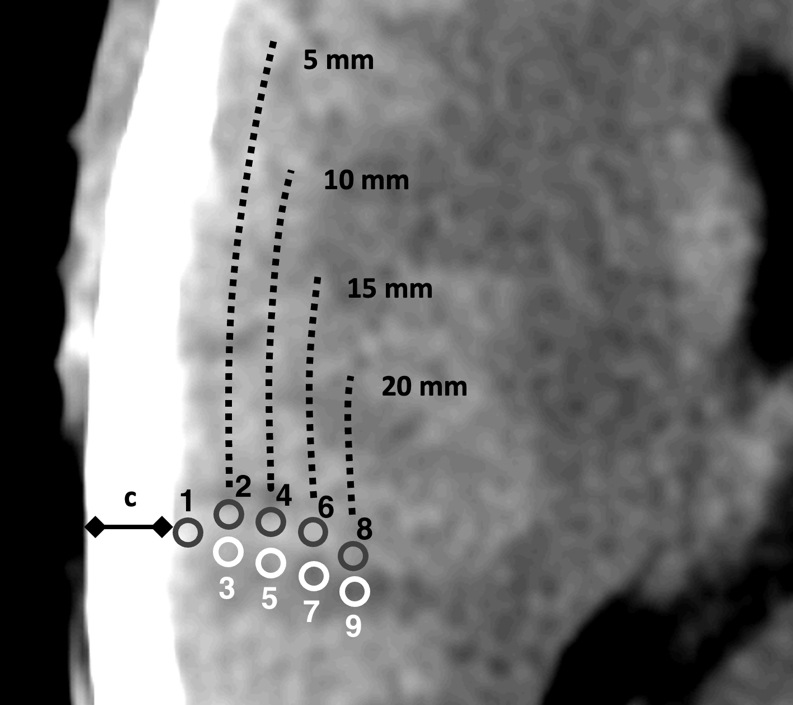


**Figure 1:** Position of the cortical ROIs (1) – (9). ROI (1) in the gray matter below the calvaria (c: calvaria thickness); ROI (2) in gray, ROI (3) in white matter 5 mm below the calvaria; ROI (4) in gray, ROI (5) in white matter 10 mm below the calvaria; ROI (6) and (7) 15 mm and ROI (8) and (9) 20 mm below the calvaria, respectively.

Six ROIs involved the basal ganglia, the thalamus and the immediately adjacent white matter in each case (figure 2). ROI (10) was placed in the superior caput of the caudate nucleus, ROI (11) in the immediately adjacent white matter in the anterior internal capsule. ROI (12) was placed in the inferior caudate head, ROI (13) in the immediately adjacent anterior callosum. ROI (14) was set in the posterior thalamus, ROI (15) in the immediately adjacent posterior internal capsule. Finally ROI (16) was placed in the pons between the petrous bones (figure 2) to provide a measure of artifacts analogous to the preliminary studies (Michael, Boriesosdick, Schoenbeck, Woeltjen, et al., 2022).


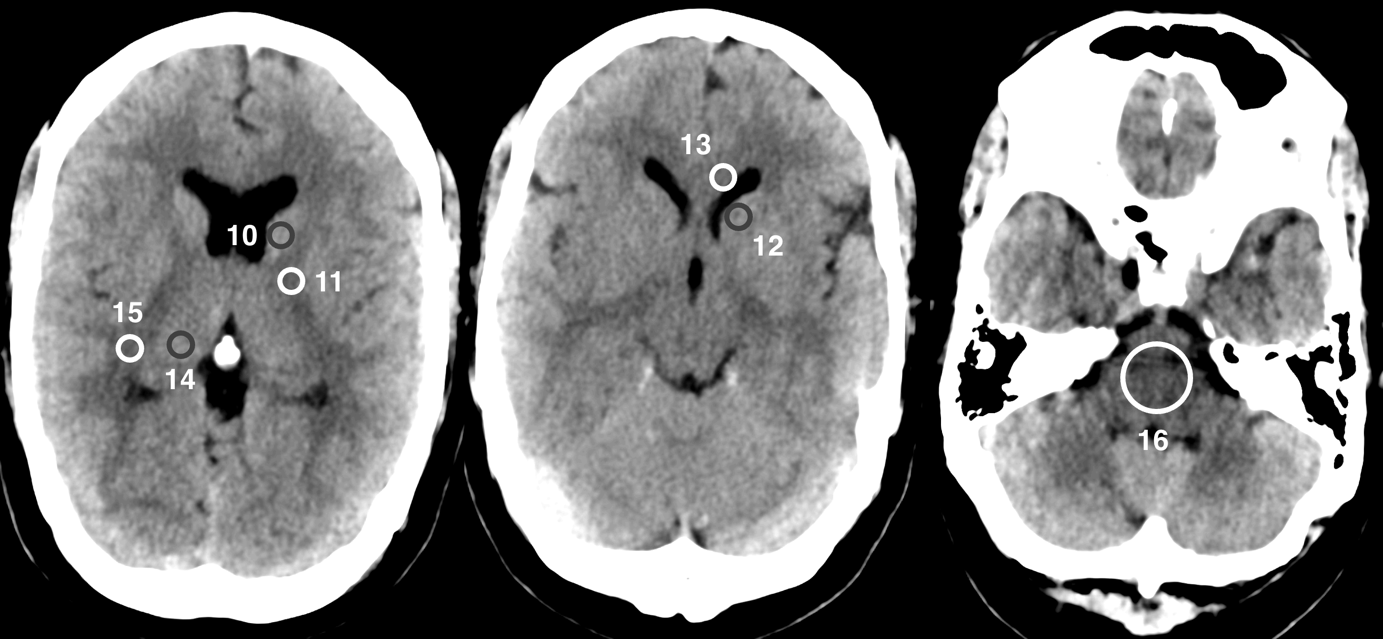


**Figure 2:** Position of ROI (10) to ROI (16). ROI (10) in the superior caput of the caudate nucleus, ROI (11) in the white matter immediately adjacent (e.g. anterior internal capsule); ROI (12) in the inferior caudate head, ROI (13) in the adjacent anterior callosum; ROI (14) in the posterior thalamus, ROI (15) in the adjacent posterior internal capsule; ROI (16) in the pons between the petrous bones.

The size of ROI (1) to ROI (15) measured 4 mm^2^. To ensure that only gray matter was in the ROI, especially for cortical matter, the ROI was downsized in very few cases. ROI (16) measured 200 mm^2^, analogous to the previous study (Michael, Boriesosdick, Schoenbeck, Woeltjen, et al., 2022).

Within the spectral data sets of all levels of the QIR (levels 0 – 4), each ROI was set identically. A total of 151 virtual monoenergetic reconstructions (VMI) with keV levels from 40 keV to 190 keV in 1-keV steps were prepared (for an exemplary selection see figure 3). For each ROI, both the average density and its standard deviation in Hounsfield Units were measured in every QIR level and every VMI.


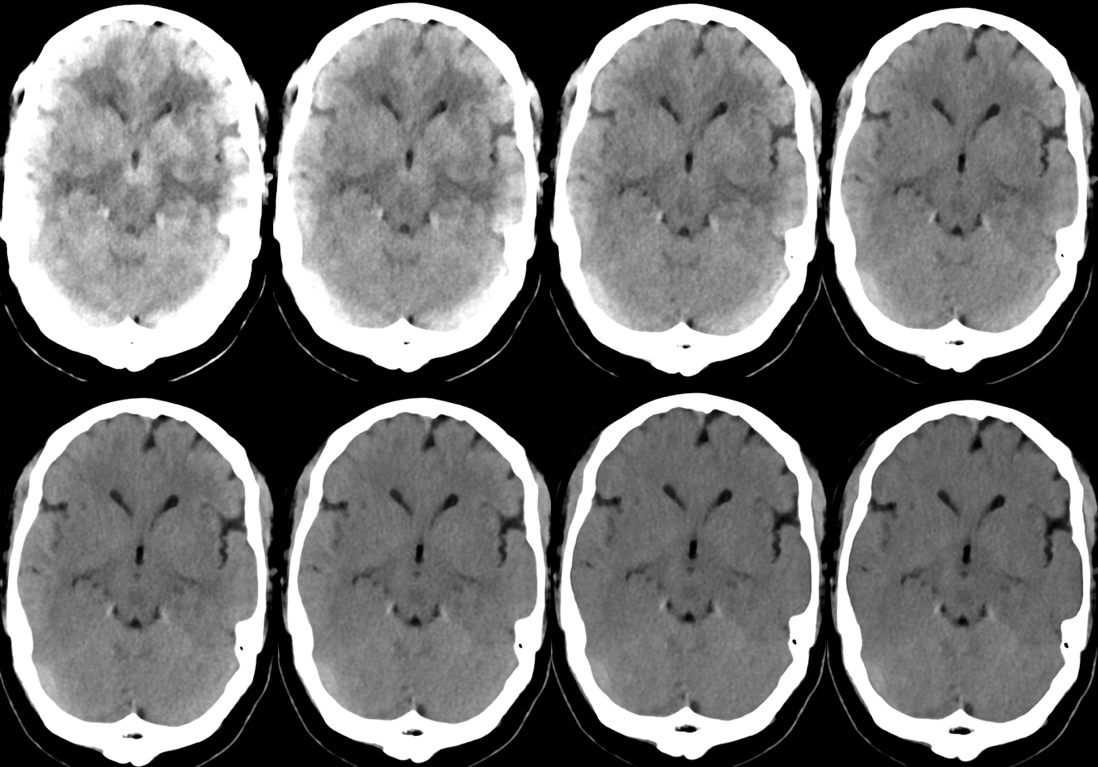


**Figure 3:** An exemplary selection of virtual monoenergetic images for an overview. From top left to bottom right: 40 keV, 50 keV, 60 keV, 70 keV, 80 keV, 90 keV, 120 keV, 150 keV. The window settings are in all cases C40/W70.

Parameters for assessing image quality were determined as described previously, in order to allow for comparison of study results (Michael, Boriesosdick, Schoenbeck, Woeltjen, et al., 2022; Pomerantz et al., 2013). The signal of the ROIs was defined as the mean density/attenuation in Hounsfield units (HU). Noise was defined as the standard deviation (SD) of a ROI in HU. The SD of the ROI in the pons between the petrous bones was referred to as the posterior fossa artifact index (PFAI) (Neuhaus et al., 2017). The signal-to-noise ratio (SNR) was calculated by dividing the mean density of the ROI (signal) by the corresponding SD of the ROI (noise). The contrast-to-noise ratio was calculated as the quotient of the difference of the mean density of two adjacent ROIs with gray matter (GM) and white matter (WM) and the square root of the sum of the variance of both ROIs (Michael, Boriesosdick, Schoenbeck, Woeltjen, et al., 2022):

$$CNR= \frac{{mean}_{GM}- {mean}_{WM}}{\sqrt{{SD}_{GM}^{2}+ {SD}_{WM}^{2}}}$$

### 3.4 Qualitative Image Analysis

The image quality of specific VMIs (keV: 40, 50, 60, 66, 70, 75, 90, 100, selected based on the quantitative results to include the VMI with the maximal signal and CNR) in QIR level 4 was evaluated by five radiologists: two experienced neuroradiologists with 17 and 12 years of experience, two experienced general radiologists with 8 and 6 years of experience and one resident with 2 years of experience. The quality of gray-white matter differentiation, the extent of noise and the general clinical usability were rated using 5-point Likert scales (from 1 = “difficult, uncertain diagnosis” to 5 = “excellent, fully diagnostic”). The five readers were blinded to the keV level and other used algorithms for postprocessing. Every VMI was presented with equal standard windowing using a commonly accepted setting (C40/W80), since there is currently no evidence for the optimal window settings of different VMI in nonenhanced CT of the head.

### 3.5 Additional Measurements

After evaluation of the quantitative measurements the influence of the cranial calvaria was investigated for its causality. For general assessment of calvarial artifacts we identified two patients with craniectomy shortly before reimplantation in the available period. In the unenhanced CCT of these two patients, 10 ROIs each with a size of 4 mm were placed on the side with cranial dome with a distance of approximately 5 to 10 mm; on the side of craniectomy, 10 ROIs were placed at 5 to 10 mm from the dura. In both hemispheres only brain parenchyma was included that was unharmed.

### 3.6 Statistical Analysis

Data processing and statistical analyses were performed using the statistical software R (Version 4.1.0; R Core Team, 2021) and RStudio (Version 2022.07.1+554). The Shapiro–Wilk test was applied to test for normal distribution, the Levene test to check for homoscedasticity. When considering the individual ROIs, a one-way ANOVA or the Friedman test as the corresponding nonparametric method was used to investigate whether the image parameters of the individual keV levels differed. Using the Bonferroni method, the resulting p values were corrected. For post hoc testing with selected keV levels, e.g. the keV level with the maximum or minimum value of a parameter and the adjacent keV levels as well as a representative selection of the spectrum, the paired T-test or the nonparametric Wilcoxon signed rank test were used. Again, the correction of the p values was done using the Bonferroni method.

For qualitative analysis, appropriate nonparametric procedures were used (Friedman test and Wilcoxon test). If not stated otherwise, all data are presented mean ± standard deviation.

# Results: Analysis of Signal, Noise, SNR and CNR

In the coming sections, the results of the analyses of the individual parameters of the image quality are shown. First, the signal is presented, then the noise; after a short presentation of the Signal to Noise Ratio, the results regarding the Contrast to Noise Ratio are finally presented.

## Signal

The signal is shown for each ROI individually as a function of the keV level of the VMI and the iterative reconstruction (Q level). This general presentation is followed by a synopsis of several ROIs to highlight commonalities and differences. In particular, the influence of the calvaria is elaborated.

## Noise

The noise is analyzed and presented analogously to the signal. After presenting the noise in each ROI as a function of Q level and keV, ROIs are again compiled to illustrate similarities and differences. Again, the influence of the calvaria is analyzed.

## Signal-to-Noise-Ratio (SNR)

The Signal to Noise Ratio (SNR) is analyzed for each ROI analogously to Signal and Noise. Due to the importance and clarity of the CNR, further analyses are primarily omitted.

## Contrast-to-Noise-Ratio (CNR)

The Contrast to Noise Ratio is one of the most illustrative and important image parameters in unenhanced CT imaging of the head. After all, a large part of all diagnosis is based on the differentiation of gray and white matter, furthermore other tissue types - this differentiation is of course based on the contrast, which should have as large a ratio as possible to the accompanying noise, so that the image diagnosis can be sensitive and specific.

The CNR is first plotted as a function of keV and Q level for each ROI, analogous to the previous parameters. Then a compilation of different ROIs with further aspects follows.

# Results: First Approach to the Signal

## ROI (1): Gray Matter directly below the Calvaria


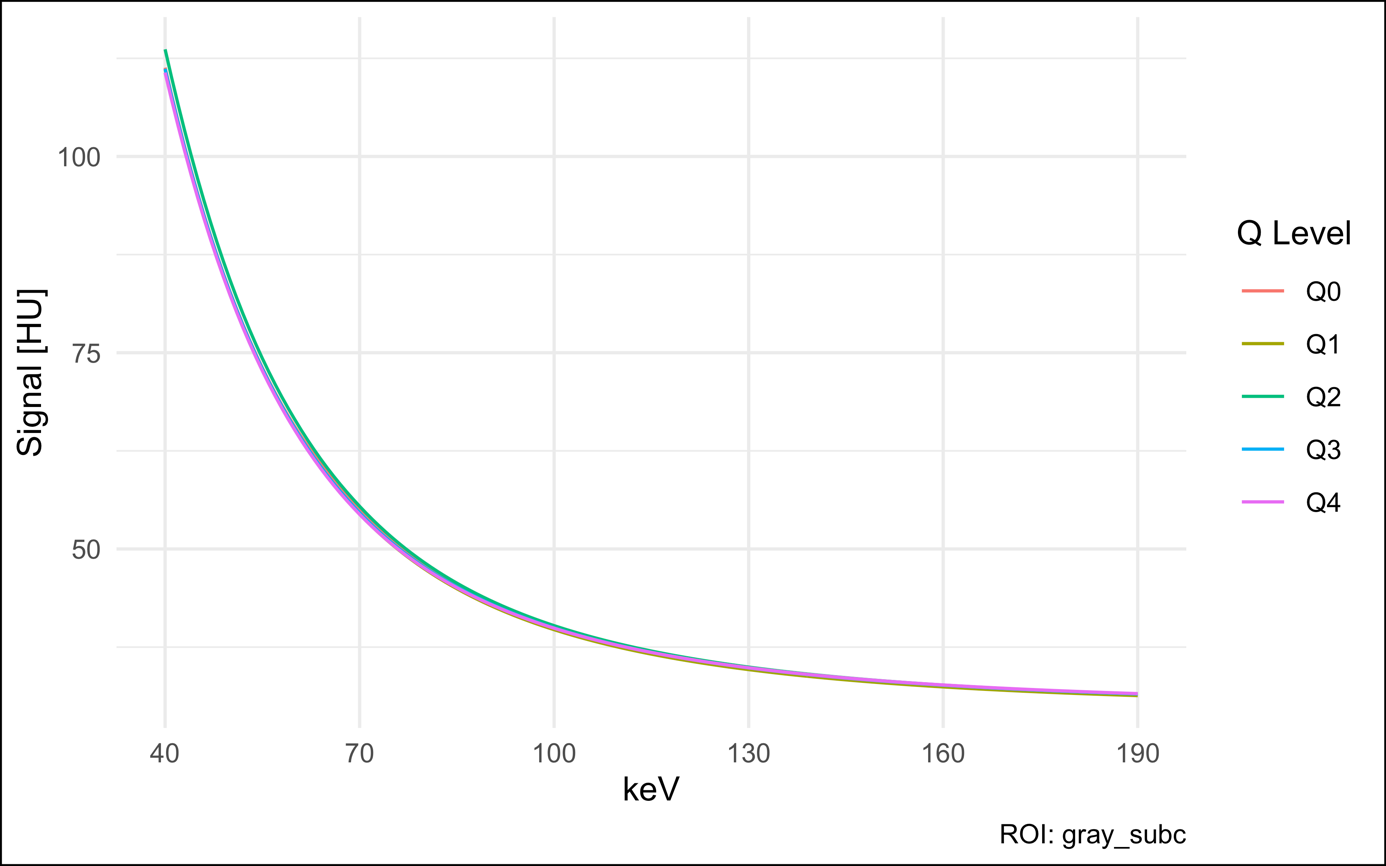


There is a significant difference between the signal characteristics of the individual virtual monoenergetic reconstructions (Friedman test corrected p < 0.0001) in ROI (1). The maximum gray matter signal immediately below the calvaria is found in the 40 keV VMI and averages 110.68 ± 31.20 HU in Q4 exemplarily. The differences between the individual Q levels are minimal and do not seem relevant. In post hoc testing, a significant difference in comparison to the next keV level of 41 keV is found (corrected p < 0.0001).

Selected keV levels with all Q levels and their post hoc tests can be reviewed in the supplemental material (Excel file “PCCT_CCT_Analysis.xlsx”, sheet “analysis_signal”).

## ROI (2): Cortical Gray Matter 5 mm below the Calvaria


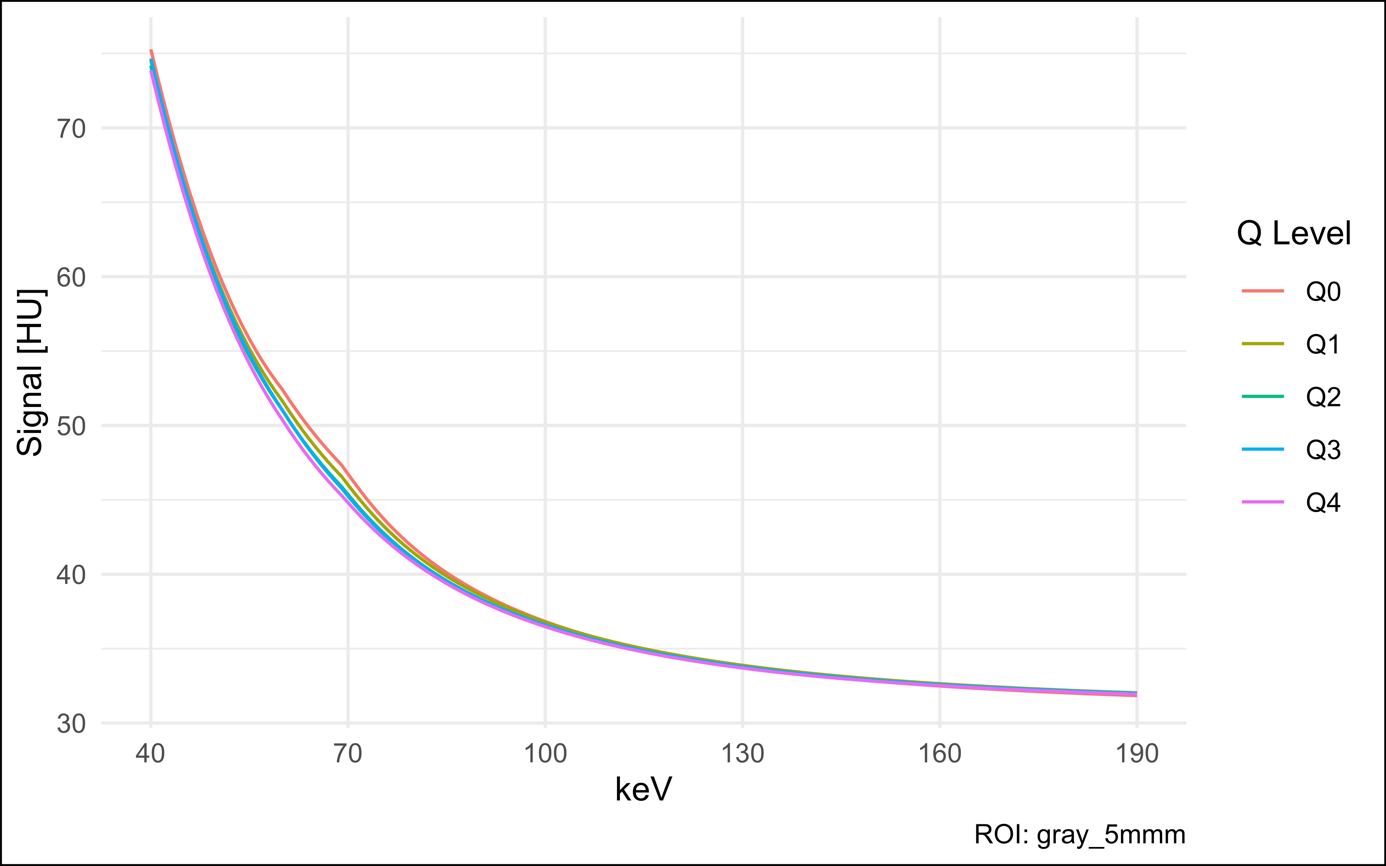


There is a significant difference between the signal characteristics of the individual virtual monoenergetic reconstructions (Friedman test corrected p < 0.0001) in ROI (2). The maximum gray matter signal 5 mm below the calvaria is found in the 40 keV VMI and averages 73.86 ± 10.75 HU in Q4 exemplarily. The differences between the individual Q levels are minimal and do not seem relevant. In post hoc testing, a significant difference in comparison to the next keV level of 41 keV is found (corrected p < 0.0001).

Selected keV levels with all Q levels and their post hoc tests can be reviewed in the supplemental material (Excel file “PCCT_CCT_Analysis.xlsx”, sheet “analysis_signal”).

## ROI (3): Cortical White Matter 5 mm below the Calvaria


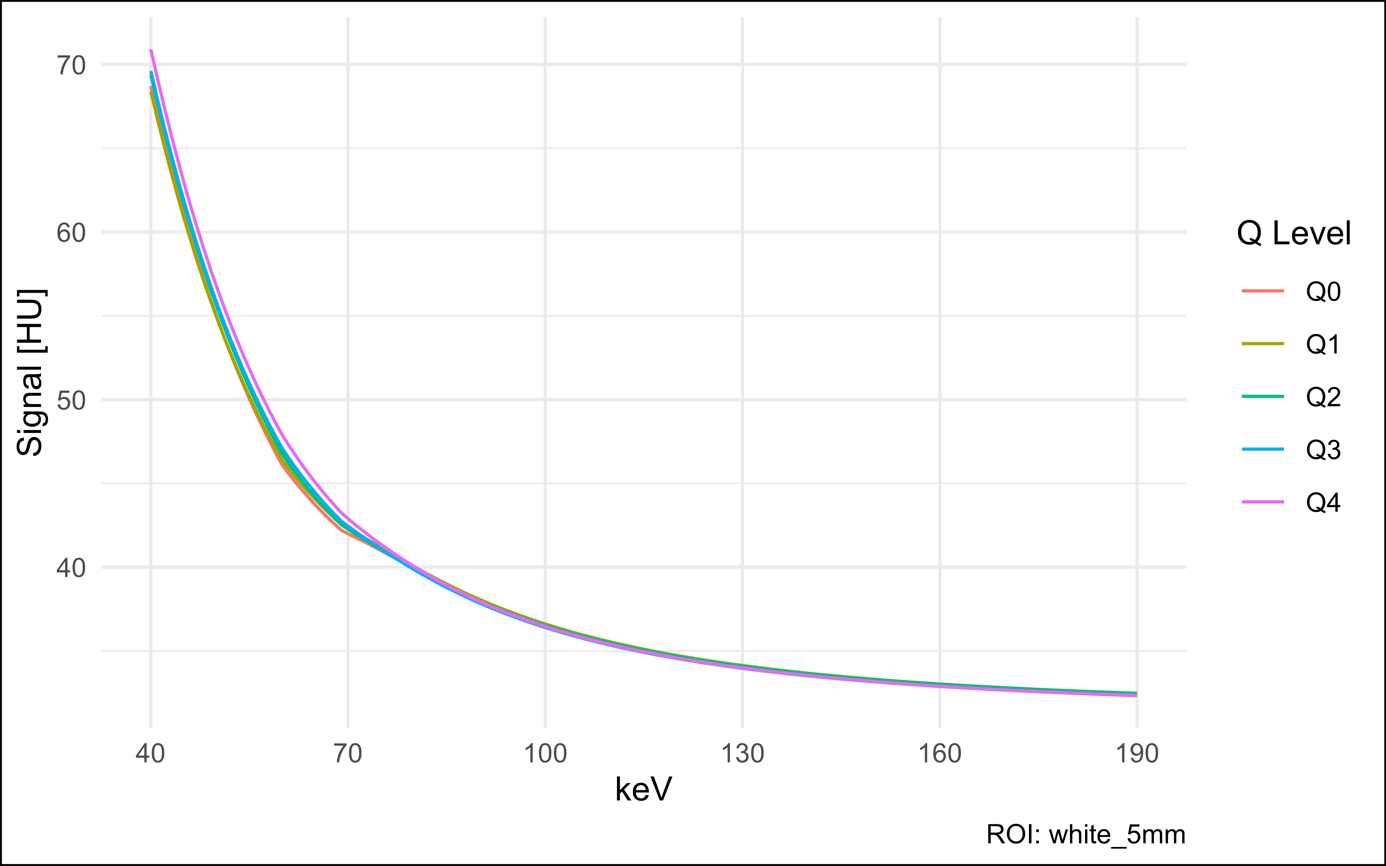


There is a significant difference between the signal characteristics of the individual virtual monoenergetic reconstructions (Friedman test corrected p < 0.0001) in ROI (3). The maximum white matter signal 5 mm below the calvaria is found in the 40 keV VMI and averages 70.91 ± 10.70 HU in Q4 exemplarily. The differences between the individual Q levels are minimal and do not seem relevant. In post hoc testing, a significant difference in comparison to the next keV level of 41 keV is found (corrected p < 0.0001).

Selected keV levels with all Q levels and their post hoc tests can be reviewed in the supplemental material (Excel file “PCCT_CCT_Analysis.xlsx”, sheet “analysis_signal”).

## ROI (4): Cortical Gray Matter 10 mm below the Calvaria


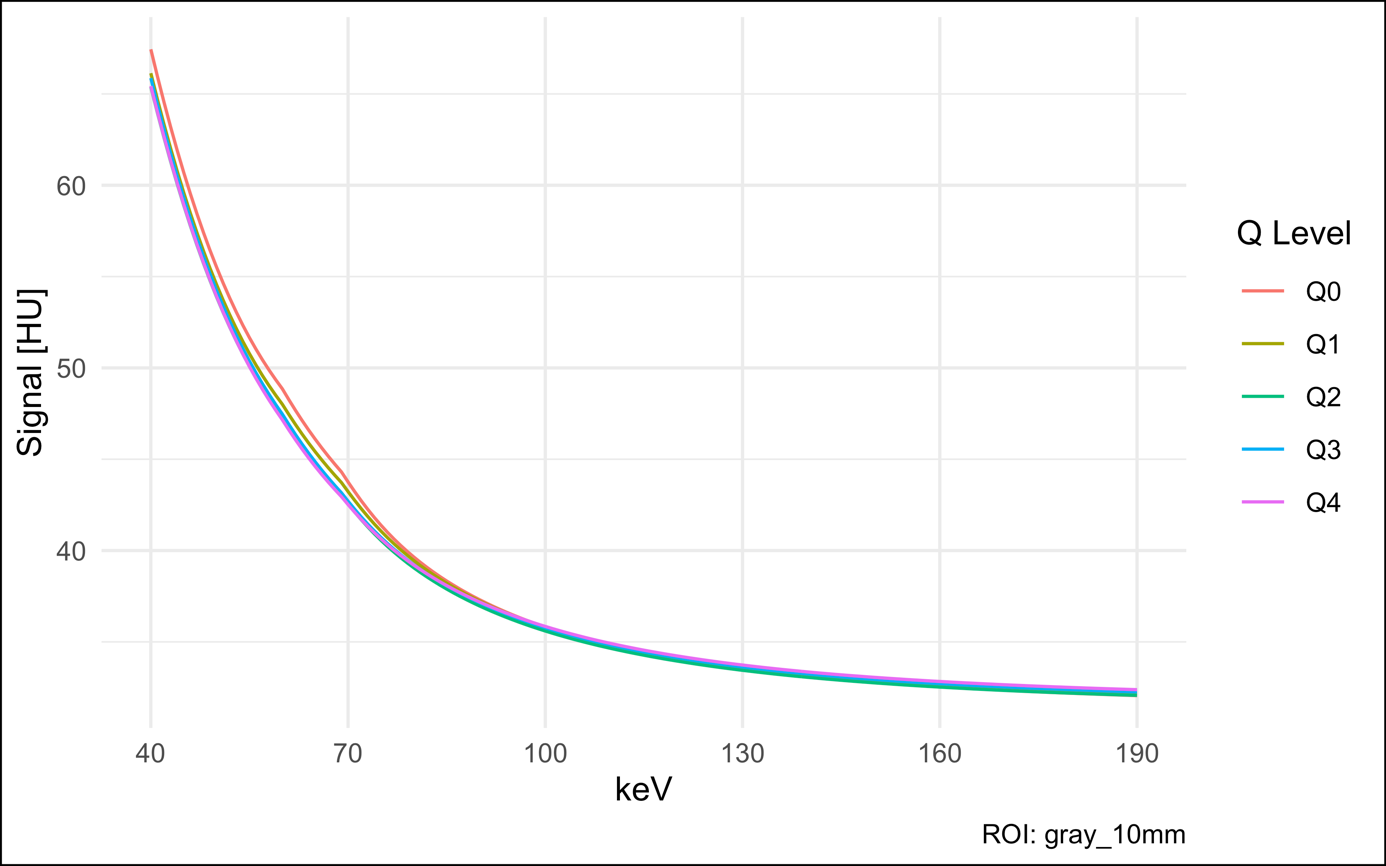


There is a significant difference between the signal characteristics of the individual virtual monoenergetic reconstructions (Friedman test corrected p < 0.0001) in ROI (4). The maximum gray matter signal 10 mm below the calvaria is found in the 40 keV VMI and averages 65.43 ± 8.40 HU in Q4 exemplarily. The differences between the individual Q levels are minimal and do not seem relevant. In post hoc testing, a significant difference in comparison to the next keV level of 41 keV is found (corrected p < 0.0001).

Selected keV levels with all Q levels and their post hoc tests can be reviewed in the supplemental material (Excel file “PCCT_CCT_Analysis.xlsx”, sheet “analysis_signal”).

## ROI (5): White Matter 10 mm below the Calvaria


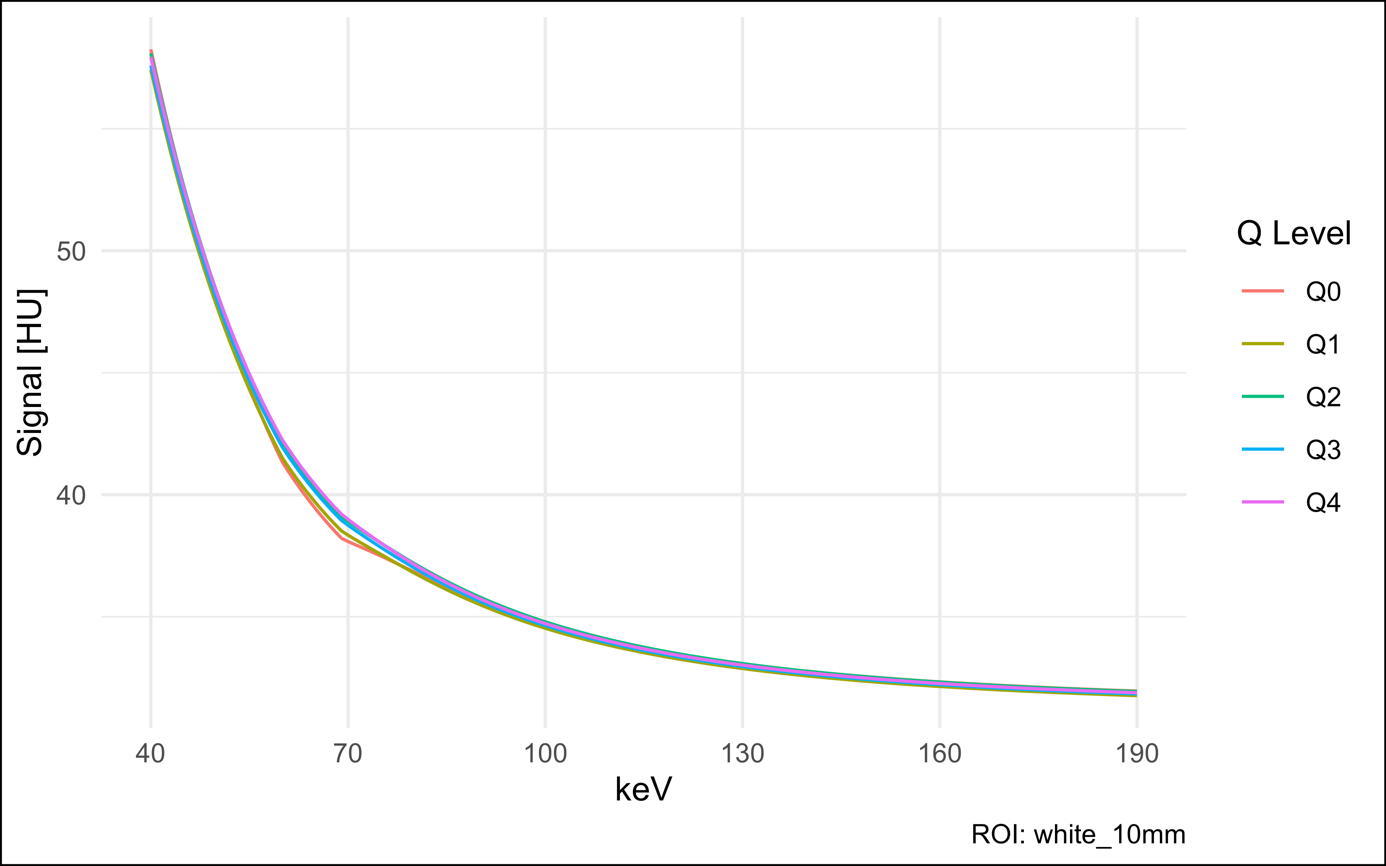


There is a significant difference between the signal characteristics of the individual virtual monoenergetic reconstructions (Friedman test corrected p < 0.0001) in ROI (5). The maximum white matter signal 10 mm below the calvaria is found in the 40 keV VMI and averages 57.94 ± 8.38 HU in Q4 exemplarily. The differences between the individual Q levels are minimal and do not seem relevant. In post hoc testing, a significant difference in comparison to the next keV level of 41 keV is found (corrected p < 0.0001).

Selected keV levels with all Q levels and their post hoc tests can be reviewed in the supplemental material (Excel file “PCCT_CCT_Analysis.xlsx”, sheet “analysis_signal”).

## ROI (6): Cortical Gray Matter 15 mm below the Calvaria


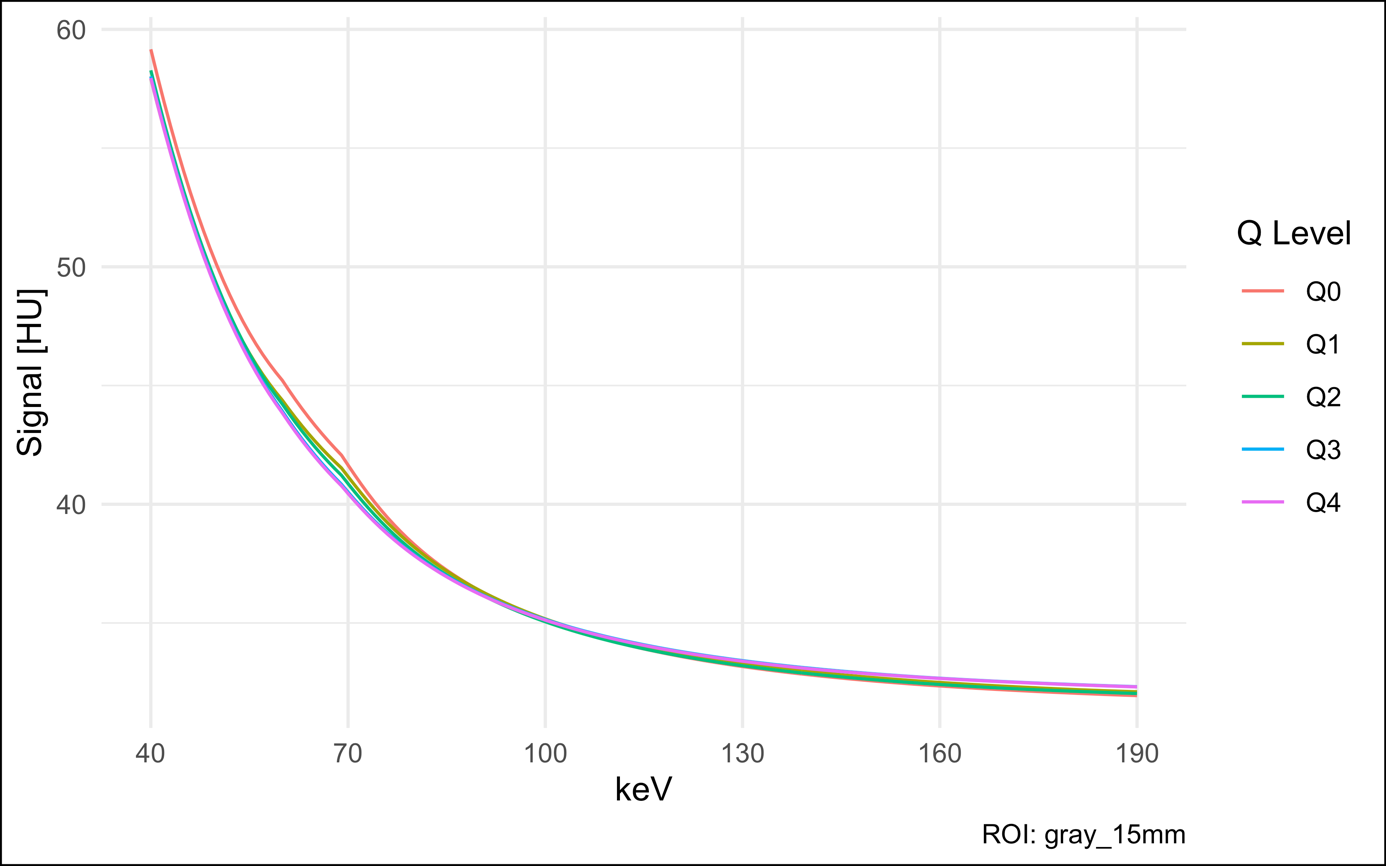


There is a significant difference between the signal characteristics of the individual virtual monoenergetic reconstructions (Friedman test corrected p < 0.0001) in ROI (6). The maximum gray matter signal 15 mm below the calvaria is found in the 40 keV VMI and averages 57.95 ± 6.62 HU in Q4 exemplarily. The differences between the individual Q levels are minimal and do not seem relevant. In post hoc testing, a significant difference in comparison to the next keV level of 41 keV is found (corrected p < 0.0001).

Selected keV levels with all Q levels and their post hoc tests can be reviewed in the supplemental material (Excel file “PCCT_CCT_Analysis.xlsx”, sheet “analysis_signal”).

## ROI (7): Cortical White Matter 15 mm below the Calvaria


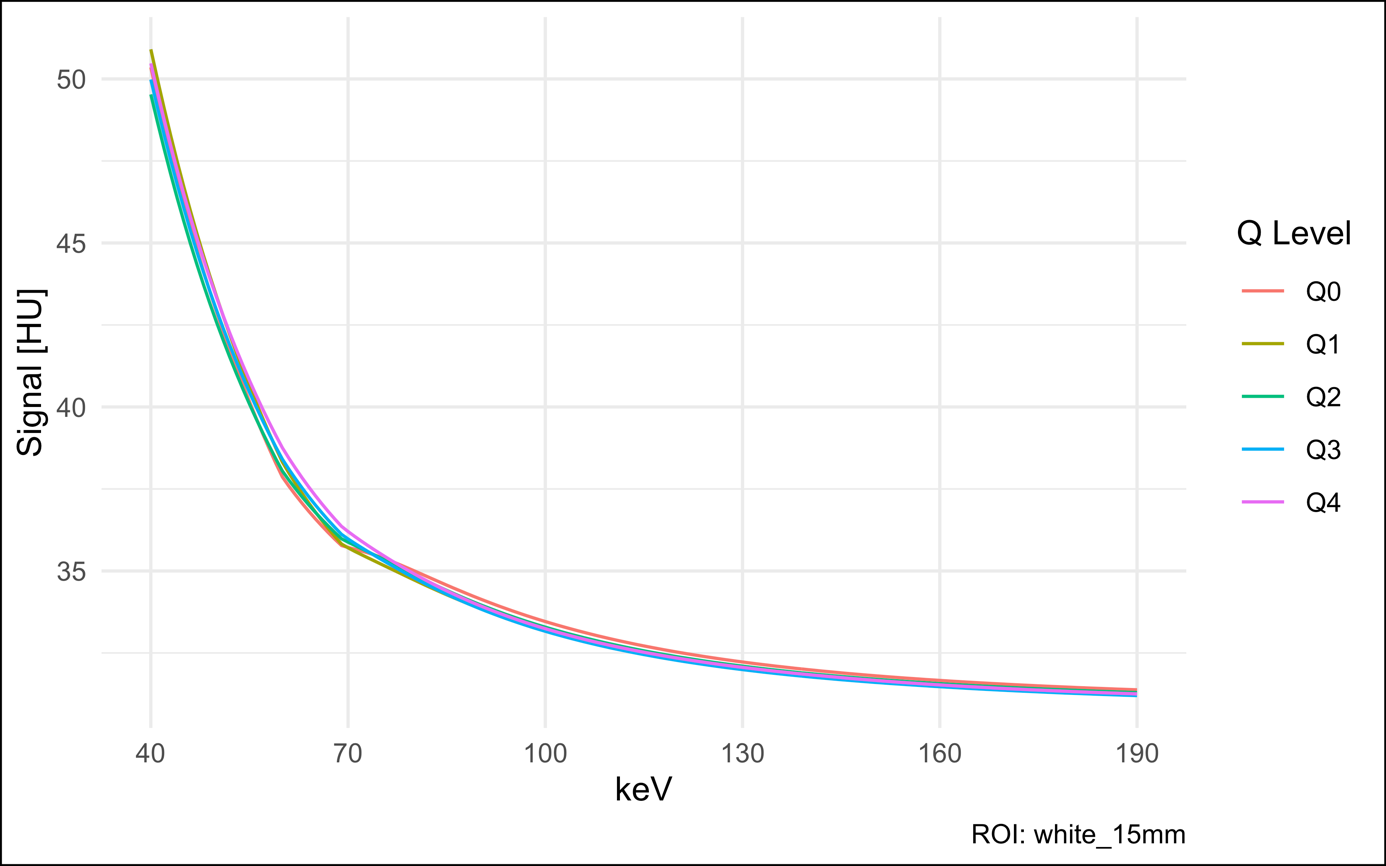


There is a significant difference between the signal characteristics of the individual virtual monoenergetic reconstructions (Friedman test corrected p < 0.0001) in ROI (7). The maximum white matter signal 15 mm below the calvaria is found in the 40 keV VMI and averages 50.48 ± 6.17 HU in Q4 exemplarily. The differences between the individual Q levels are minimal and do not seem relevant. In post hoc testing, a significant difference in comparison to the next keV level of 41 keV is found (corrected p < 0.0001).

Selected keV levels with all Q levels and their post hoc tests can be reviewed in the supplemental material (Excel file “PCCT_CCT_Analysis.xlsx”, sheet “analysis_signal”).

## ROI (8): Cortical Gray Matter 20 mm below the Calvaria


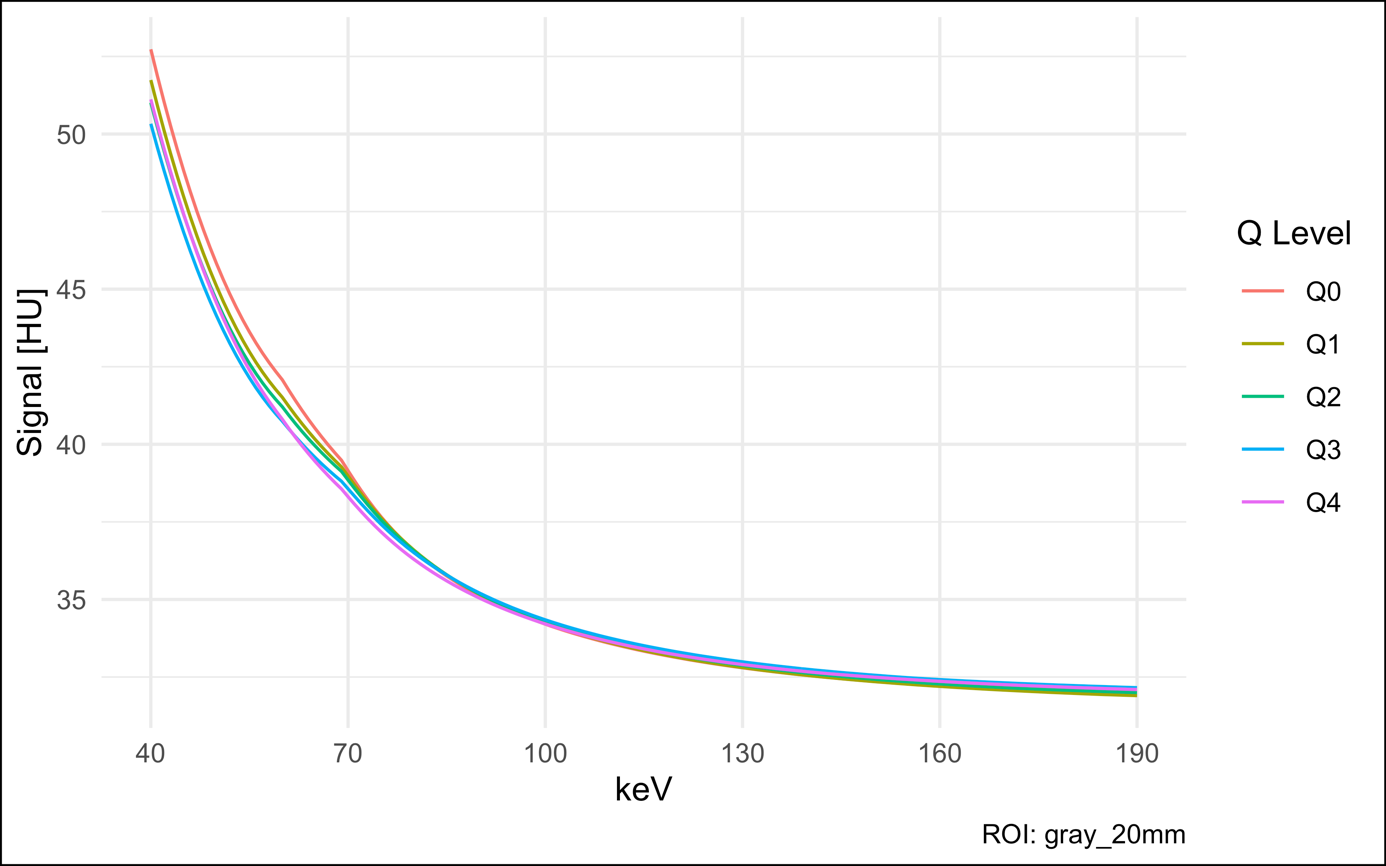


There is a significant difference between the signal characteristics of the individual virtual monoenergetic reconstructions (Friedman test corrected p < 0.0001) in ROI (8). The maximum gray matter signal 20 mm below the calvaria is found in the 40 keV VMI and averages 51.12 ± 6.20 HU in Q4 exemplarily. The differences between the individual Q levels are minimal and do not seem relevant. In post hoc testing, a significant difference in comparison to the next keV level of 41 keV is found (corrected p < 0.0001).

Selected keV levels with all Q levels and their post hoc tests can be reviewed in the supplemental material (Excel file “PCCT_CCT_Analysis.xlsx”, sheet “analysis_signal”).

## ROI (9): Cortical White Matter 20 mm below the Calvaria


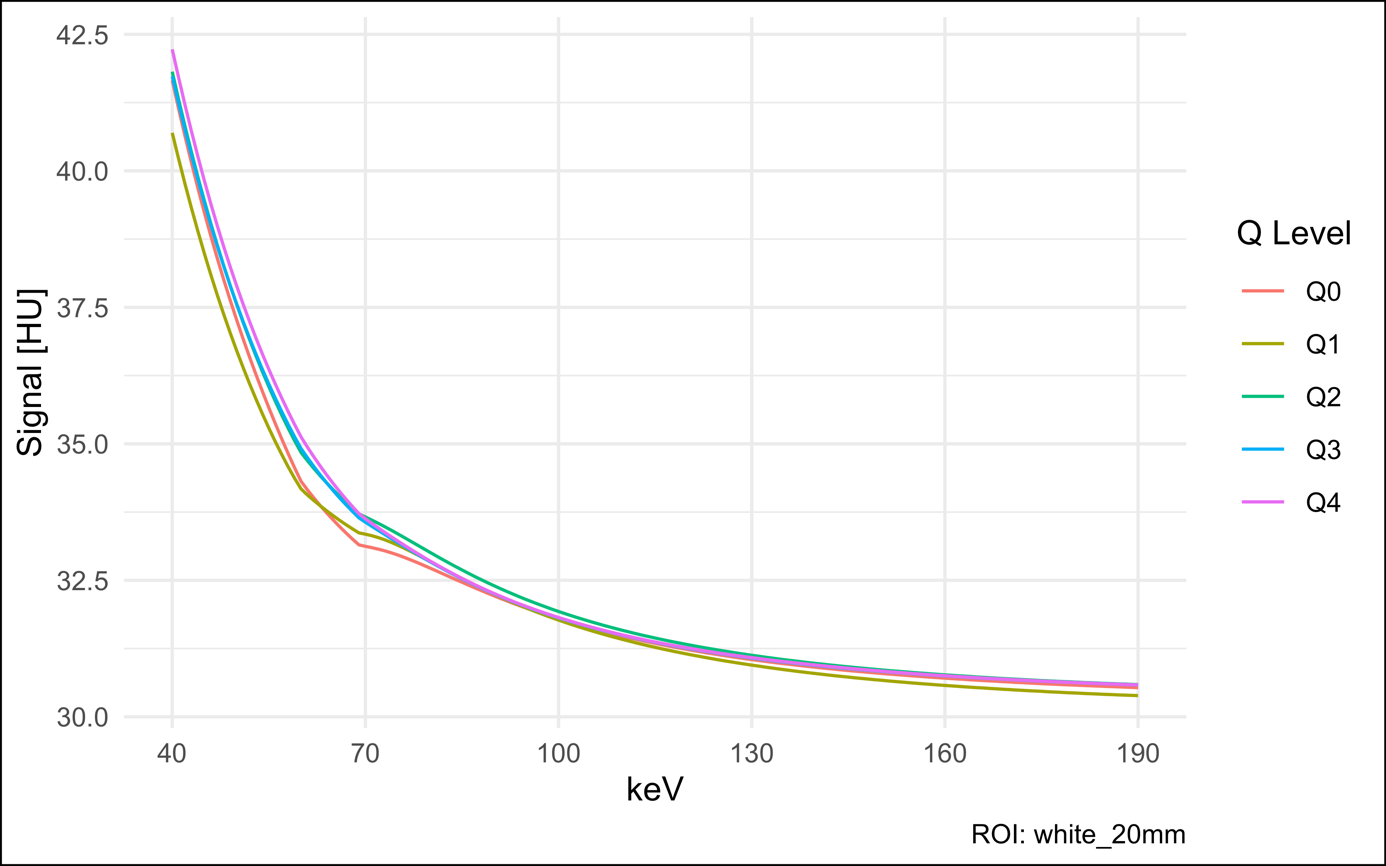


There is a significant difference between the signal characteristics of the individual virtual monoenergetic reconstructions (Friedman test corrected p < 0.0001) in ROI (9). The maximum white matter signal 20 mm below the calvaria is found in the 40 keV VMI and averages 42.23 ± 6.91 HU in Q4 exemplarily. The differences between the individual Q levels are minimal and do not seem relevant. In post hoc testing, a significant difference in comparison to the next keV level of 41 keV is found (corrected p < 0.0001).

Selected keV levels with all Q levels and their post hoc tests can be reviewed in the supplemental material (Excel file “PCCT_CCT_Analysis.xlsx”, sheet “analysis_signal”).

## ROI (10): Gray Matter in the Superior Caput Nuclei Caudati


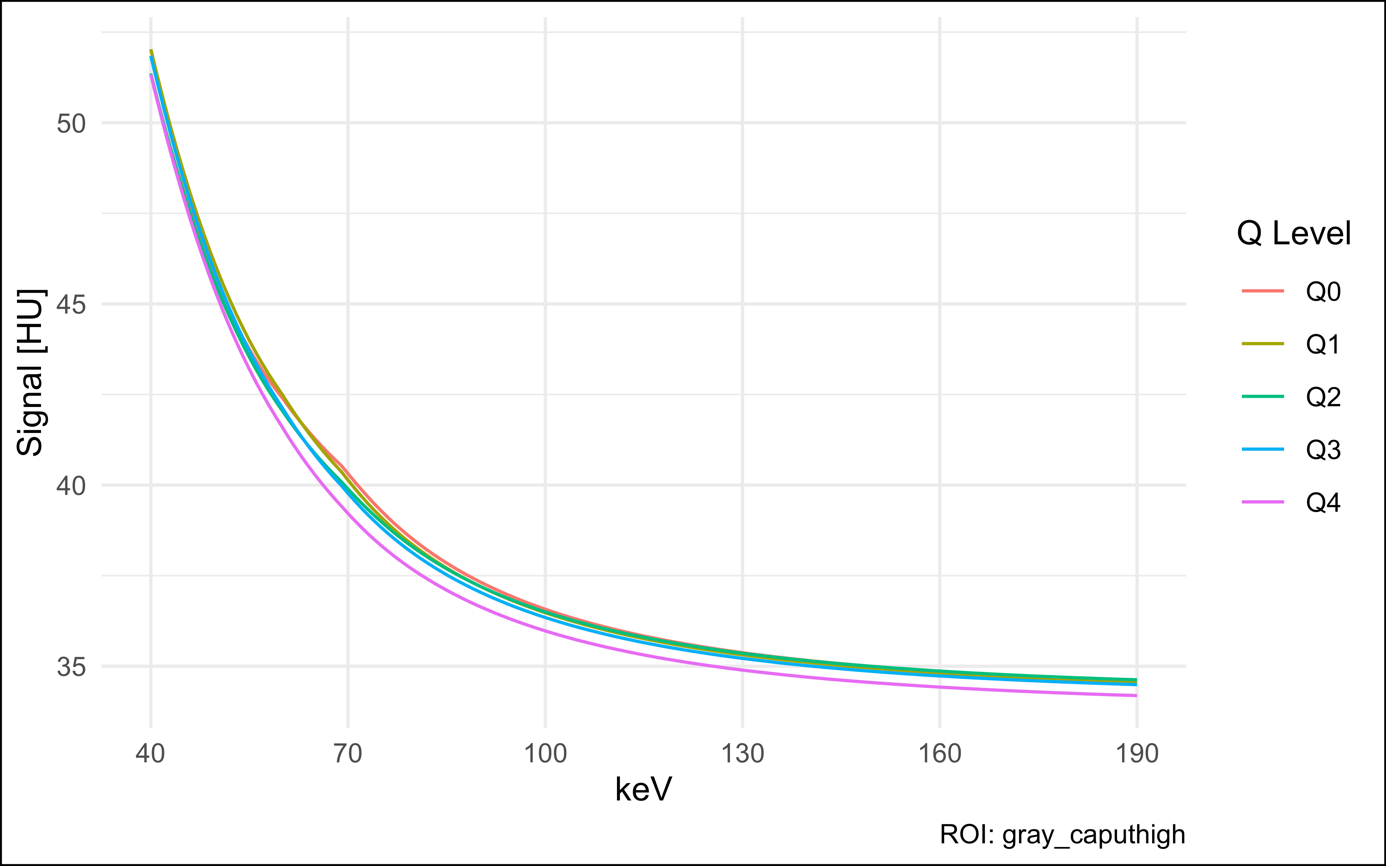


There is a significant difference between the signal characteristics of the individual virtual monoenergetic reconstructions (Friedman test corrected p < 0.0001) in ROI (10). The maximum gray matter signal in the superior caput nuclei caudati is found in the 40 keV VMI and averages 51.33 ± 4.54 HU in Q4 exemplarily. The differences between the individual Q levels are minimal and do not seem relevant. In post hoc testing, a significant difference in comparison to the next keV level of 41 keV is found (corrected p < 0.0001).

Selected keV levels with all Q levels and their post hoc tests can be reviewed in the supplemental material (Excel file “PCCT_CCT_Analysis.xlsx”, sheet “analysis_signal”).

## ROI (11): White Matter adjacent to ROI (10) in the Superior Internal Capsule


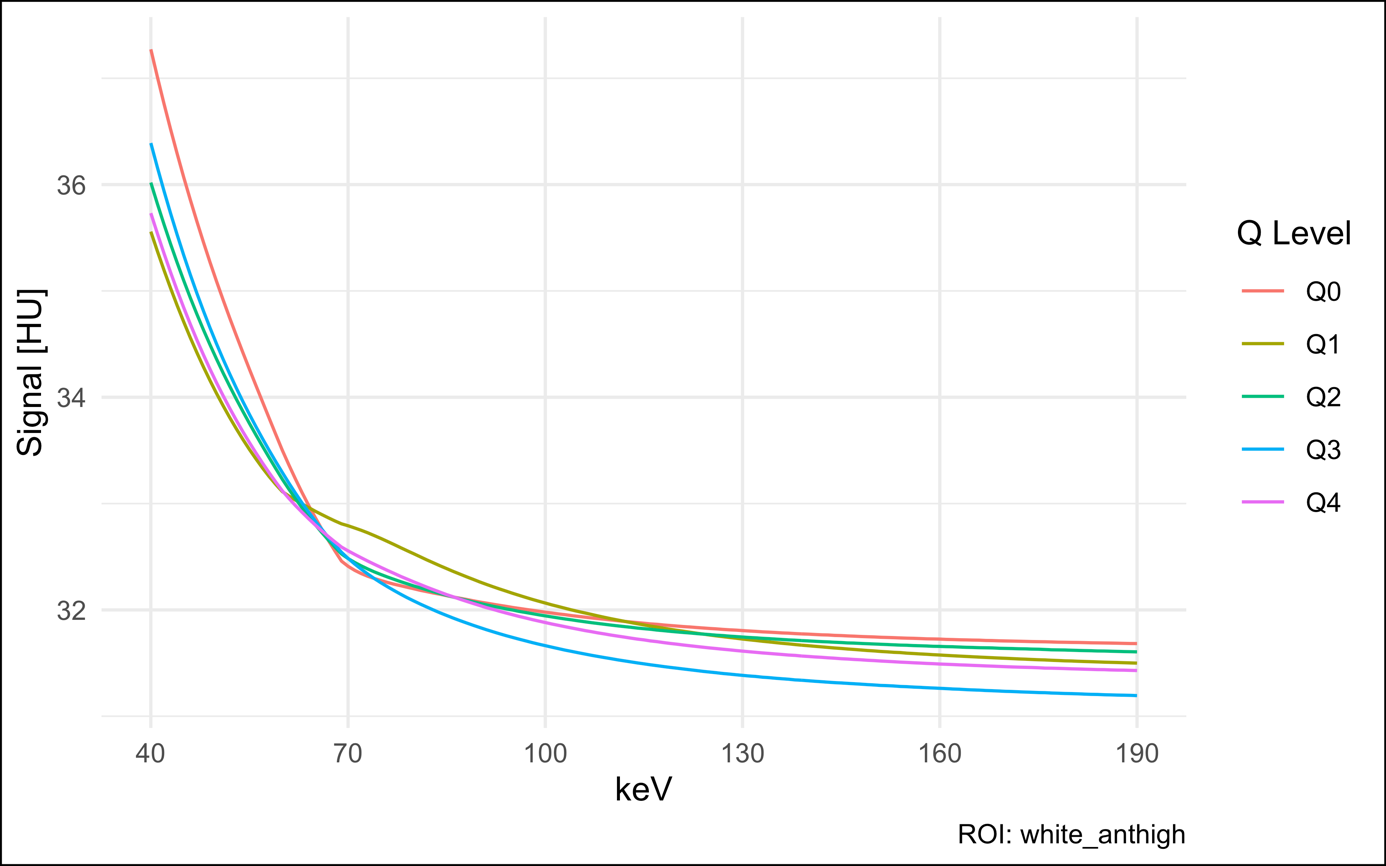


There is a significant difference between the signal characteristics of the individual virtual monoenergetic reconstructions (Friedman test corrected p < 0.0001) in ROI (11). The maximum white matter signal in the anterior internal capsule is found in the 40 keV VMI and averages 35.73 ± 5.03 HU in Q4 exemplarily. The differences between the individual Q levels are minimal and do not seem relevant. In post hoc testing, a significant difference in comparison to the next keV level of 41 keV is found (corrected p < 0.0001).

Selected keV levels with all Q levels and their post hoc tests can be reviewed in the supplemental material (Excel file “PCCT_CCT_Analysis.xlsx”, sheet “analysis_signal”).

## ROI (12): Gray Matter in the Inferior Caput Nuclei Caudati


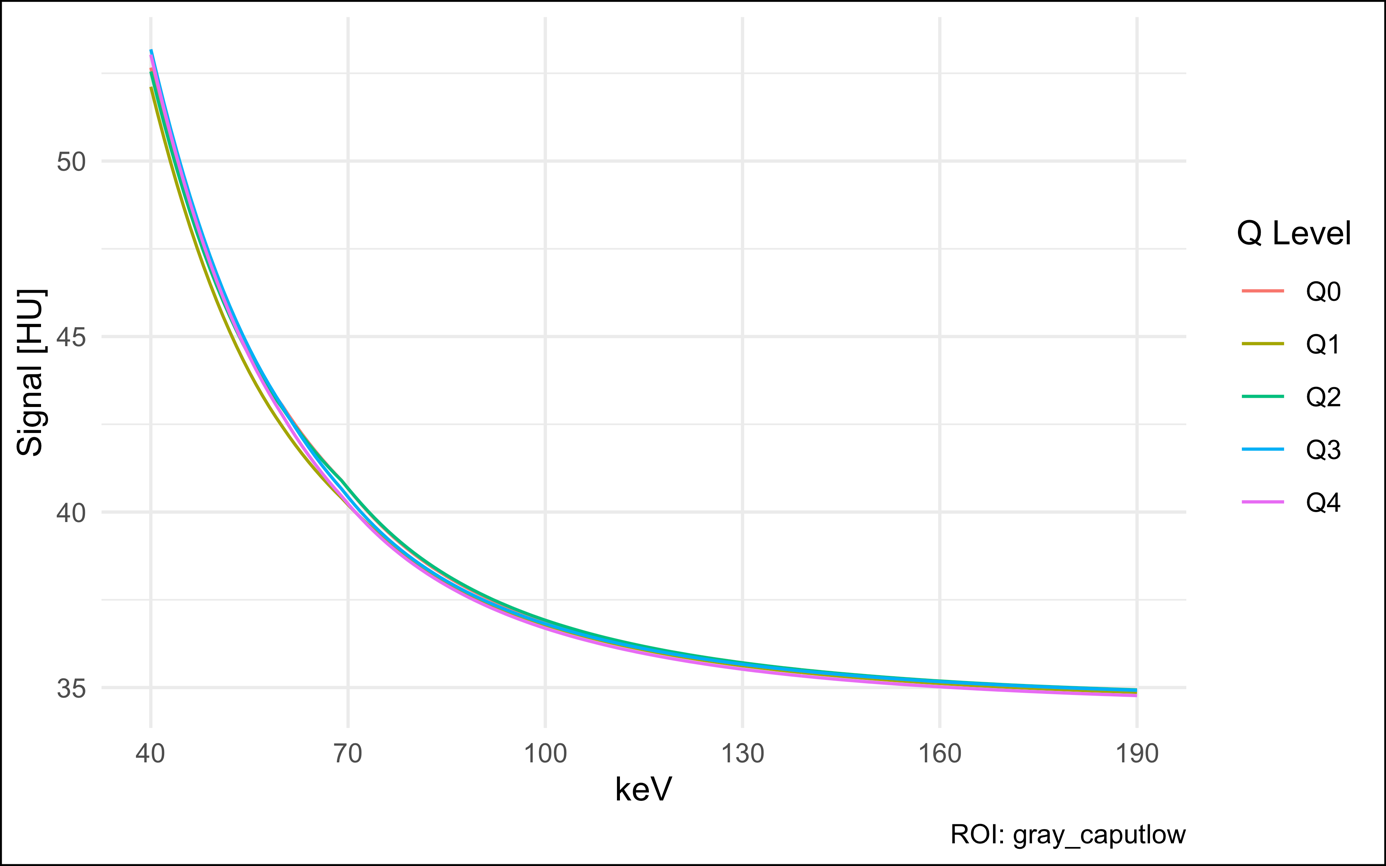


There is a significant difference between the signal characteristics of the individual virtual monoenergetic reconstructions (Friedman test corrected p < 0.0001) in ROI (12). The maximum gray matter signal in the inferior caput nuclei caudati is found in the 40 keV VMI and averages 53.03 ± 4.35 HU in Q4 exemplarily. The differences between the individual Q levels are minimal and do not seem relevant. In post hoc testing, a significant difference in comparison to the next keV level of 41 keV is found (corrected p < 0.0001).

Selected keV levels with all Q levels and their post hoc tests can be reviewed in the supplemental material (Excel file “PCCT_CCT_Analysis.xlsx”, sheet “analysis_signal”).

## ROI (13): White Matter adjacent to ROI (12) in the Anterior Callosum


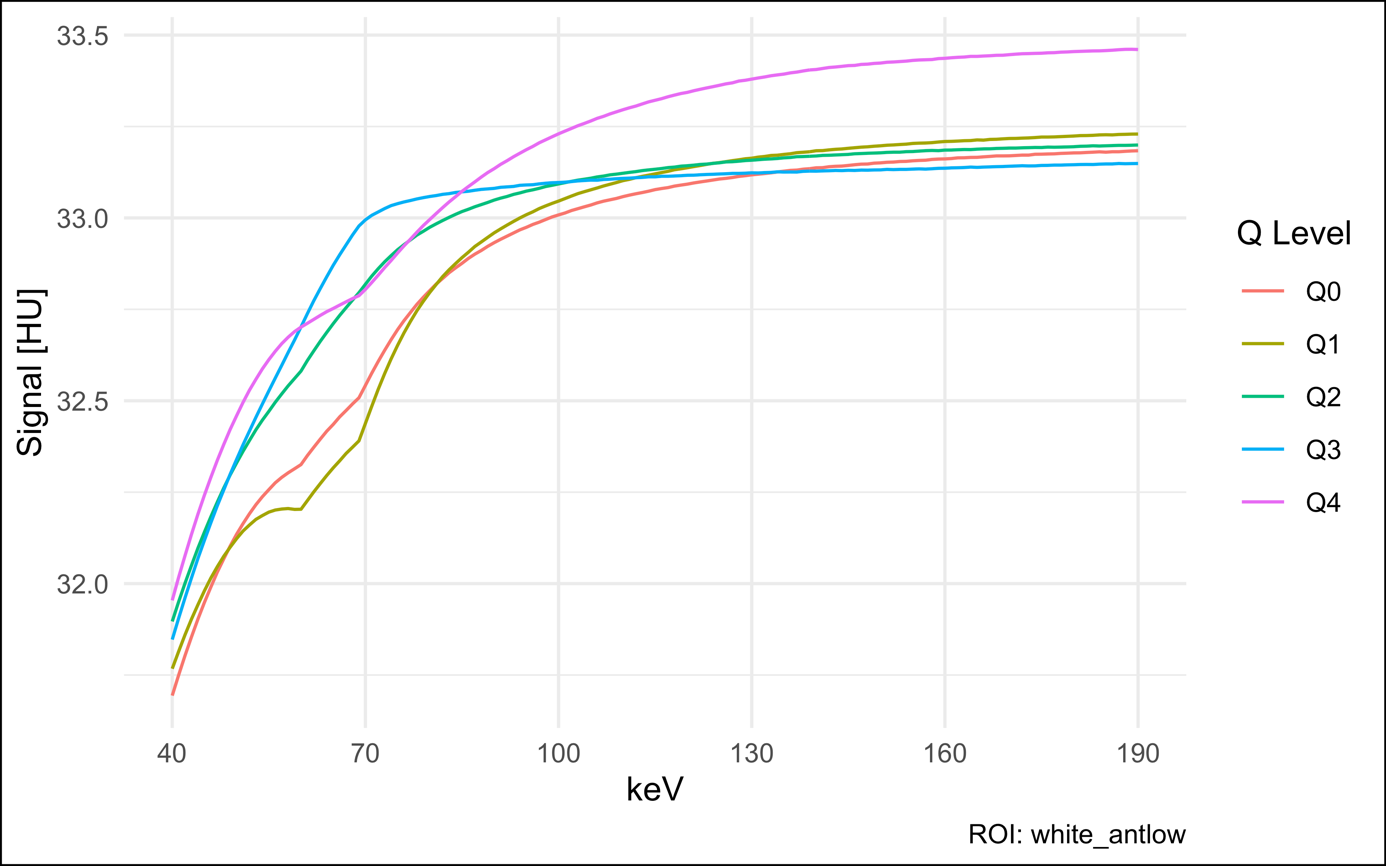


There is no significant difference between the signal characteristics of the individual virtual monoenergetic reconstructions (Friedman test corrected p > 0.05) in ROI (13) in Q levels 0 to 4. In Q level 4 however the very small differences reach significance due to low variance. The maximum white matter signal in the anterior callosum is found at 189 keV with 33.46 ± 5.08 HU, the post hoc tests show no significant differences to the adjacent low keV VMI.

Selected keV levels with all Q levels and their post hoc tests can be reviewed in the supplemental material (Excel file “PCCT_CCT_Analysis.xlsx”, sheet “analysis_signal”).

## ROI (14): Gray Matter in the Posterior Thalamus


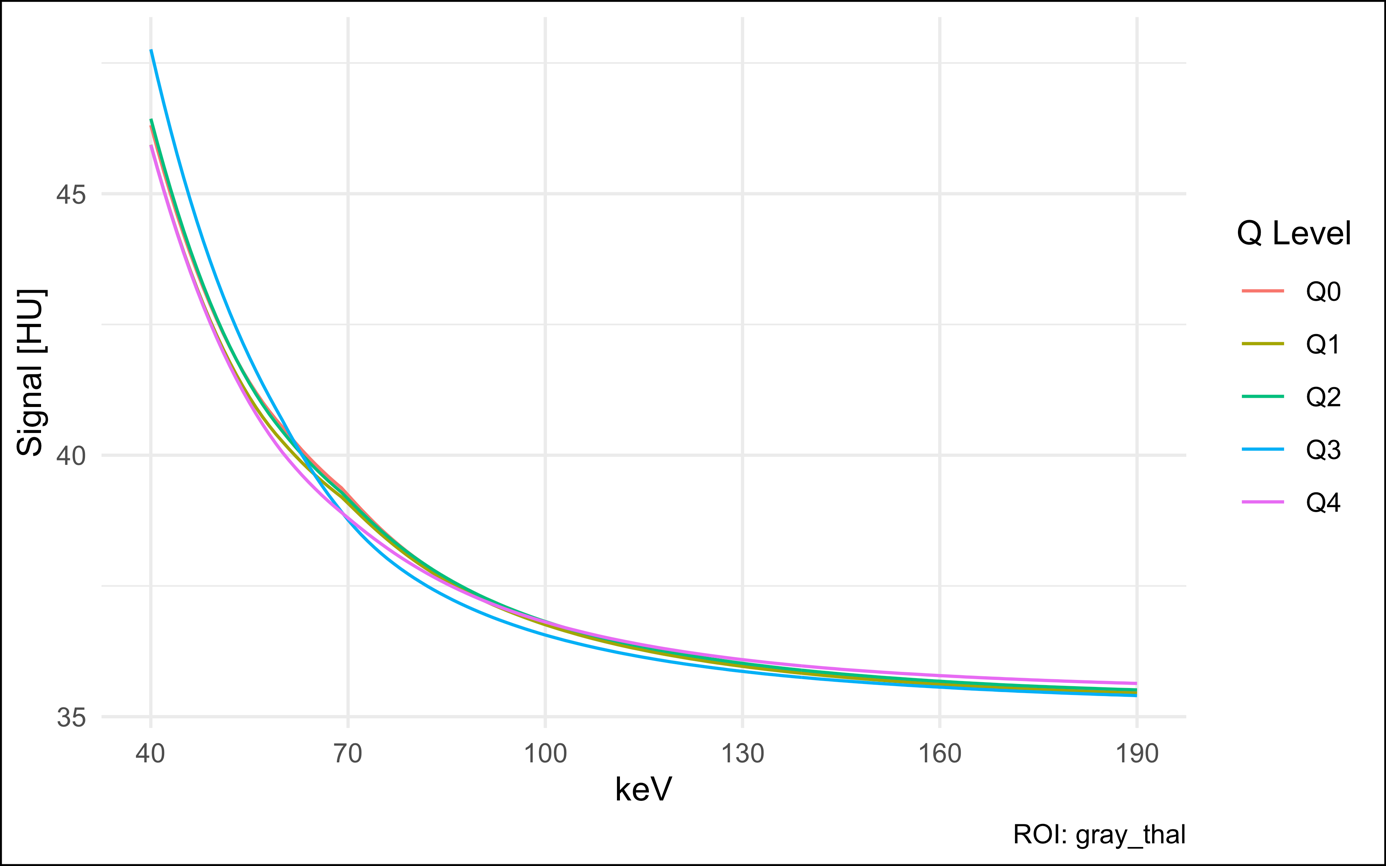


There is a significant difference between the signal characteristics of the individual virtual monoenergetic reconstructions (Friedman test corrected p < 0.0001) in ROI (14). The maximum gray matter signal in the posterior thalamus is found in the 40 keV VMI and averages 45.93 ± 3.95 HU in Q4 exemplarily. The differences between the individual Q levels are minimal and do not seem relevant. In post hoc testing, a significant difference in comparison to the next keV level of 41 keV is found (corrected p < 0.0001).

Selected keV levels with all Q levels and their post hoc tests can be reviewed in the supplemental material (Excel file “PCCT_CCT_Analysis.xlsx”, sheet “analysis_signal”).

## ROI (15): White Matter in the Posterior Internal Capsule


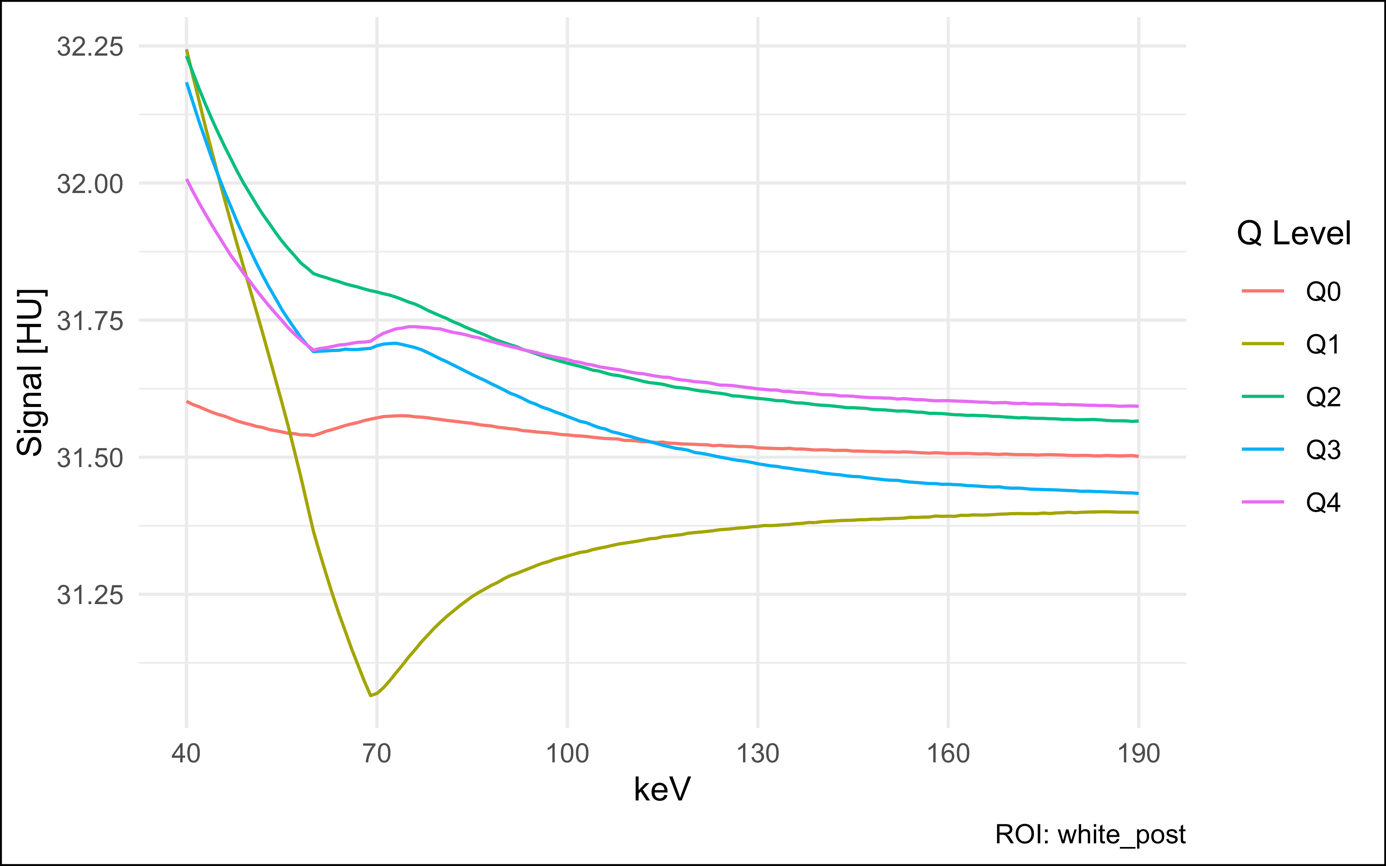


There is no significant difference between the signal characteristics of the individual virtual monoenergetic reconstructions (Friedman test corrected p > 0.05) in ROI (15) in Q levels 0 to 4. The graph shows a trend in terms of very small differences.

## ROI (16): White Matter in the Pons between the Petrous Bones


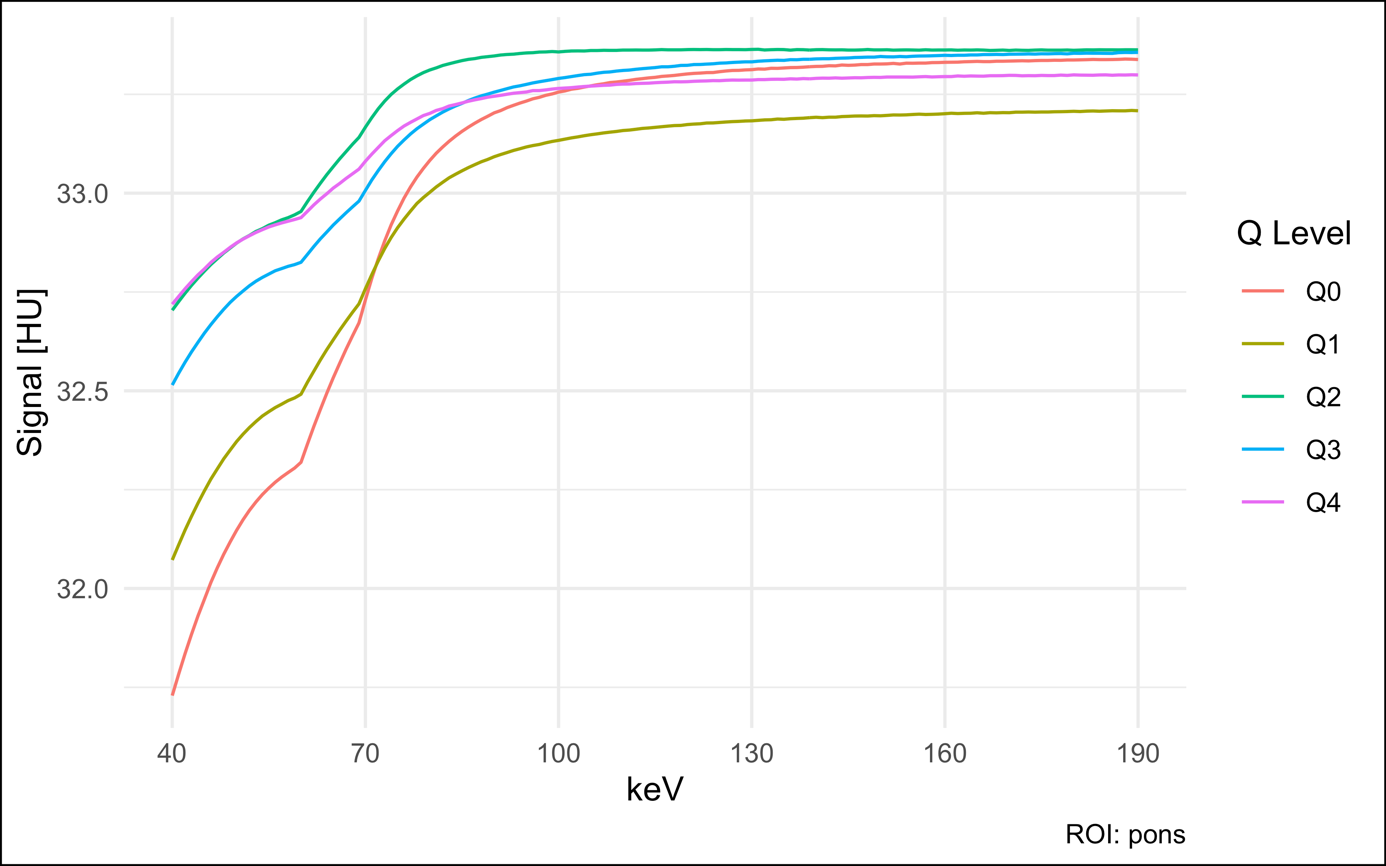


There is also no significant difference between the signal characteristics of the individual virtual monoenergetic reconstructions (Friedman test corrected p > 0.05) in ROI (16) in Q levels 0 to 4. The graph shows a trend in terms of very small differences.

Generally ROI (16) was designed exclusively for the analysis of the noise (as the “posterior fossa artifact index”), the evaluation of the signal is therefore only possible to a very limited extent.

# Results: Signal and Calvaria

## Signal of Cortical and Deep Gray and White Matter

When looking at the signal behavior of the ROIs with gray matter, it is noticeable that the ROIs with deep gray matter are relatively similar. The difference in signal between VMI with low and high keV is smallest in the thalamus - this ROI is paramedian and thus farthest from the cranial dome in the direction of the X-ray beam path and in the axial image reconstruction. The different distance to the bony skull base, however, does not seem to have any influence on the signal behavior, the gray matter in the superior and inferior caput nuclei caudati behaves more or less identically.


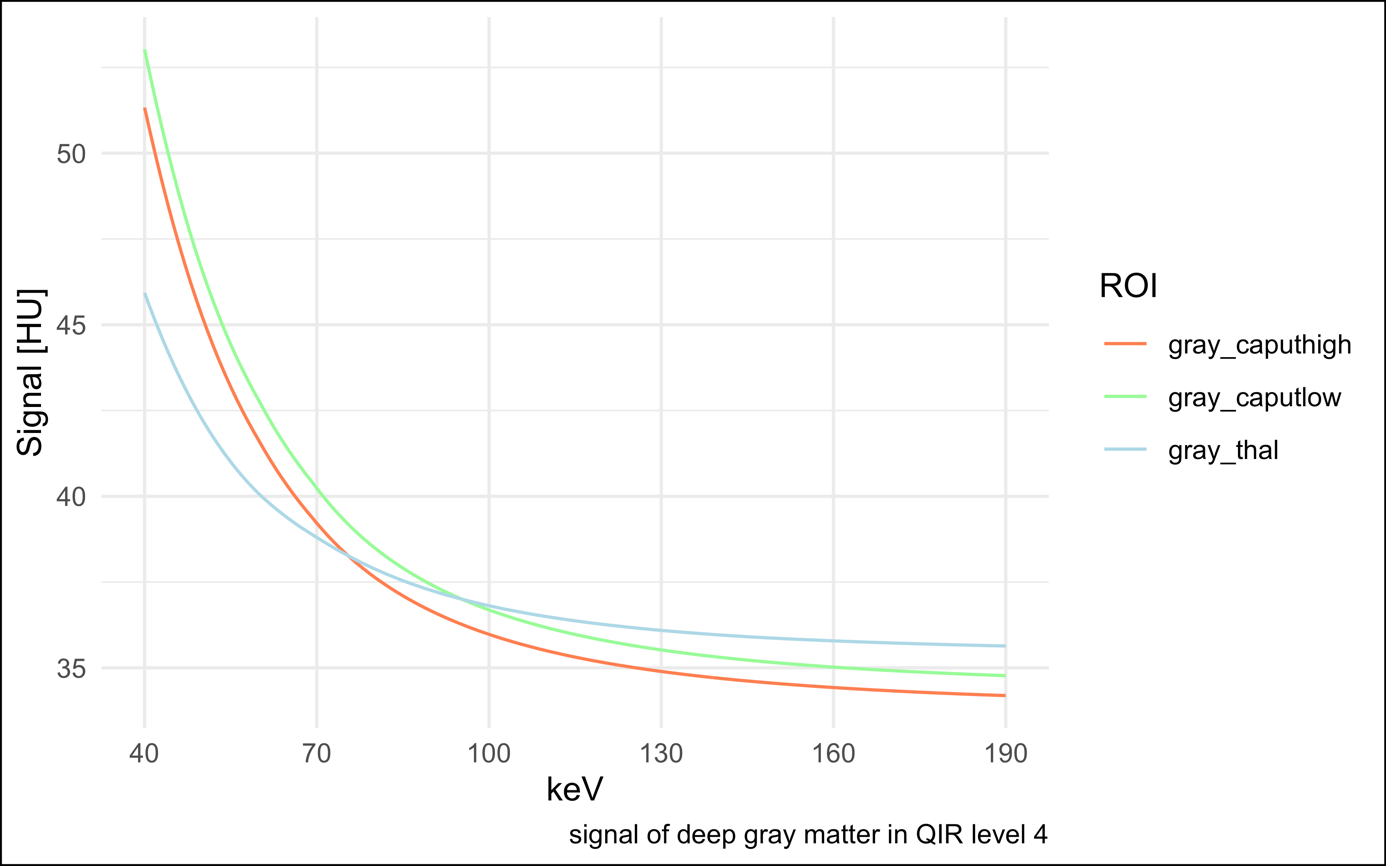


However, when looking at the ROIs in the cortex of the brain, it is noticeable that the ROIs closer to the cranial dome show a much higher signal in the low keV VMI. Only the ROI at 20 mm distance from the cranial dome shows an almost congruent signal behavior over the keV as the deep gray matter in the caudate head.


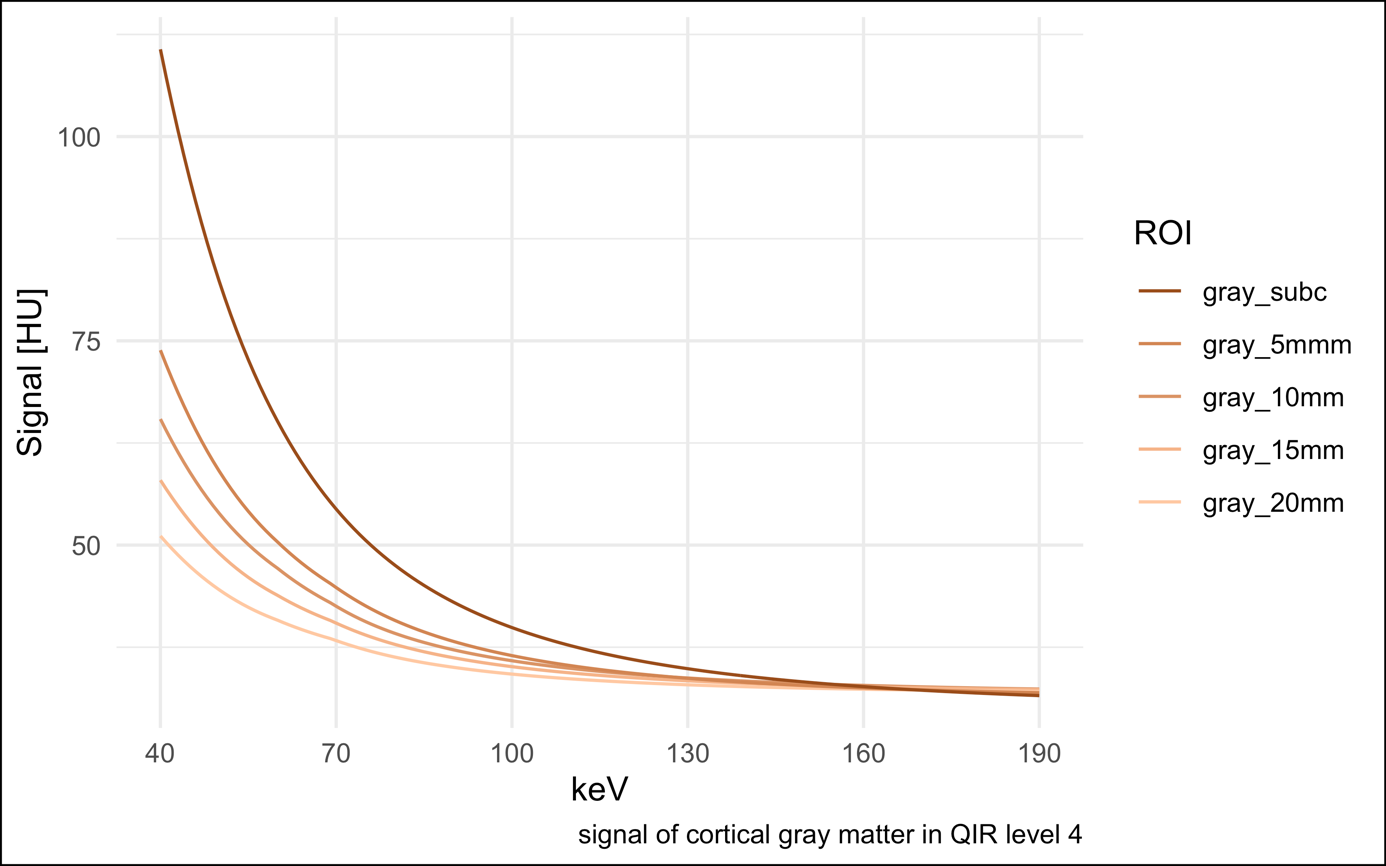


These clear differences can be graphically elaborated even better if not the absolute signal but the difference of the signal of the cortical ROI to the signal in a reference ROI with a larger distance to the cranial dome - here the gray matter in the superior caudate head was chosen.

In the following, the differences in the signal of the cortical ROIs to the reference ROI in the superior caudate head in all Q levels are shown. Once again, it becomes clear that there is no relevant difference between the Q levels.

| 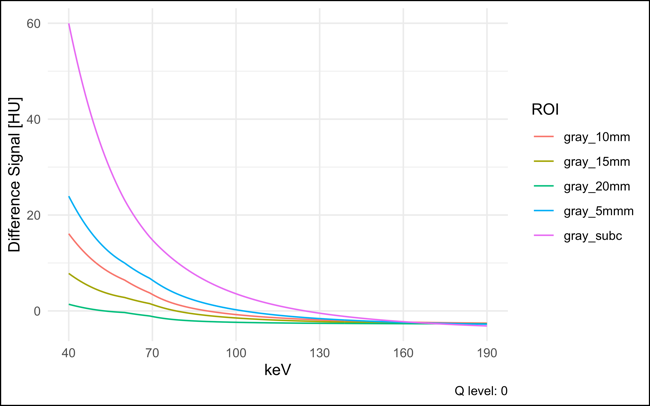 | 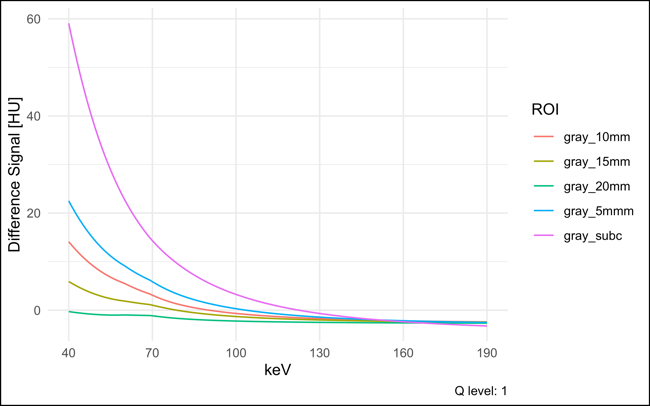 |
| --- | --- |
| 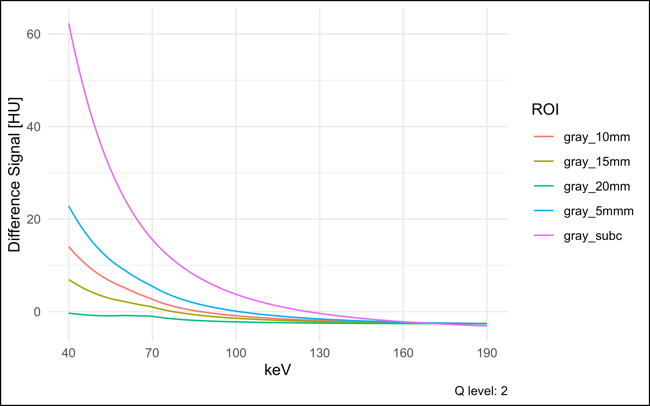 | 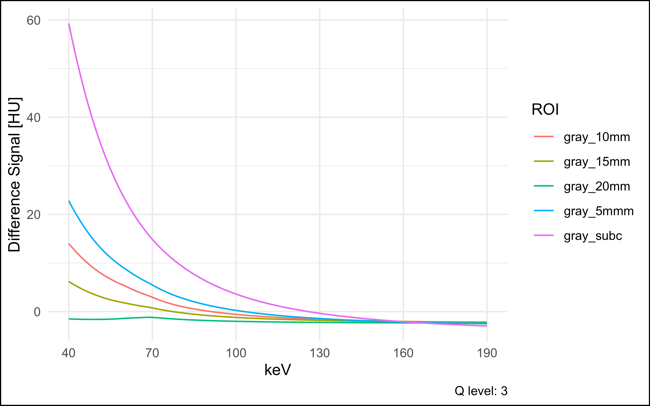 |
| 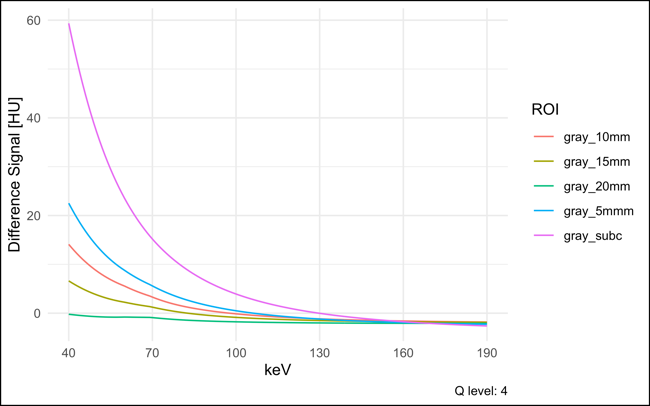 |  |

In the following, the fourth iterative level (Q level 4) is always considered for simplification. The signal behavior of the white matter adjacent to the gray matter is comparable, with the white matter having up to even more than 130 keV lower signal than the adjacent gray matter. Also in the white matter in the signal higher, the closer the ROI is to the cranial dome.


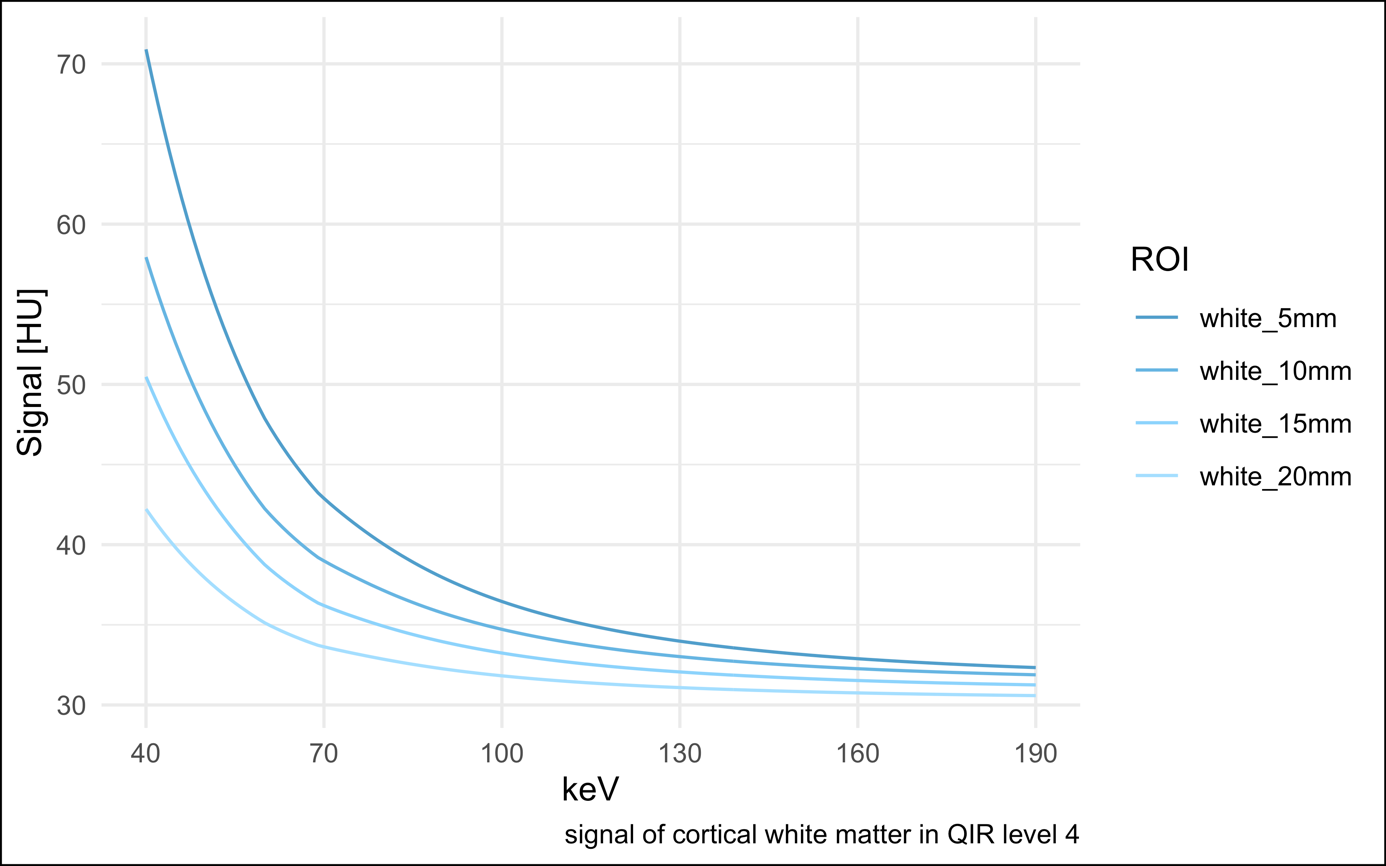


Thus, it happens that the white matter at a distance of 5 mm from the cranial dome has a higher signal in the low keV than the gray matter at 10 mm from the dome. In relation to the gray matter, the signal of the white matter first falls faster with increasing keV and then stagnates at low values in the higher keV.


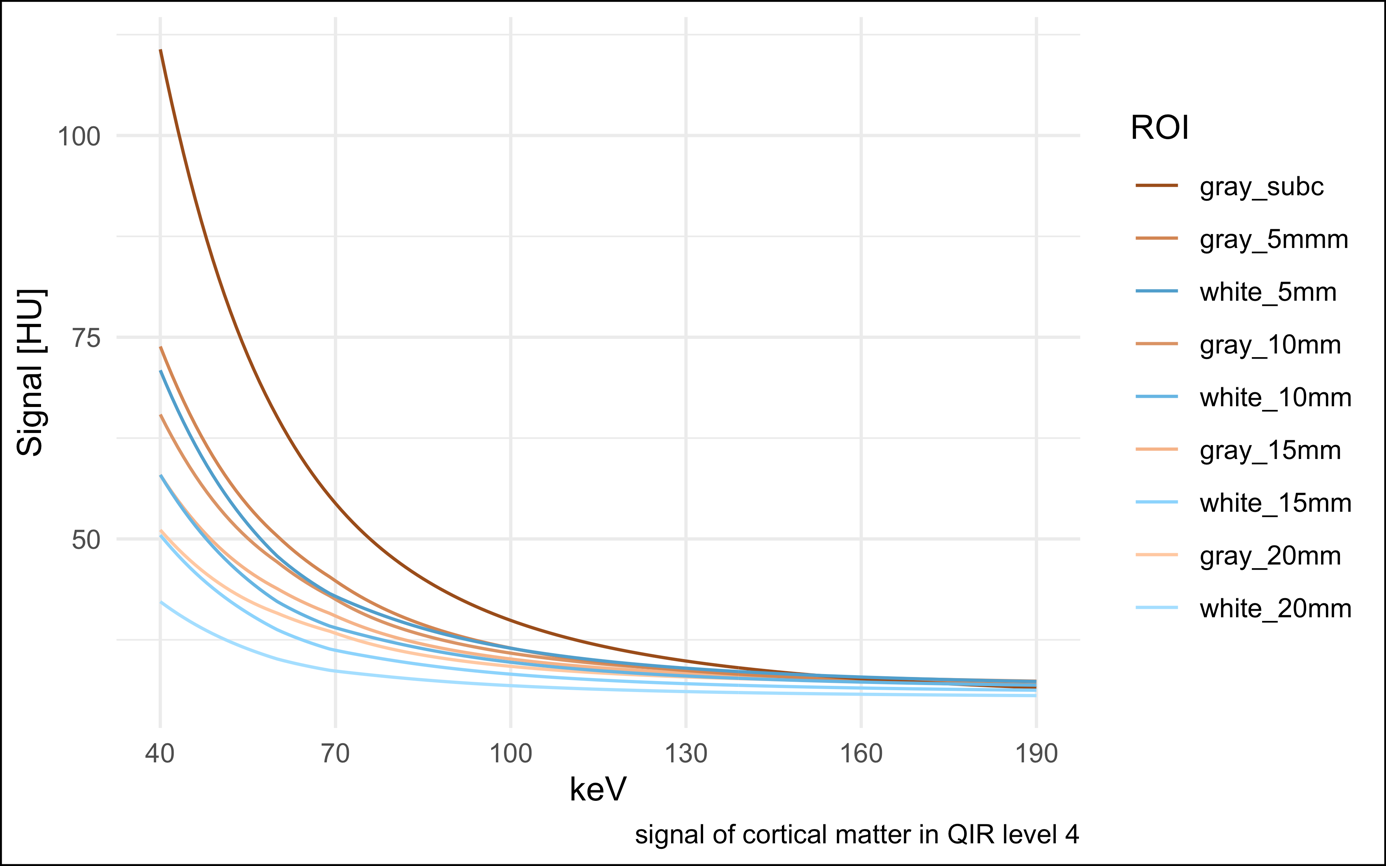


A different signal behavior is present in the white matter adjacent to the deep gray matter. The trend of the white matter signal in the anterior internal capsule is similar to the cortical ROIs - there were significant differences over the keV (see above). The white matter signal in the posterior internal capsule and adjacent to the internal caudate head - measured in the anterior callosum - is almost constant over the different keV, the trends shown are not significant (see above).


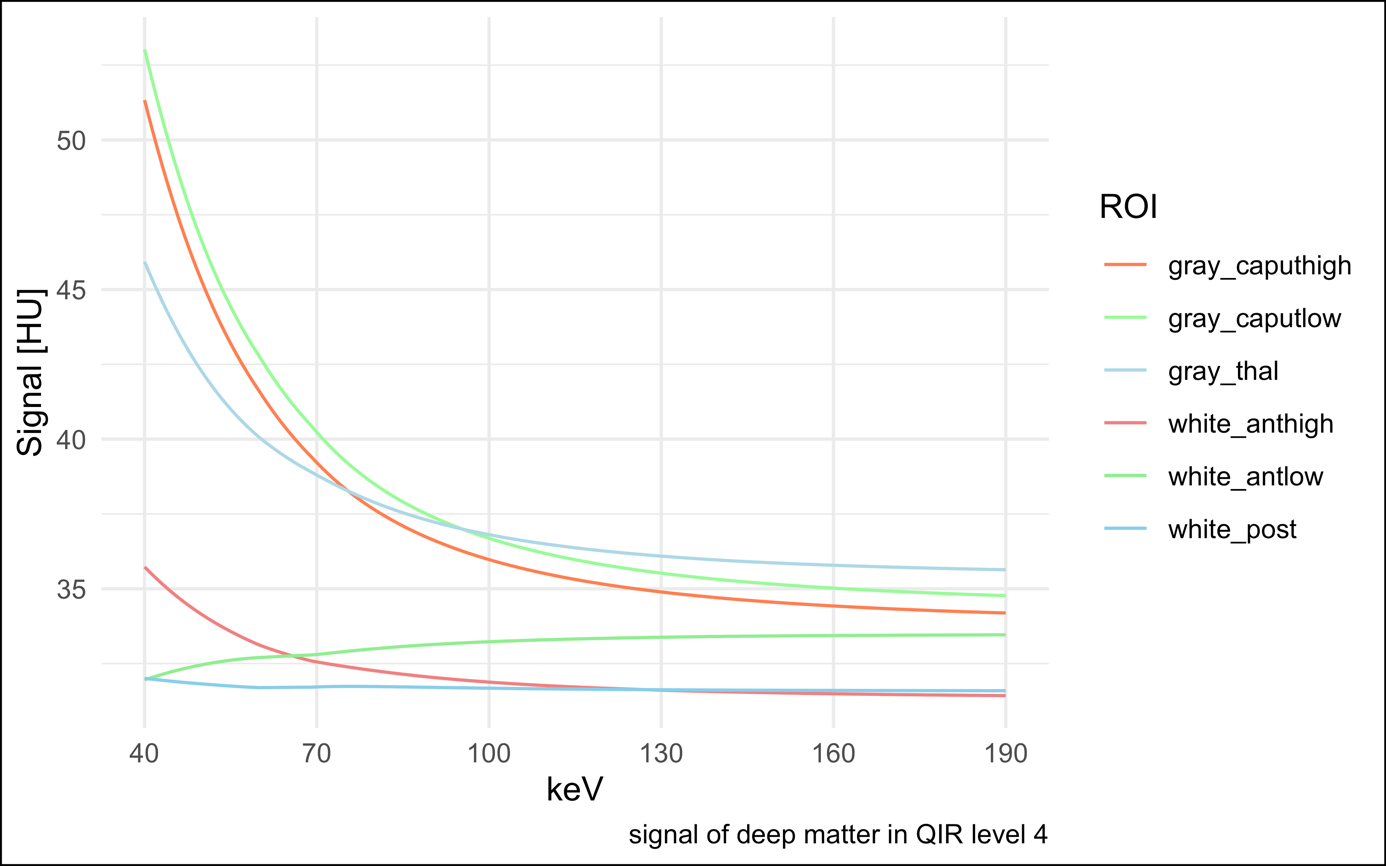


## Signal Differences: Correlation to Calvaria Thickness

It stands to reason that in addition to the distance to the cranial dome, the thickness of the latter also plays a role in the signal increase. At the position of the cortical ROIs, the thickness of the cranial dome was also measured as the diameter perpendicular to the tangent at the position of the cortical ROIs (see above).

This signal increase is most pronounced close to the cranial dome and in the low keV. Therefore, for plotting and calculating a correlation, the signal in the gray and white matter in the 40 keV in the ROI 5 mm below the cranial calvaria was chosen. The gray matter immediately below the calvaria was intentionally not chosen because of a slightly different distance of the brain matter from the calvaria of the skull due to a different brain volume reduction in older patients. The ROI 5 mm below the cranial dome is therefore more reliable in terms of distance. Since the signal does not differ relevantly in the different Q levels, the data of Q4 were used as an example.


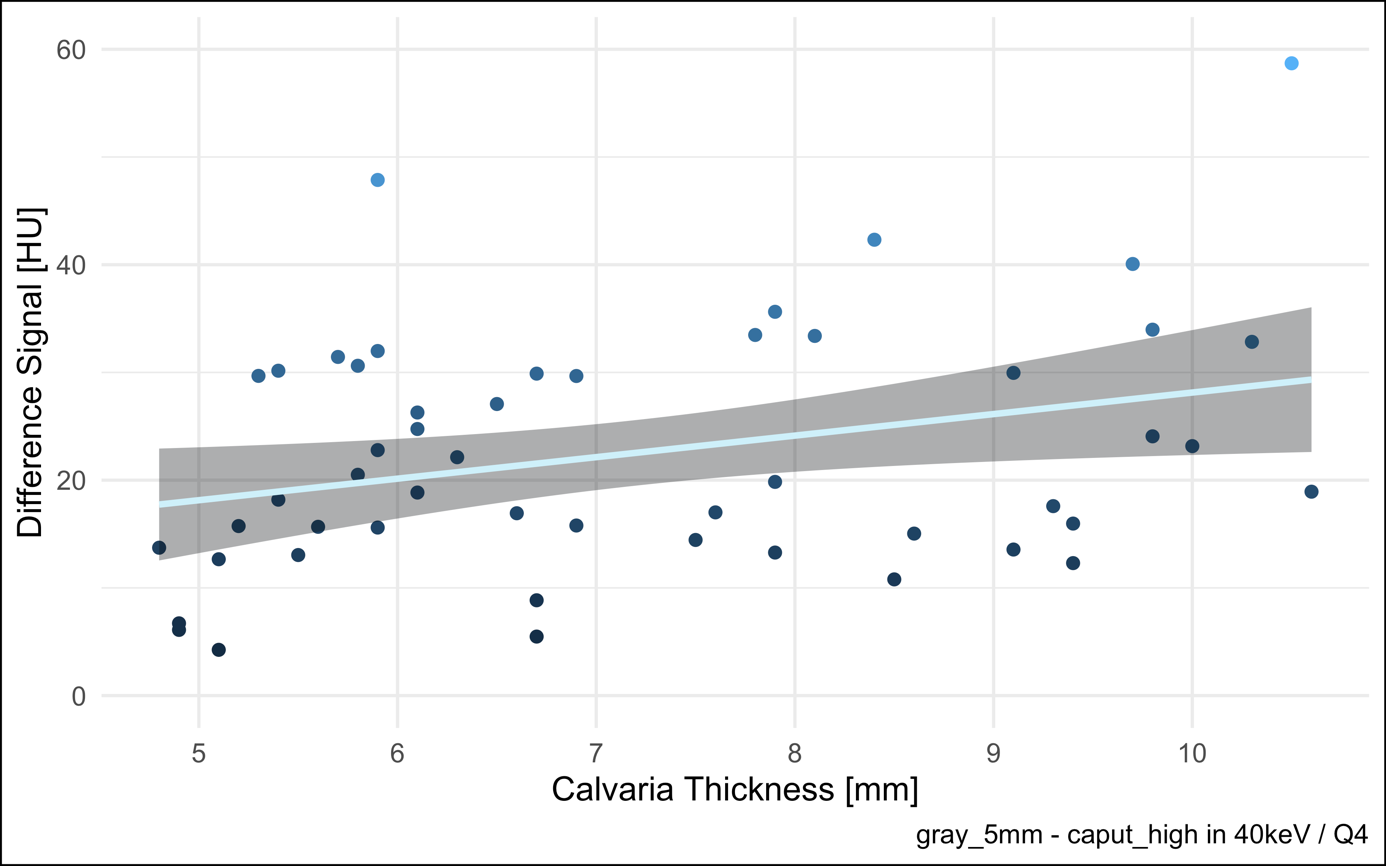


In the linear model, there is a very weak (adjusted r^2^ = 0.078) but significant (F-statistic: 5.221 on 1 and 49 df, p-value: 0.02669) relationship between the calvarial thickness and the difference in signal between the ROI in the gray matter 5 mm below the calvaria and the reference ROI in the superior caudate head. According to this model, with each millimeter of calotte thickness, this difference would increase by 2 HU (slope of linear model is 1.9991).

## Signal Differences: Calvaria and Causality

The correlation between the signal increase under the cranial dome and the thickness of the dome, although weak, makes a significant contribution. In order to be able to prove the assumed causality, another series of measurements was used.

The signal behavior of gray matter of the cortex should be determined on the one hand in the presence of the cranial calvaria - without doubt the usual situation - and on the other hand without the cranial calvaria. In the period available for retrospective inclusion of patients of this study, two patients with craniectomy shortly before reimplantation were also found. In the unenhanced CCT of these patients, ROIs in cortical substance could be measured both near and far from the cranial calvaria. In the CCT of these two patients, 10 ROIs each with a size of 4 mm were placed on the side with cranial dome at approximately 5 to 10 mm from it; on the side of craniectomy, 10 ROIs were placed at 5 to 10 mm from the dura. In both hemispheres, of course, only brain parenchyma was included that was judged to be intact by CT morphology.


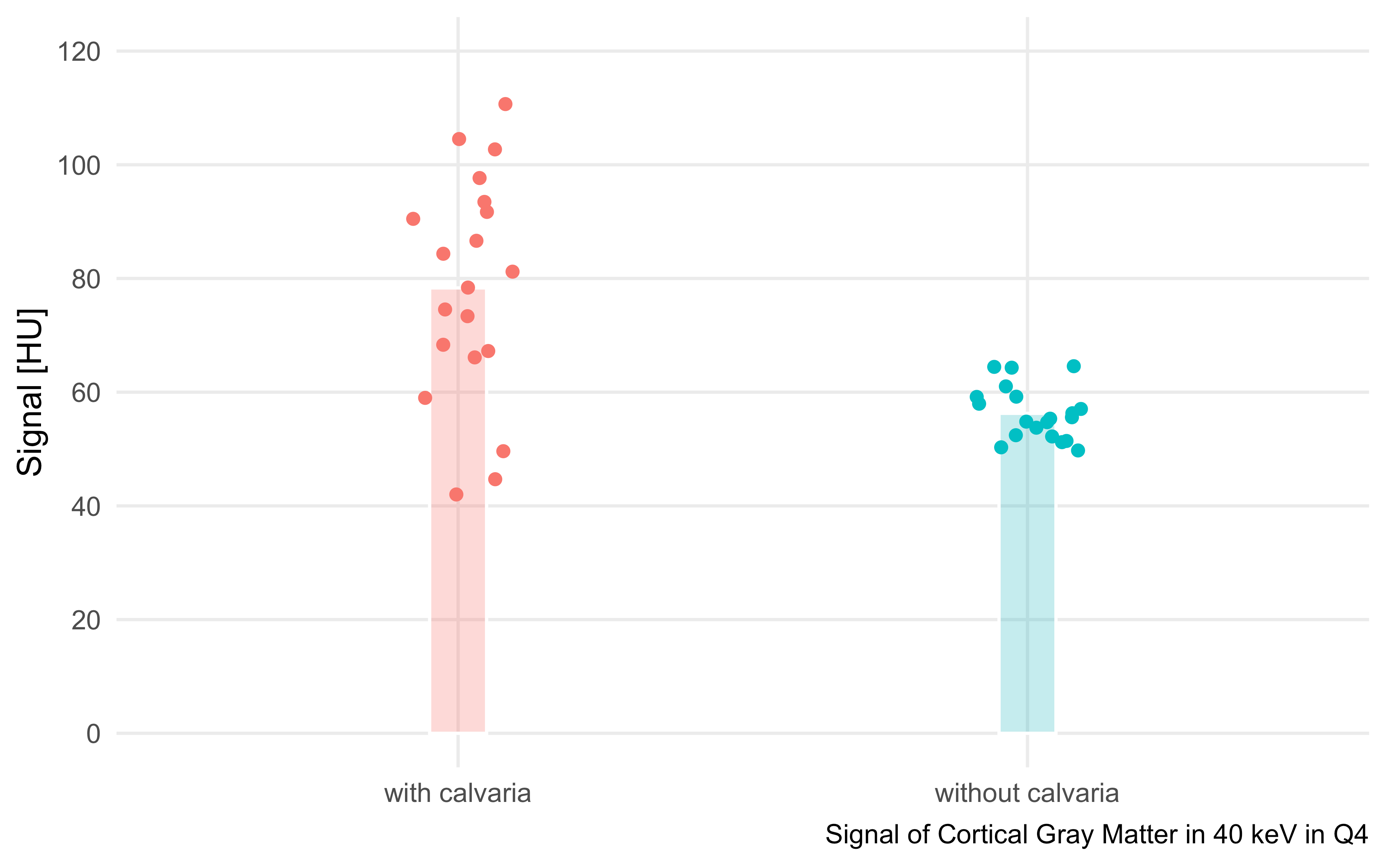


Both the signal values below the calvaria and on the side without the calvaria are normally distributed (Shapiro-Wilk p > 0.05). The gray matter signal below the calvaria averages 78.34 ± 19.78 HU, on the side without calvaria only 56.28 ± 4.67 HU. The difference between the variances is significant (Levene's test p = 0.0000249). The difference between the means is also significant (Welch's test p = 0.00008328). Hereby, the causal relationship of cranial calvaria and increased signal of the brain substance below the dome is proven.

# Results: First Approach to Noise

## ROI (1): Gray Matter below the Calvaria


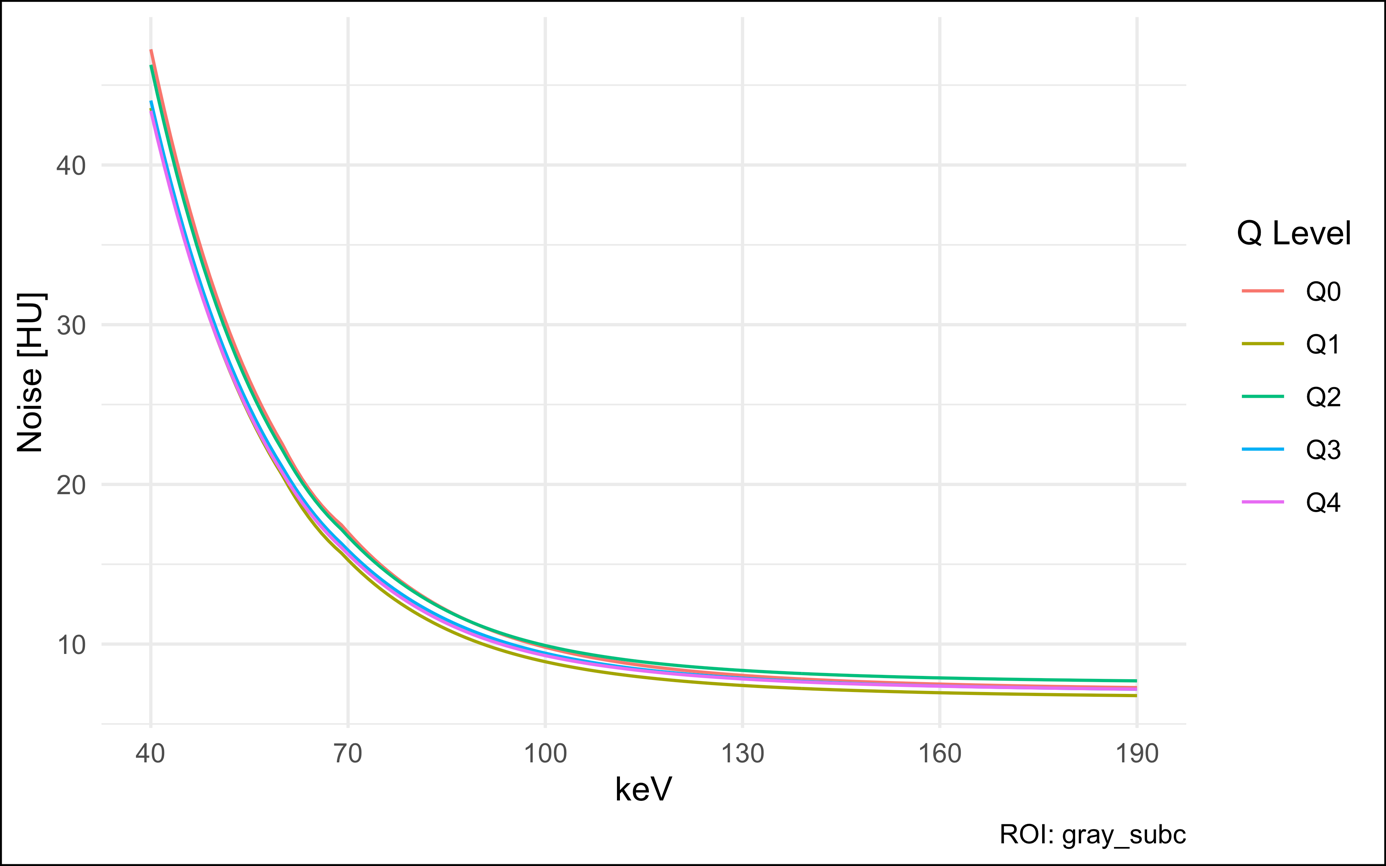


There is a significant difference between the noise characteristics of the individual virtual monoenergetic reconstructions (Friedman test corrected p < 0.0001) in ROI (1). Interestingly, there is just a relatively small difference between the Q levels. In this respect, the ROI directly below the cranial calvaria is clearly different from all other ROIs. The minimum noise without iterative reconstruction is found in the 190 keV VMI with 7.28 ± 40.18 HU. Also the huge standard deviation of 40.18 HU is a peculiarity of this ROI (and is probably due to the influential artifacts of the cranial calvaria).

In post hoc testing, a significant difference is found only to the keV in the lower range up to 110 keV (110 keV: corrected p = 0.0155).

Selected keV levels with all Q levels and their post hoc tests can be reviewed in the supplemental material (Excel file “PCCT_CCT_Analysis.xlsx”, sheet “analysis_noise”).

## ROI (2): Cortical Gray Matter 5 mm below the Calvaria


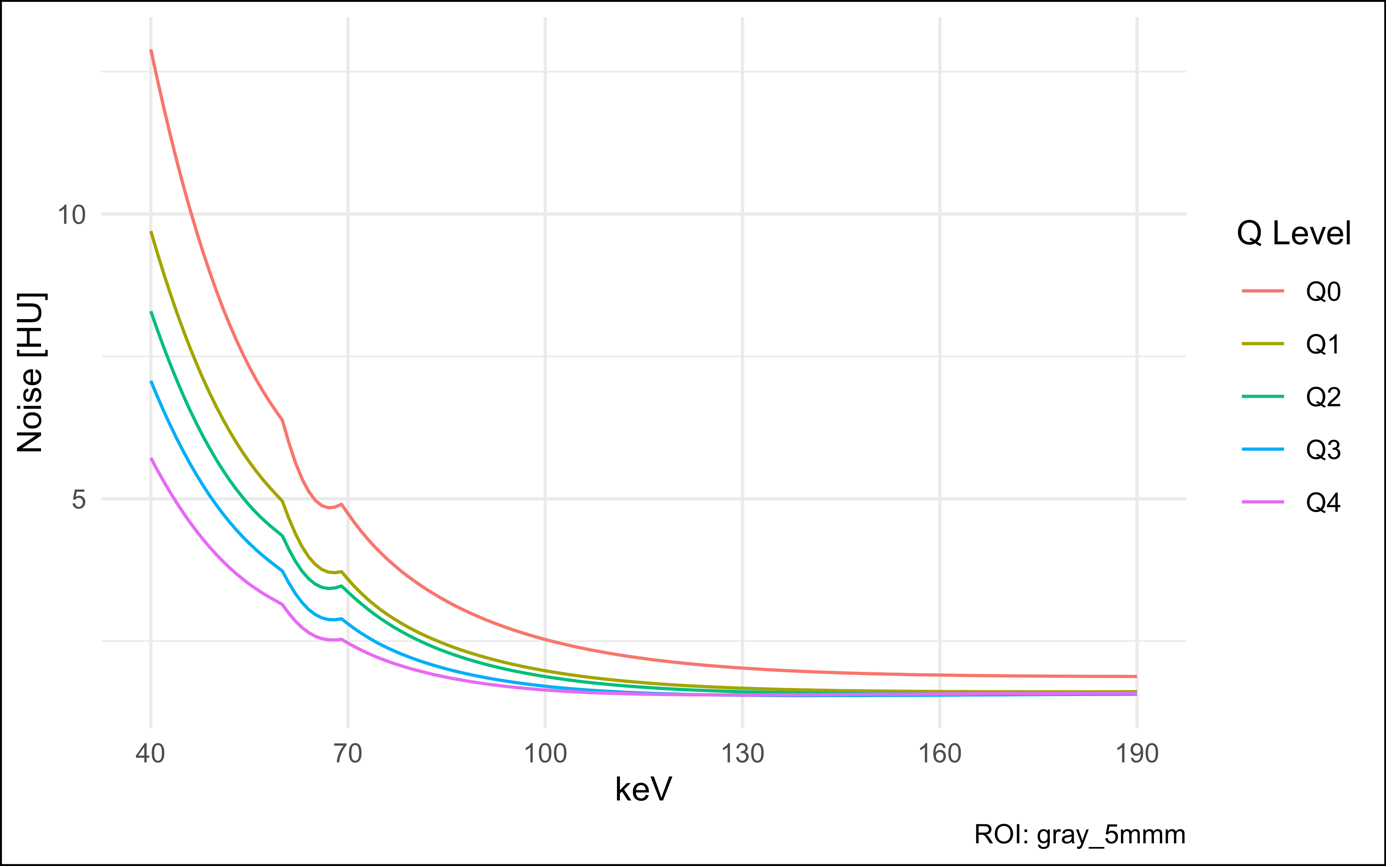


There is a significant difference between the noise characteristics of the individual virtual monoenergetic reconstructions (Friedman test corrected p < 0.0001) in ROI (2). As expected, the iterative reconstruction leads to a reduction of noise with each level. Between 60 and 70 keV, there is a focally emphasized lowering of the noise in each iterative level.

In **Q0** the minimum noise is found in the 131 keV VMI with 1.55 ± 1.47 HU. In post hoc testing, a significant difference is found only to the keV in the lower range up to 170 keV (170 keV: corrected p = 0.00809).

In **Q4** the minimum noise is found in the 190 keV VMI with 1.88 ± 2.54 HU. In post hoc testing, a significant difference is found only to the keV in the lower range up to 100 keV (100 keV: corrected p = 0.03681).

Selected keV levels with all Q levels and their post hoc tests can be reviewed in the supplemental material (Excel file “PCCT_CCT_Analysis.xlsx”, sheet “analysis_noise”).

## ROI (3): Cortical White Matter 5 mm below the Calvaria


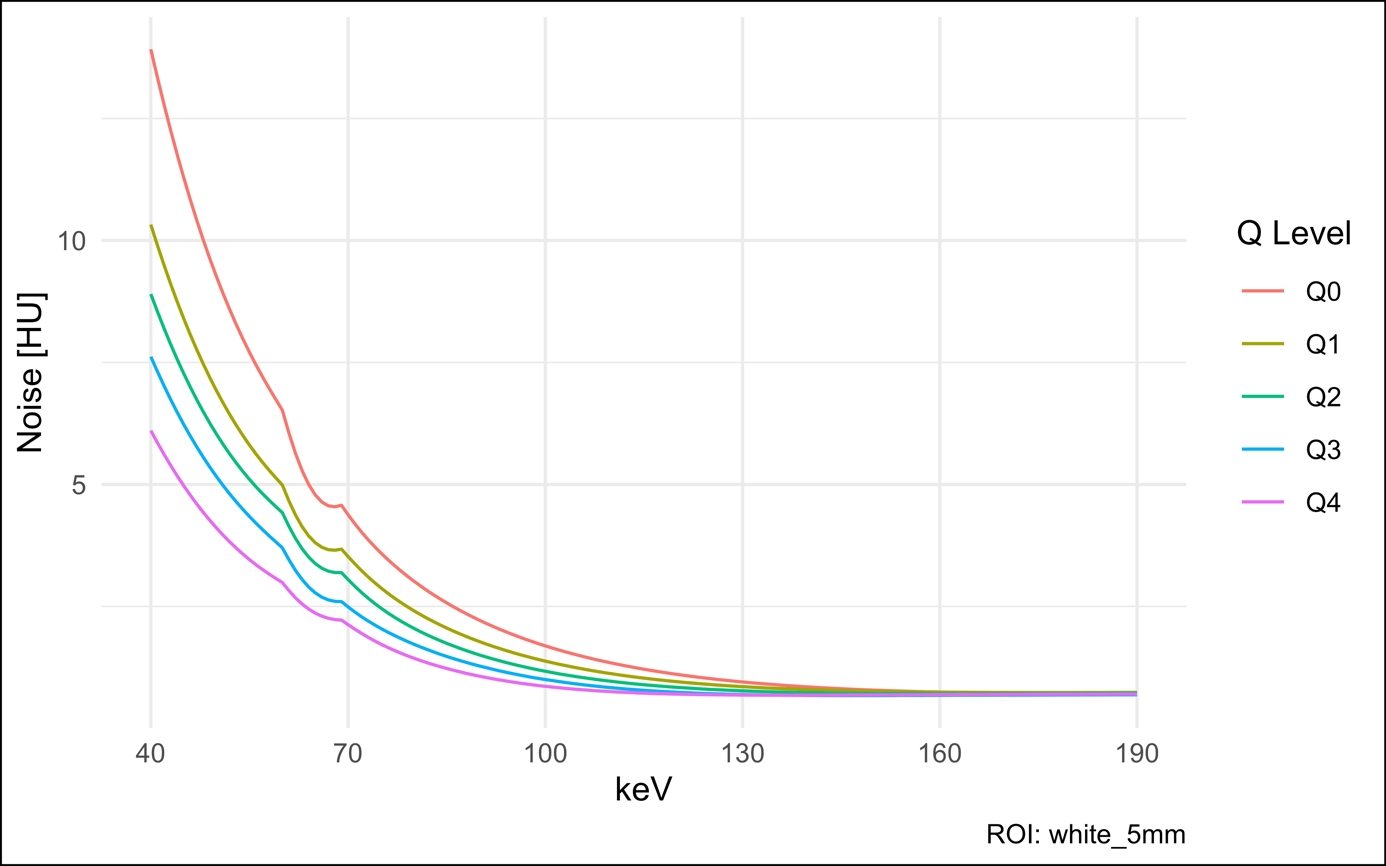


There is a significant difference between the noise characteristics of the individual virtual monoenergetic reconstructions (Friedman test corrected p < 0.0001) in ROI (3). As expected, the iterative reconstruction leads to a reduction of noise with each level. Between 60 and 70 keV, there is a focally emphasized lowering of the noise in each iterative level.

In **Q0** the minimum noise is found in the 190 keV VMI with 0.7 ± 3.74 HU. In post hoc testing, a significant difference is found only to the keV in the lower range up to 160 keV (160 keV: corrected p = 0.00047).

In **Q4** the minimum noise is found in the 136 keV VMI with 0.68 ± 0.48 HU. In post hoc testing, a significant difference is found only to the keV in the lower range up to 110 keV (110 keV: corrected p = 0.00824).

Selected keV levels with all Q levels and their post hoc tests can be reviewed in the supplemental material (Excel file “PCCT_CCT_Analysis.xlsx”, sheet “analysis_noise”).

## ROI (4): Cortical Gray Matter 10 mm below the Calvaria


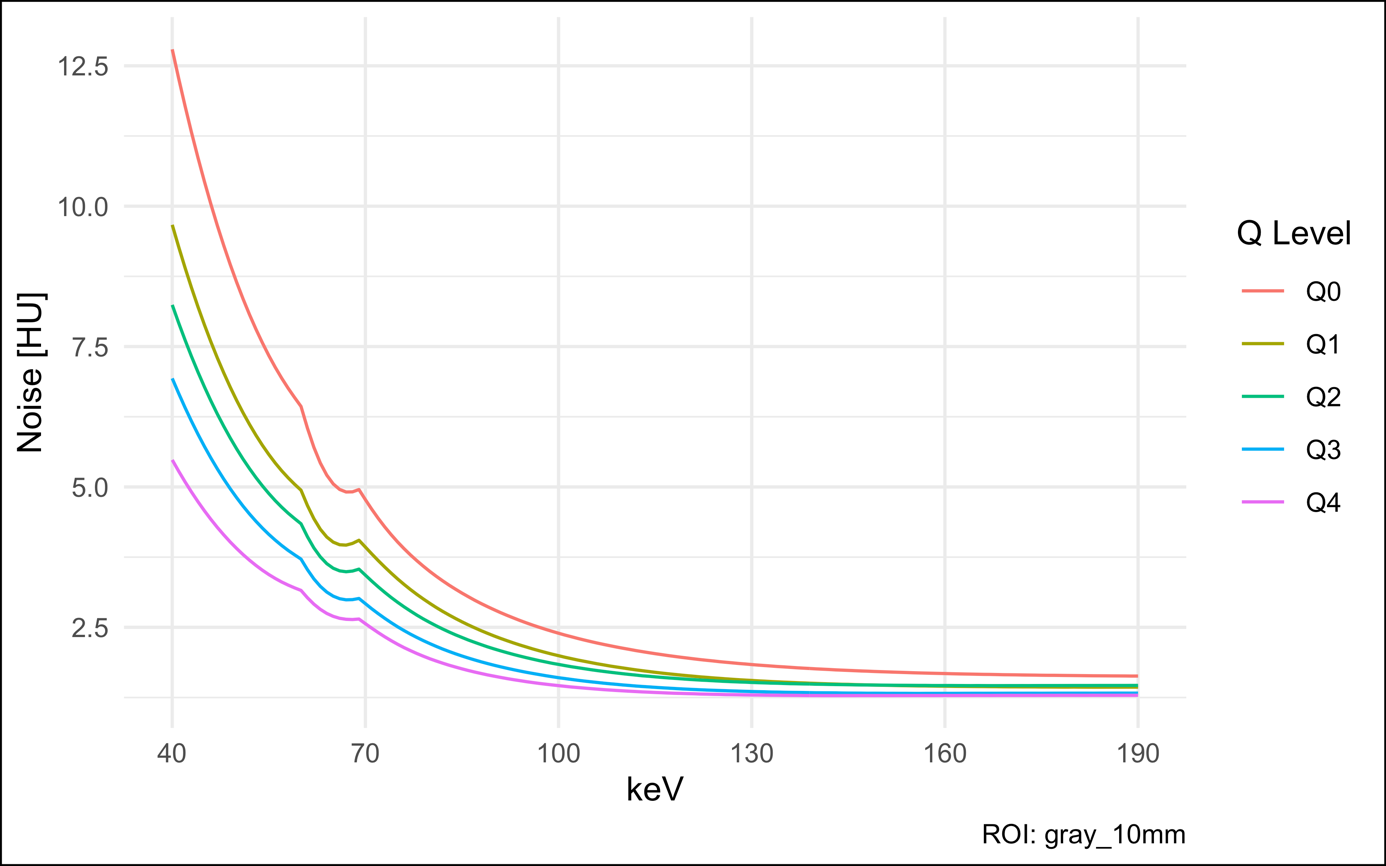


There is a significant difference between the noise characteristics of the individual virtual monoenergetic reconstructions (Friedman test corrected p < 0.0001) in ROI (4). As expected, the iterative reconstruction leads to a reduction of noise with each level. Between 60 and 70 keV, there is a focally emphasized lowering of the noise in each iterative level.

In **Q0** the minimum noise is found in the 189 keV VMI with 1.63 ± 1.43 HU. In post hoc testing, a significant difference is found only to the keV in the lower range up to 186 keV (160 keV: corrected p = 0.02074).

In **Q4** the minimum noise is found in the 142 keV VMI with 1.28 ± 1.14 HU. In post hoc testing, a significant difference is found only to the keV in the lower range up to 120 keV (120 keV: corrected p = 0.00118).

Selected keV levels with all Q levels and their post hoc tests can be reviewed in the supplemental material (Excel file “PCCT_CCT_Analysis.xlsx”, sheet “analysis_noise”).

## ROI (5): White Matter 10 mm below the Calvaria


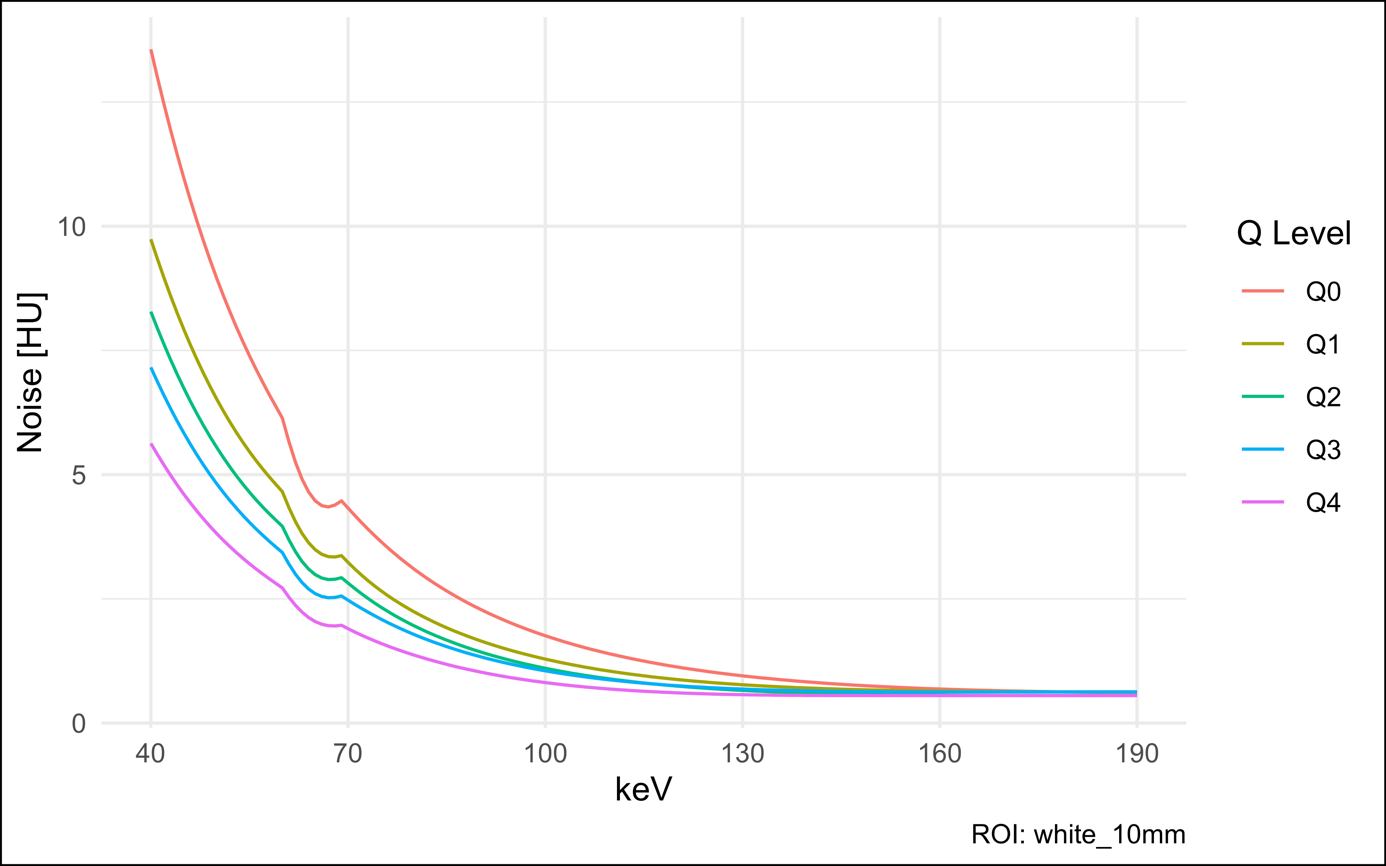


There is a significant difference between the noise characteristics of the individual virtual monoenergetic reconstructions (Friedman test corrected p < 0.0001) in ROI (5). As expected, the iterative reconstruction leads to a reduction of noise with each level. Between 60 and 70 keV, there is a focally emphasized lowering of the noise in each iterative level.

In **Q0** the minimum noise is found in the 190 keV VMI with 0.61 ± 2.48 HU. In post hoc testing, a significant difference is found only to the keV in the lower range up to 188 keV (188 keV: corrected p = 0.03876).

In **Q4** the minimum noise is found in the 163 keV VMI with 0.55 ± 0.27 HU. In post hoc testing, a significant difference is found only to the keV in the lower range up to 130 keV (130 keV: corrected p = 0.02512).

Selected keV levels with all Q levels and their post hoc tests can be reviewed in the supplemental material (Excel file “PCCT_CCT_Analysis.xlsx”, sheet “analysis_noise”).

## ROI (6): Cortical Gray Matter 15 mm below the Calvaria


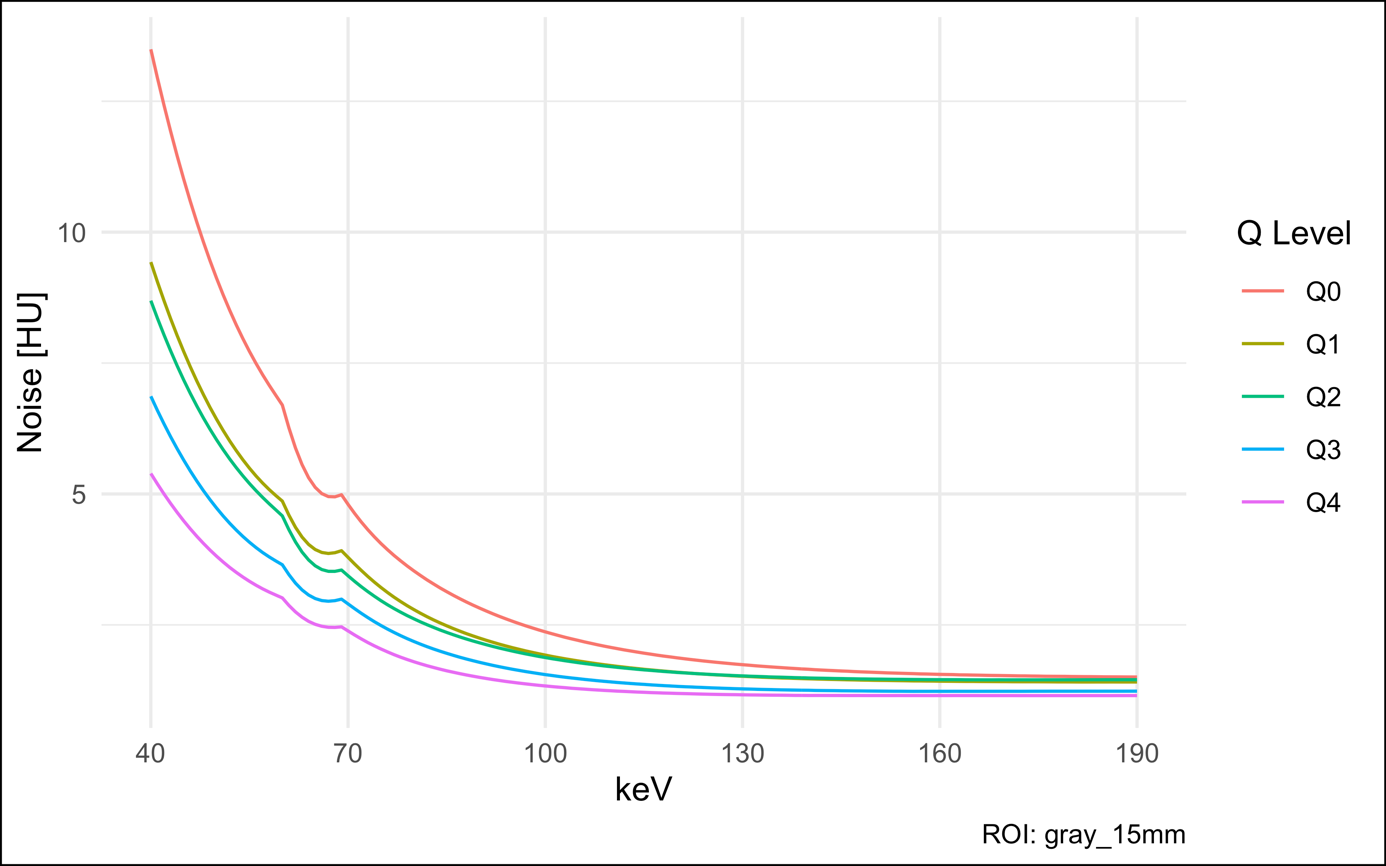


There is a significant difference between the noise characteristics of the individual virtual monoenergetic reconstructions (Friedman test corrected p < 0.0001) in ROI (6). As expected, the iterative reconstruction leads to a reduction of noise with each level. Between 60 and 70 keV, there is a focally emphasized lowering of the noise in each iterative level.

In **Q0** the minimum noise is found in the 190 keV VMI with 1.50 ± 3.06 HU. In post hoc testing, a significant difference is found only to the keV in the lower range up to 180 keV (180 keV: corrected p = 0.00699).

In **Q4** the minimum noise is found in the 172 keV VMI with 1.15 ± 1.01 HU. In post hoc testing, a significant difference is found only to the keV in the lower range up to 110 keV (110 keV: corrected p = 0.00181).

Selected keV levels with all Q levels and their post hoc tests can be reviewed in the supplemental material (Excel file “PCCT_CCT_Analysis.xlsx”, sheet “analysis_noise”).

## ROI (7): Cortical White Matter 15 mm below the Calvaria


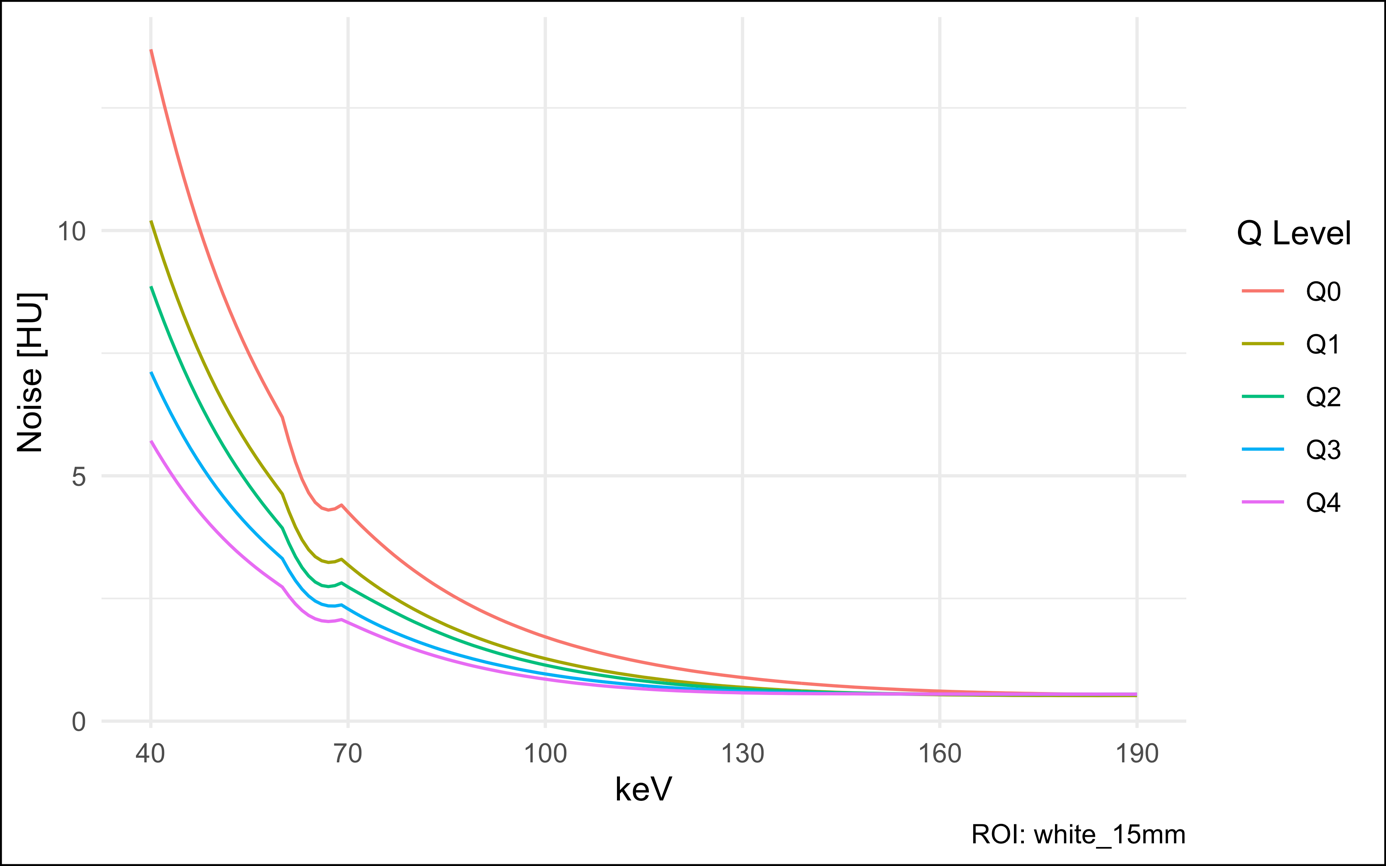


There is a significant difference between the noise characteristics of the individual virtual monoenergetic reconstructions (Friedman test corrected p < 0.0001) in ROI (7). As expected, the iterative reconstruction leads to a reduction of noise with each level. Between 60 and 70 keV, there is a focally emphasized lowering of the noise in each iterative level.

In **Q0** the minimum noise is found in the 190 keV VMI with 0.54 ± 3.08 HU. In post hoc testing, a significant difference is found only to the keV in the lower range up to 186 keV (186 keV: corrected p = 0.01665).

In **Q4** the minimum noise is found in the 189 keV VMI with 0.55 ± 0.26 HU. In post hoc testing, a significant difference is found only to the keV in the lower range up to 130 keV (130 keV: corrected p = 0.03212).

Selected keV levels with all Q levels and their post hoc tests can be reviewed in the supplemental material (Excel file “PCCT_CCT_Analysis.xlsx”, sheet “analysis_noise”).

## ROI (8): Cortical Gray Matter 20 mm below the Calvaria


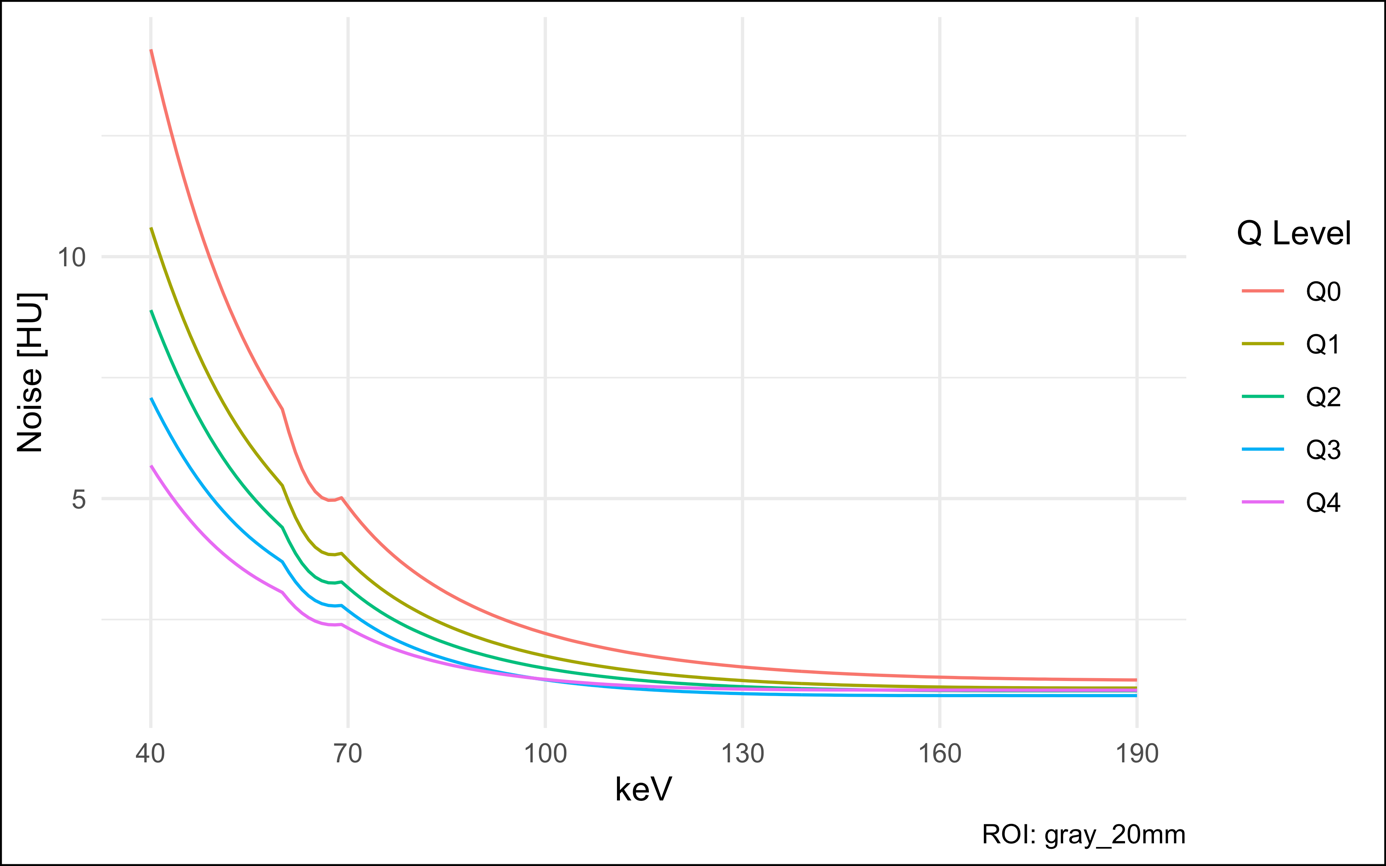


There is a significant difference between the noise characteristics of the individual virtual monoenergetic reconstructions (Friedman test corrected p < 0.0001) in ROI (8). As expected, the iterative reconstruction leads to a reduction of noise with each level. Between 60 and 70 keV, there is a focally emphasized lowering of the noise in each iterative level.

In **Q0** the minimum noise is found in the 190 keV VMI with 1.25 ± 3.07 HU. In post hoc testing, a significant difference is found only to the keV in the lower range up to 185 keV (185 keV: corrected p = 0.02496).

In **Q4** the minimum noise is found in the 189 keV VMI with 1.04 ± 0.93 HU. In post hoc testing, a significant difference is found only to the keV in the lower range up to 130 keV (130 keV: corrected p = 0.00748).

Selected keV levels with all Q levels and their post hoc tests can be reviewed in the supplemental material (Excel file “PCCT_CCT_Analysis.xlsx”, sheet “analysis_noise”).

## ROI (9): Cortical White Matter 20 mm below the Calvaria


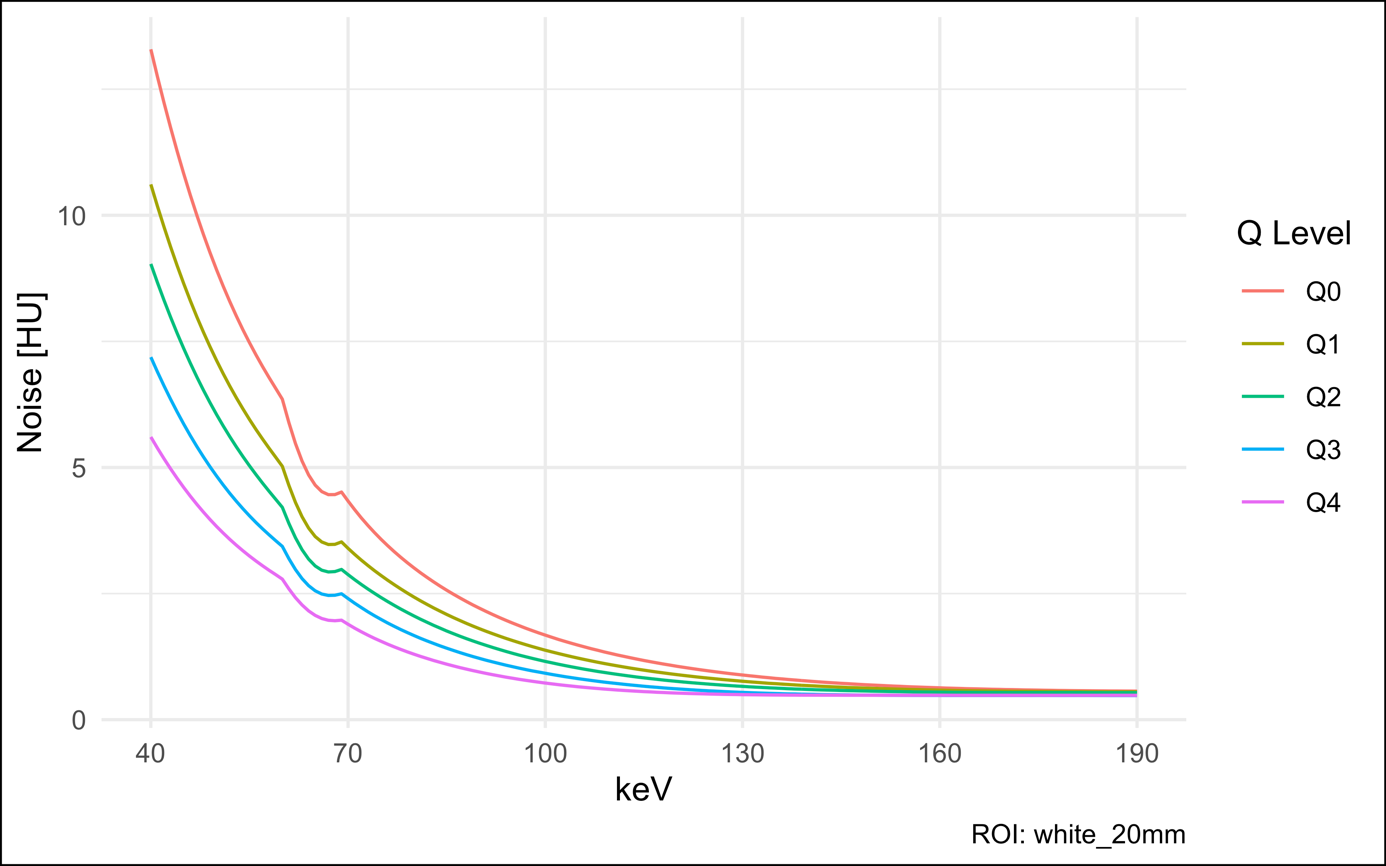


There is a significant difference between the noise characteristics of the individual virtual monoenergetic reconstructions (Friedman test corrected p < 0.0001) in ROI (9). As expected, the iterative reconstruction leads to a reduction of noise with each level. Between 60 and 70 keV, there is a focally emphasized lowering of the noise in each iterative level.

In **Q0** the minimum noise is found in the 190 keV VMI with 0.57 ± 2.86 HU. In post hoc testing, a significant difference is found only to the keV in the lower range up to 188 keV (188 keV: corrected p = 0.03676).

In **Q4** the minimum noise is found in the 177 keV VMI with 0.48 ± 0.15 HU. In post hoc testing, a significant difference is found only to the keV in the lower range up to 120 keV (120 keV: corrected p = 0.00741).

Selected keV levels with all Q levels and their post hoc tests can be reviewed in the supplemental material (Excel file “PCCT_CCT_Analysis.xlsx”, sheet “analysis_noise”).

## ROI (10): Gray Matter in the Superior Caput Nuclei Caudati


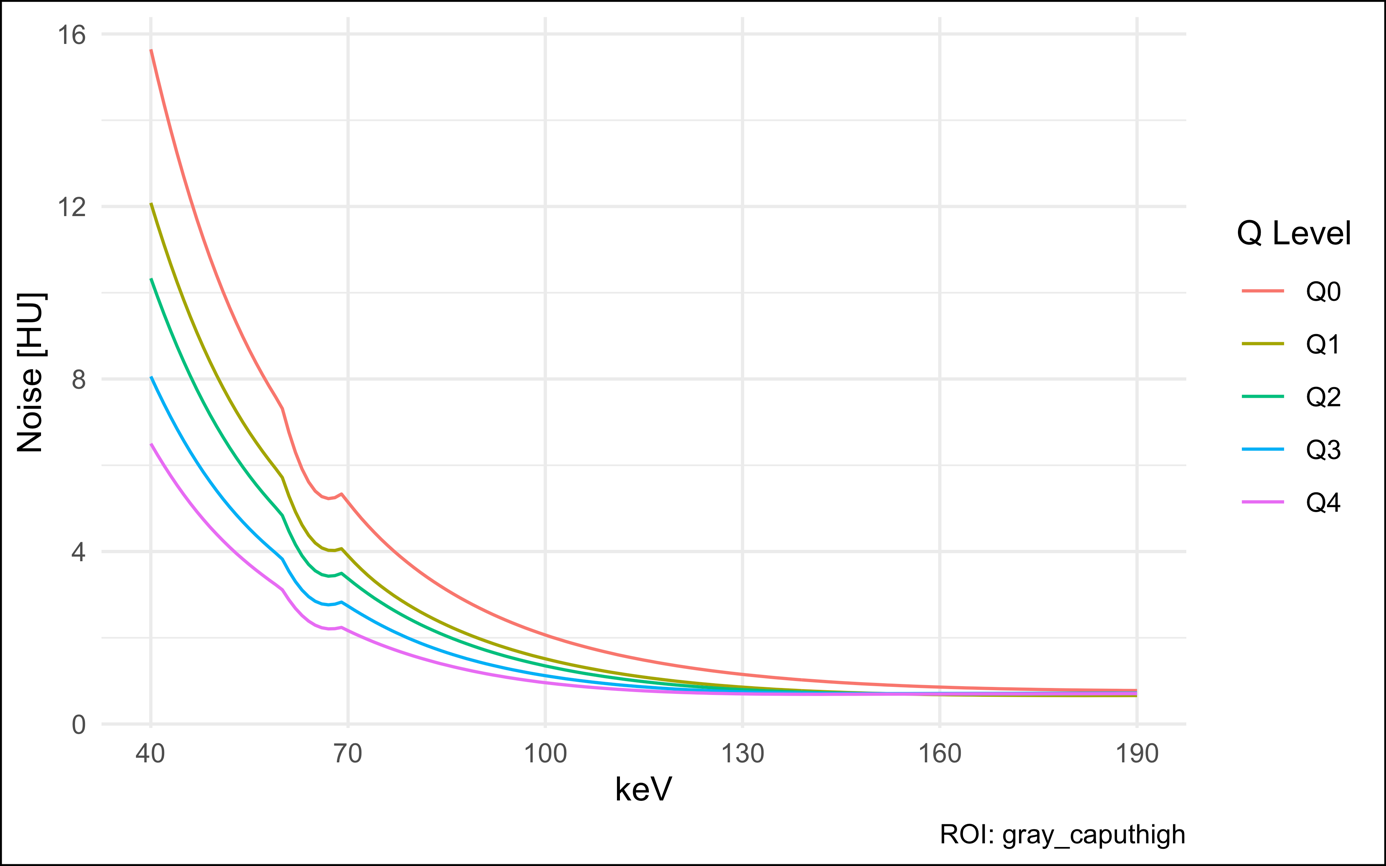


There is a significant difference between the noise characteristics of the individual virtual monoenergetic reconstructions (Friedman test corrected p < 0.0001) in ROI (10). As expected, the iterative reconstruction leads to a reduction of noise with each level. Between 60 and 70 keV, there is a focally emphasized lowering of the noise in each iterative level.

In **Q0** the minimum noise is found in the 190 keV VMI with 0.78 ± 1.90 HU. In post hoc testing, a significant difference is found only to the keV in the lower range up to 187 keV (187 keV: corrected p = 0.00275).

In **Q4** the minimum noise is found in the 142 keV VMI with 0.69 ± 0.46 HU. In post hoc testing, a significant difference is found only to the keV in the lower range up to 120 keV (120 keV: corrected p = 0.00194). There is also a significant difference to the higher keV VMI from 170 keV on (170 keV: corrected p = 0.01890).

Selected keV levels with all Q levels and their post hoc tests can be reviewed in the supplemental material (Excel file “PCCT_CCT_Analysis.xlsx”, sheet “analysis_noise”).

## ROI (11): White Matter adjacent to ROI (10) in the Superior Internal Capsule


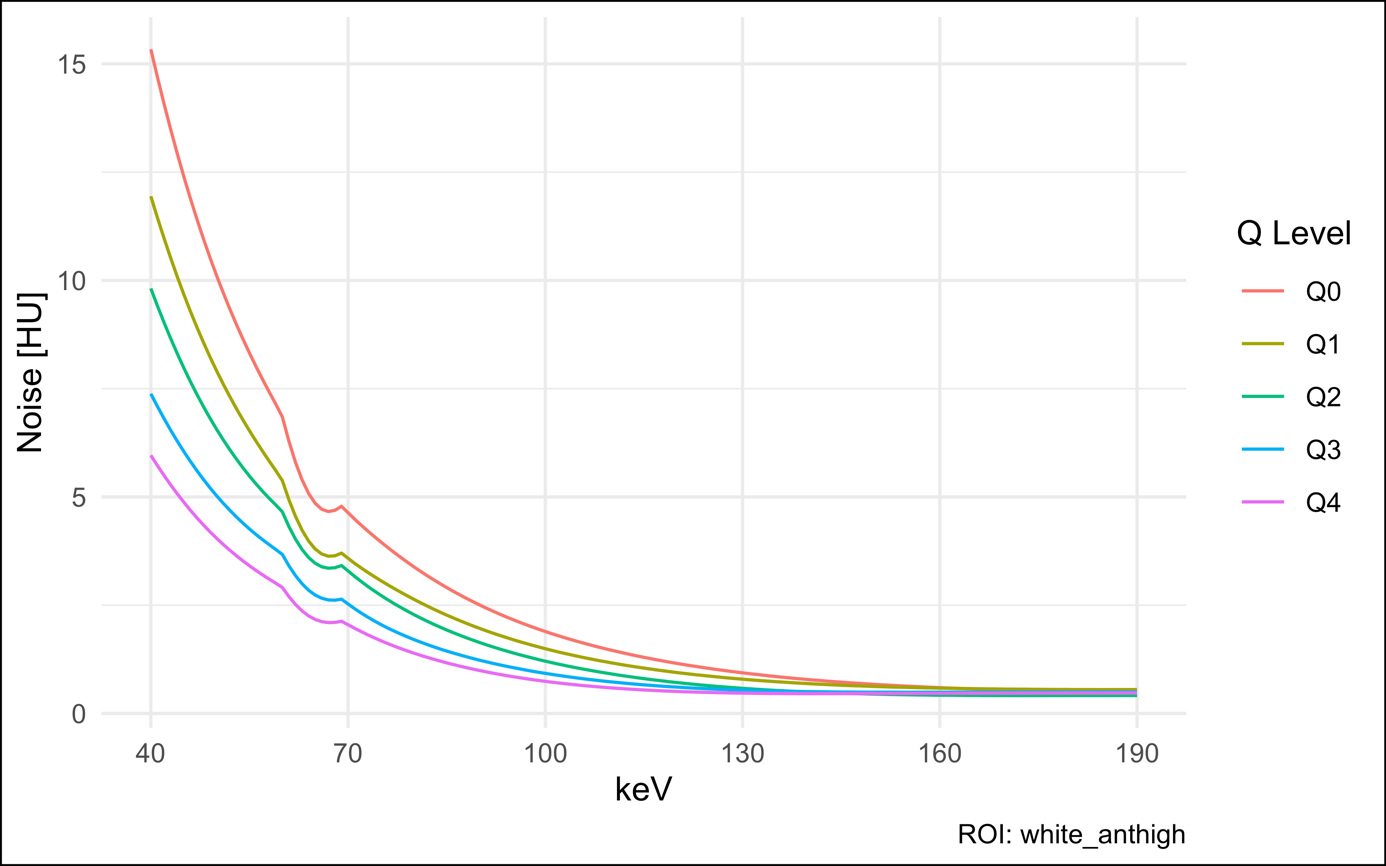


There is a significant difference between the noise characteristics of the individual virtual monoenergetic reconstructions (Friedman test corrected p < 0.0001) in ROI (11). As expected, the iterative reconstruction leads to a reduction of noise with each level. Between 60 and 70 keV, there is a focally emphasized lowering of the noise in each iterative level.

In **Q0** the minimum noise is found in the 190 keV VMI with 0.49 ± 2.92 HU. In post hoc testing, a significant difference is found only to the keV in the lower range up to 188 keV (188 keV: corrected p = 0.00075).

In **Q4** the minimum noise is found in the 140 keV VMI with 0.46 ± 0.18 HU. In post hoc testing, a significant difference is found only to the keV in the lower range up to 130 keV (130 keV: corrected p = 0.03016). There is also a significant difference to the higher keV VMI from 160 keV on (160 keV: corrected p = 0.01594).

Selected keV levels with all Q levels and their post hoc tests can be reviewed in the supplemental material (Excel file “PCCT_CCT_Analysis.xlsx”, sheet “analysis_noise”).

## ROI (12): Gray Matter in the Inferior Caput Nuclei Caudati


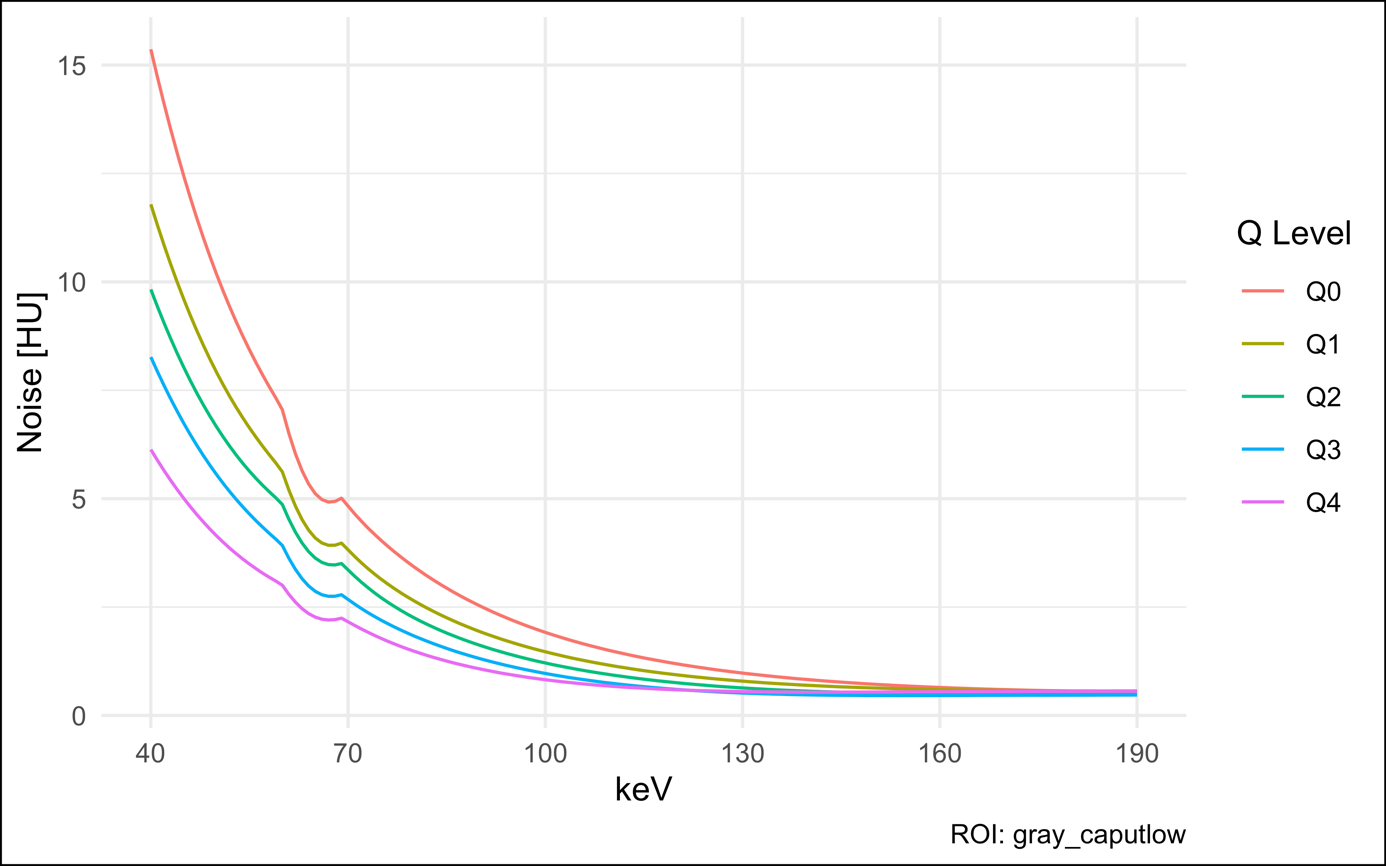


There is a significant difference between the noise characteristics of the individual virtual monoenergetic reconstructions (Friedman test corrected p < 0.0001) in ROI (12). As expected, the iterative reconstruction leads to a reduction of noise with each level. Between 60 and 70 keV, there is a focally emphasized lowering of the noise in each iterative level.

In **Q0** the minimum noise is found in the 190 keV VMI with 0.54 ± 2.98 HU. In post hoc testing, a significant difference is found only to the keV in the lower range up to 188 keV (188 keV: corrected p = 0.00142).

In **Q4** the minimum noise is found in the 142 keV VMI with 0.54 ± 0.29 HU. In post hoc testing, a significant difference is found only to the keV in the lower range up to 130 keV (130 keV: corrected p = 0.03080). There is also a significant difference to the higher keV VMI from 146 keV on (146 keV: corrected p = 0.08625).

Selected keV levels with all Q levels and their post hoc tests can be reviewed in the supplemental material (Excel file “PCCT_CCT_Analysis.xlsx”, sheet “analysis_noise”).

## ROI (13): White Matter adjacent to ROI (12) in the Anterior Callosum


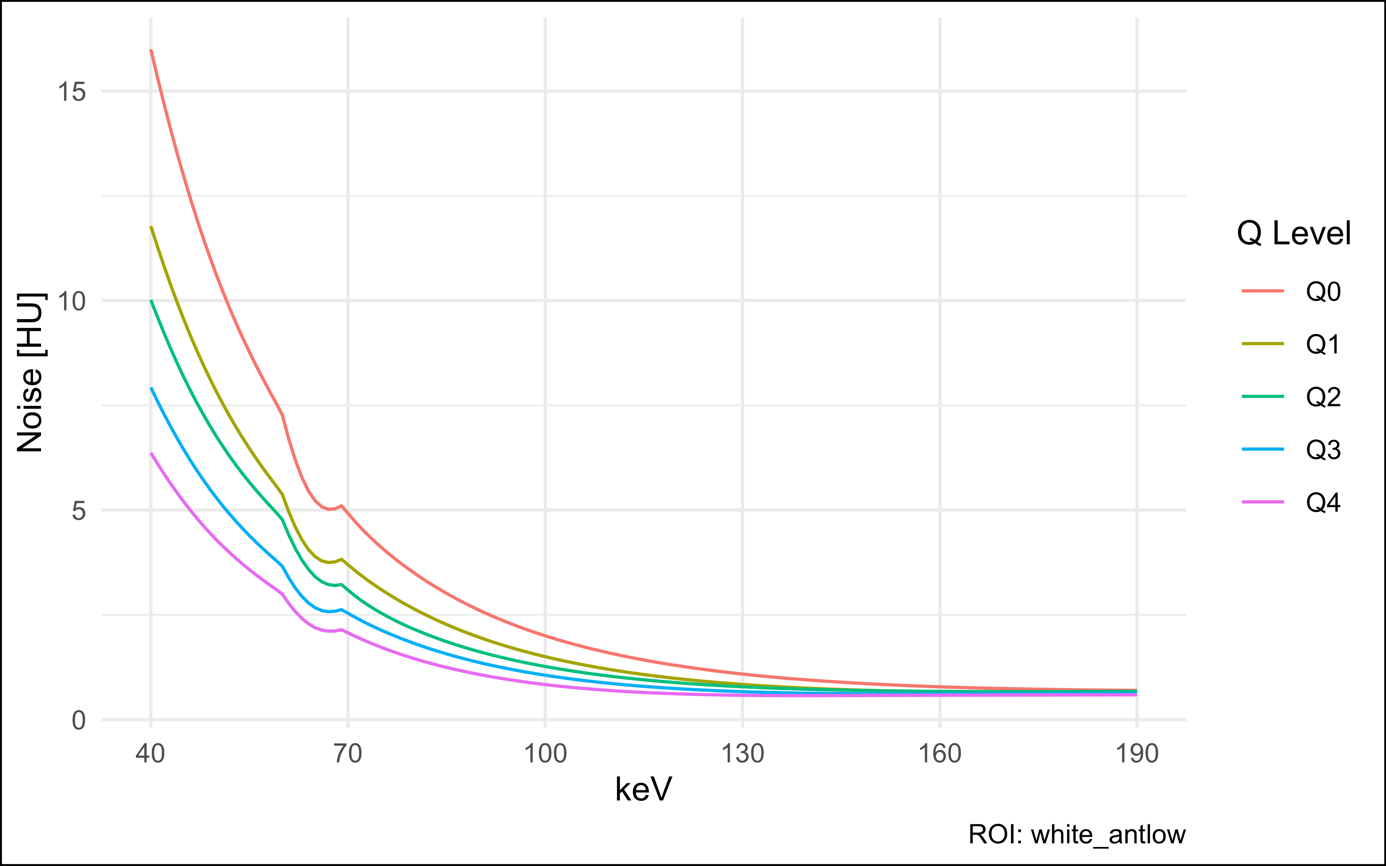


There is a significant difference between the noise characteristics of the individual virtual monoenergetic reconstructions (Friedman test corrected p < 0.0001) in ROI (13). As expected, the iterative reconstruction leads to a reduction of noise with each level. Between 60 and 70 keV, there is a focally emphasized lowering of the noise in each iterative level.

In **Q0** the minimum noise is found in the 190 keV VMI with 0.7 ± 2.90 HU. In post hoc testing, a significant difference is found only to the keV in the lower range up to 188 keV (188 keV: corrected p = 0.01068).

In **Q4** the minimum noise is found in the 137 keV VMI with 0.57 ± 0.32 HU. In post hoc testing, a significant difference is found only to the keV in the lower range up to 130 keV (130 keV: corrected p = 0.03286). There is also a significant difference to the higher keV VMI from 170 keV on (170 keV: corrected p = 0.02468).

Selected keV levels with all Q levels and their post hoc tests can be reviewed in the supplemental material (Excel file “PCCT_CCT_Analysis.xlsx”, sheet “analysis_noise”).

## ROI (14): Gray Matter in the Posterior Thalamus


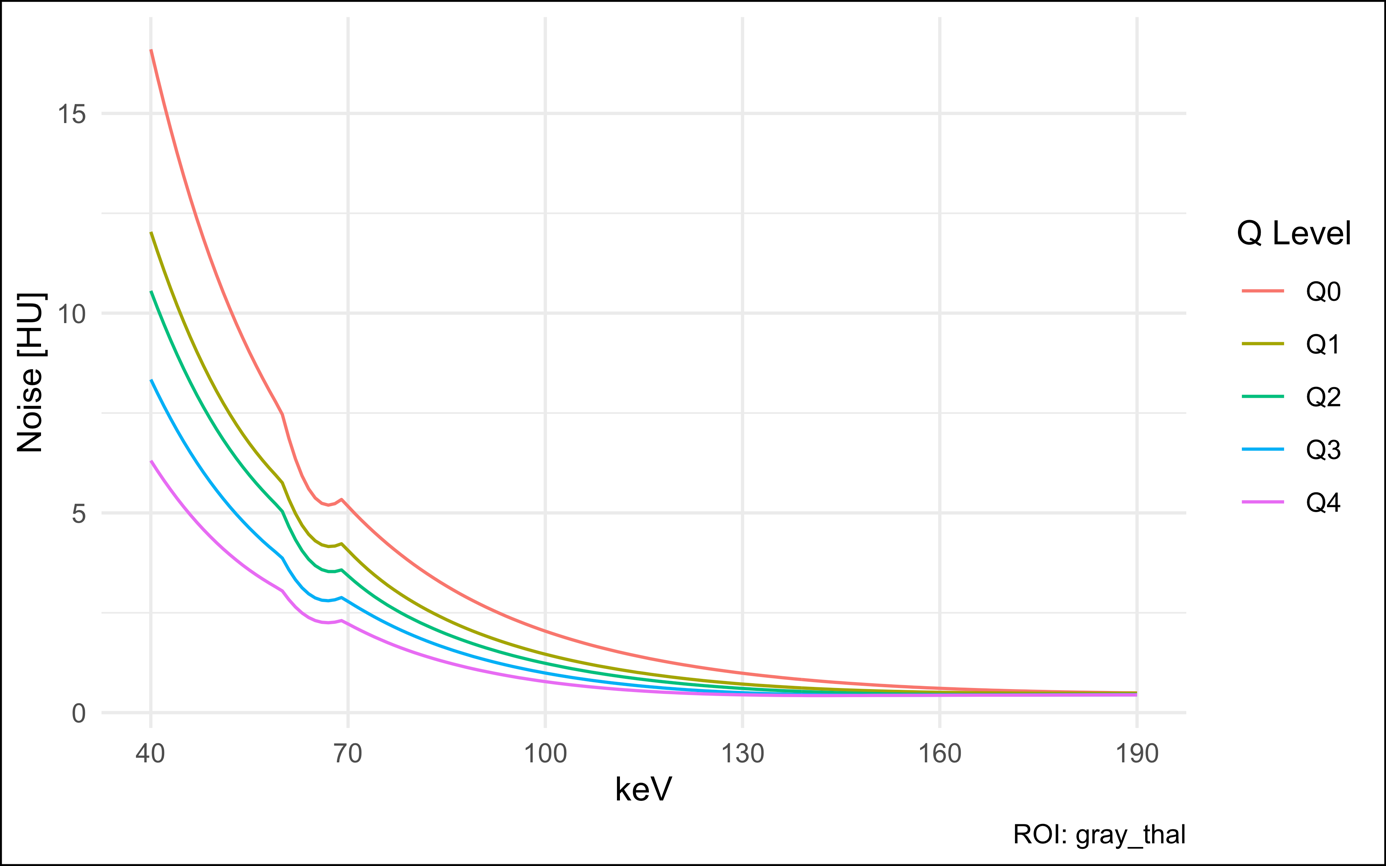


There is a significant difference between the noise characteristics of the individual virtual monoenergetic reconstructions (Friedman test corrected p < 0.0001) in ROI (14). As expected, the iterative reconstruction leads to a reduction of noise with each level. Between 60 and 70 keV, there is a focally emphasized lowering of the noise in each iterative level.

In **Q0** the minimum noise is found in the 190 keV VMI with 0.49 ± 2.85 HU. In post hoc testing, a significant difference is found only to the keV in the lower range up to 189 keV (189 keV: corrected p = 0.01232).

In **Q4** the minimum noise is found in the 142 keV VMI with 0.43 ± 0.14 HU. In post hoc testing, a significant difference is found only to the keV in the lower range up to 138 keV (138 keV: corrected p = 0.02904). There is also a significant difference to the higher keV VMI from 147 keV on (147 keV: corrected p = 0.01589).

Selected keV levels with all Q levels and their post hoc tests can be reviewed in the supplemental material (Excel file “PCCT_CCT_Analysis.xlsx”, sheet “analysis_noise”).

## ROI (15): White Matter in the Posterior Internal Capsule


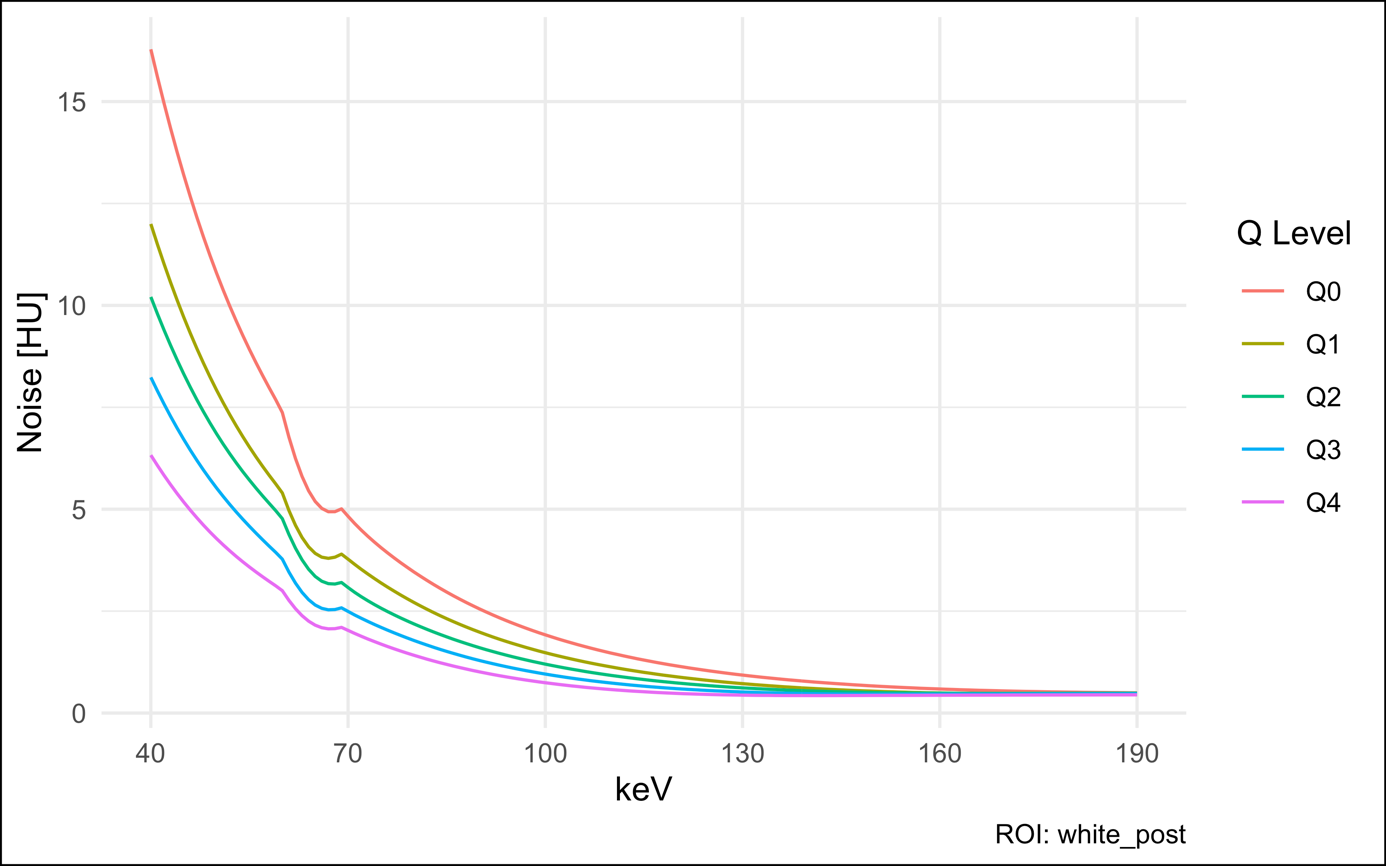


There is a significant difference between the noise characteristics of the individual virtual monoenergetic reconstructions (Friedman test corrected p < 0.0001) in ROI (15). As expected, the iterative reconstruction leads to a reduction of noise with each level. Between 60 and 70 keV, there is a focally emphasized lowering of the noise in each iterative level.

In **Q0** the minimum noise is found in the 190 keV VMI with 0.5 ± 3.67 HU. In post hoc testing, a significant difference is found only to the keV in the lower range up to 188 keV (188 keV: corrected p = 0.00799).

In **Q4** the minimum noise is found in the 142 keV VMI with 0.43 ± 0.17 HU. In post hoc testing, a significant difference is found only to the keV in the lower range up to 120 keV (120 keV: corrected p = 0.00001). There is also a significant difference to the higher keV VMI from 150 keV on (150 keV: corrected p = 0.00467).

Selected keV levels with all Q levels and their post hoc tests can be reviewed in the supplemental material (Excel file “PCCT_CCT_Analysis.xlsx”, sheet “analysis_noise”).

## ROI (16): White Matter in the Pons between the Petrous Bones


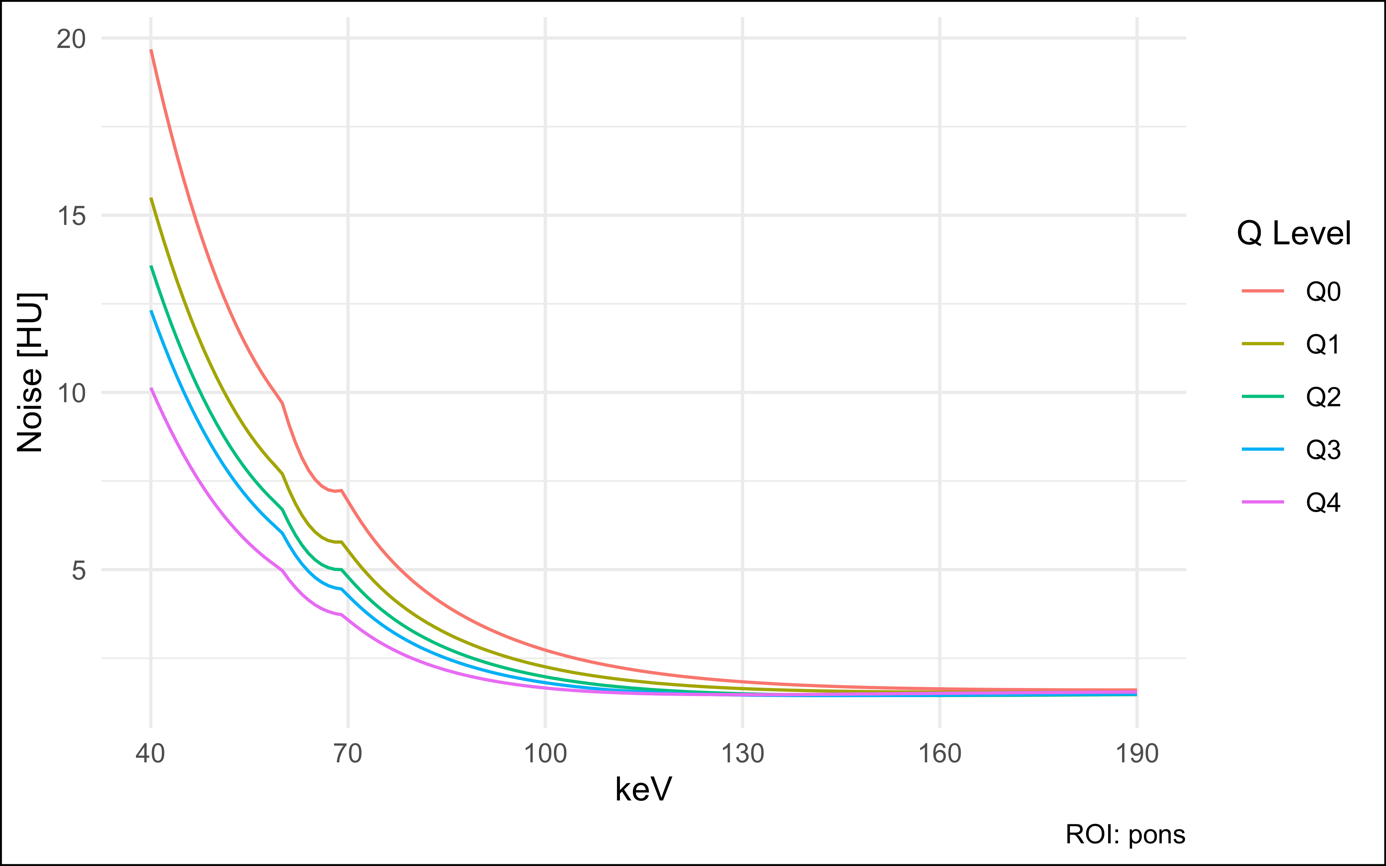


ROI (16) was designed exclusively for the analysis of the noise (as the “posterior fossa artifact index”).

There is a significant difference between the noise characteristics of the individual virtual monoenergetic reconstructions (Friedman test corrected p < 0.0001) in ROI (16). As expected, the iterative reconstruction leads to a reduction of noise with each level. Between 60 and 70 keV, there is a focally emphasized lowering of the noise in each iterative level.

In **Q0** the minimum noise is found in the 189 keV VMI with 1.6 ± 0.67 HU. In post hoc testing, a significant difference is found only to the keV in the lower range up to 160 keV (160 keV: corrected p = 0.00419).

In **Q4** the minimum noise is found in the 129 keV VMI with 1.47 ± 0.62 HU. In post hoc testing, a significant difference is found only to the keV in the lower range up to 110 keV (110 keV: corrected p = 0.00157). There is no significant difference to the higher keV VMI.

Selected keV levels with all Q levels and their post hoc tests can be reviewed in the supplemental material (Excel file “PCCT_CCT_Analysis.xlsx”, sheet “analysis_noise”).

# Results: Noise and Calvaria

There is no relevant difference between the ROIs far from the cranial calvaria in terms of noise as a function of keV. In the deep gray matter, the noise is slightly larger than in the deep white matter.

| 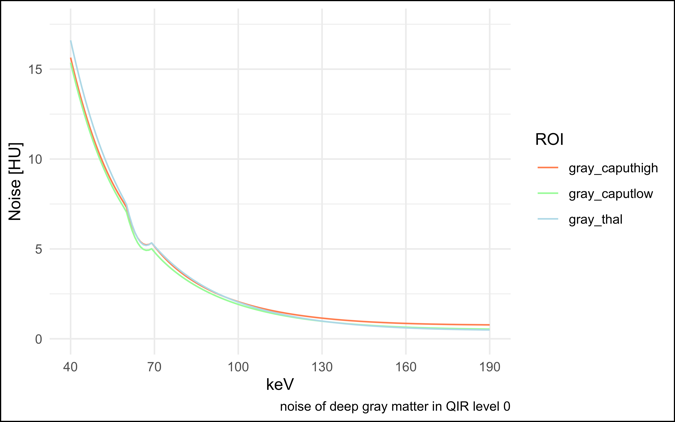 | 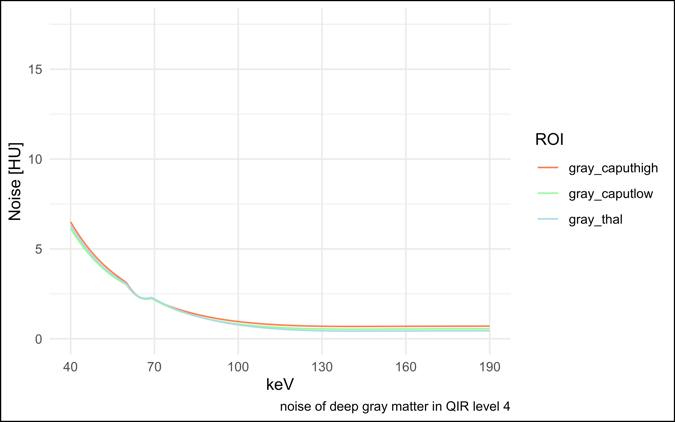 |
| --- | --- |
| 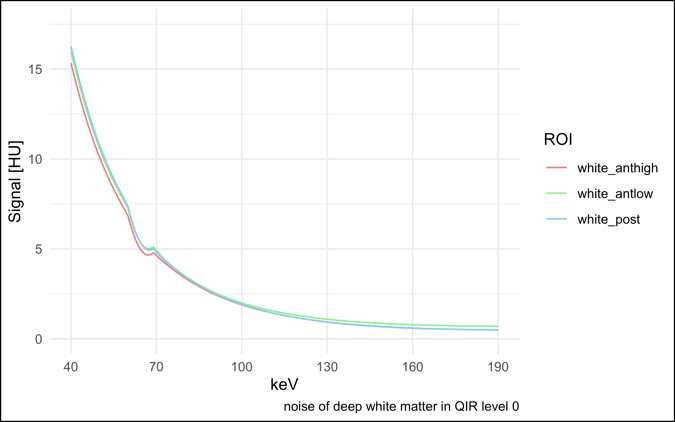 | 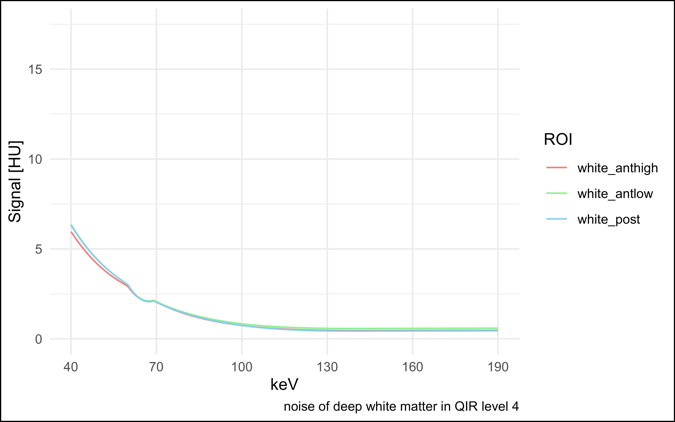 |

In the ROI directly below the cranial dome, the noise is extremely high - interestingly, there was no influence of the QIR here (see above). It is also remarkable that the distance to the calotte does not seem to play a role. The curves of the ROIs from 5 mm to 20 mm below the dome are almost congruent. The influence of the QIR here is comparable to the noise reduction in the ROIs of the deep brain matter.

| 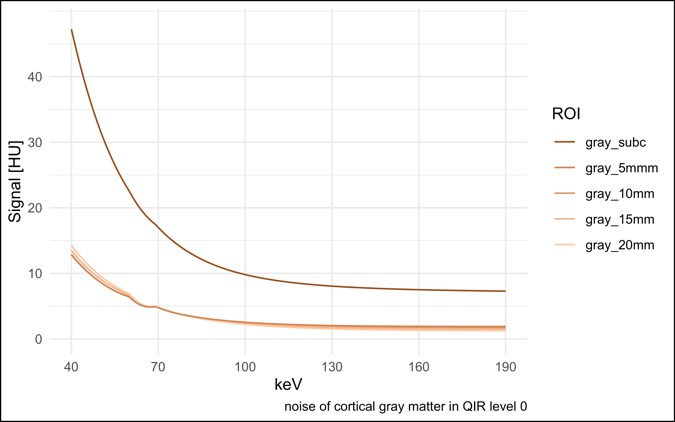 | 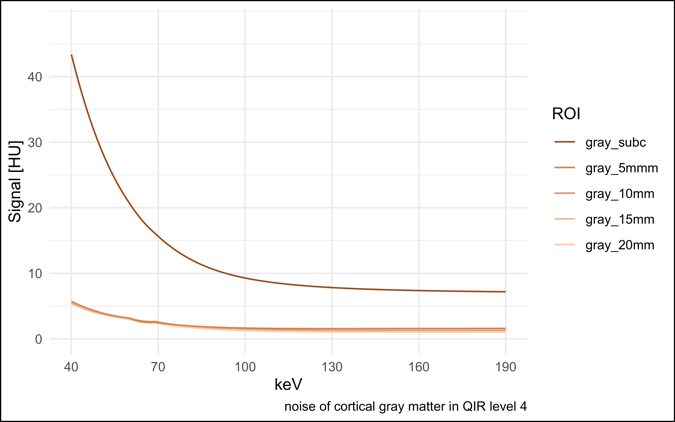 |
| --- | --- |
| 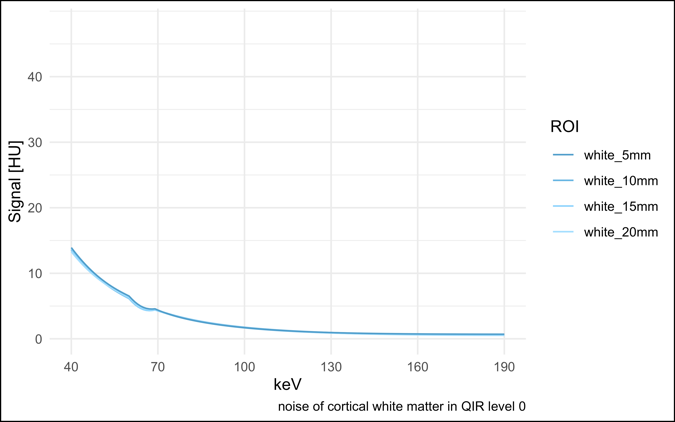 | 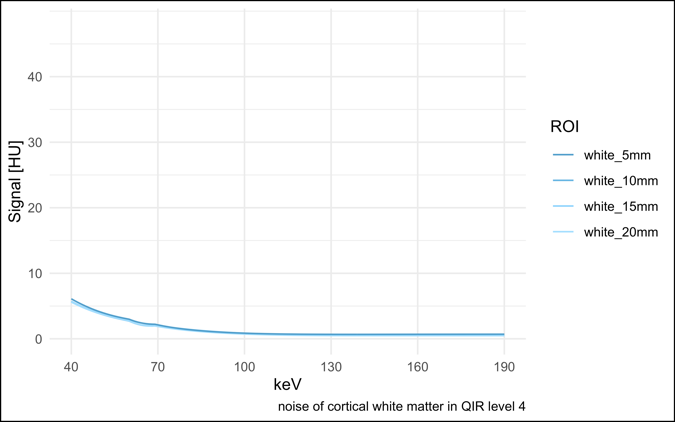 |

No connection can be uncovered with respect to the calotte thickness either. In the linear model, there is a no significant (F-statistic: 1.331 on 1 and 49 df, p-value: 0.2539) correlation between the calvarial thickness and the noise in the ROI in the gray matter 5 mm below the calvaria (tested in Q0).

| 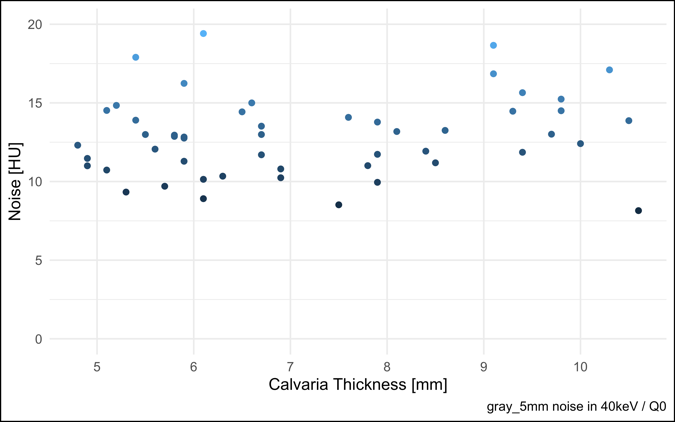 | 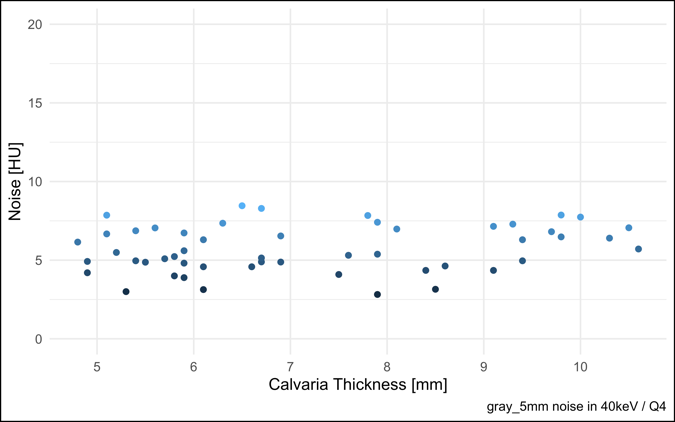 |
| --- | --- |

Comparing the noise on the side with and without cranial calvaria in patients with craniectomy, only a trend is evident here; the difference between the groups (with cranial calvaria 13.29 ± 2.28 HU, without 12.06 ± 1.96 HU) is not significant in this approach (T test: p = 0.07517). However, it seems likely that with a larger number of observation units, a small difference can be worked out; the relevance of this small difference as opposed to the larger difference in signal would have to be discussed separately.


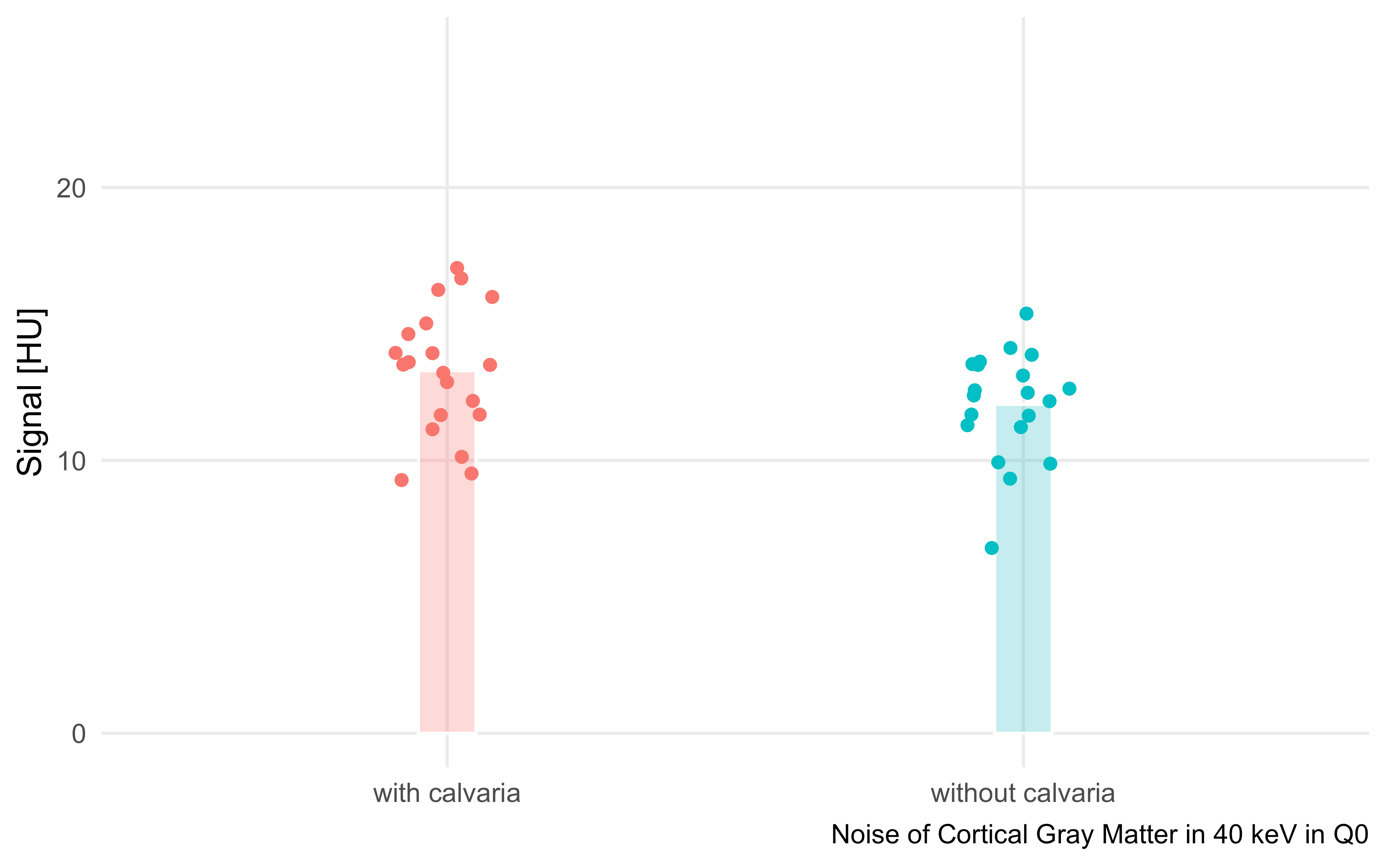


# Results: Signal to Noise Ratio (SNR)

## ROI (1): Gray Matter below the Calvaria


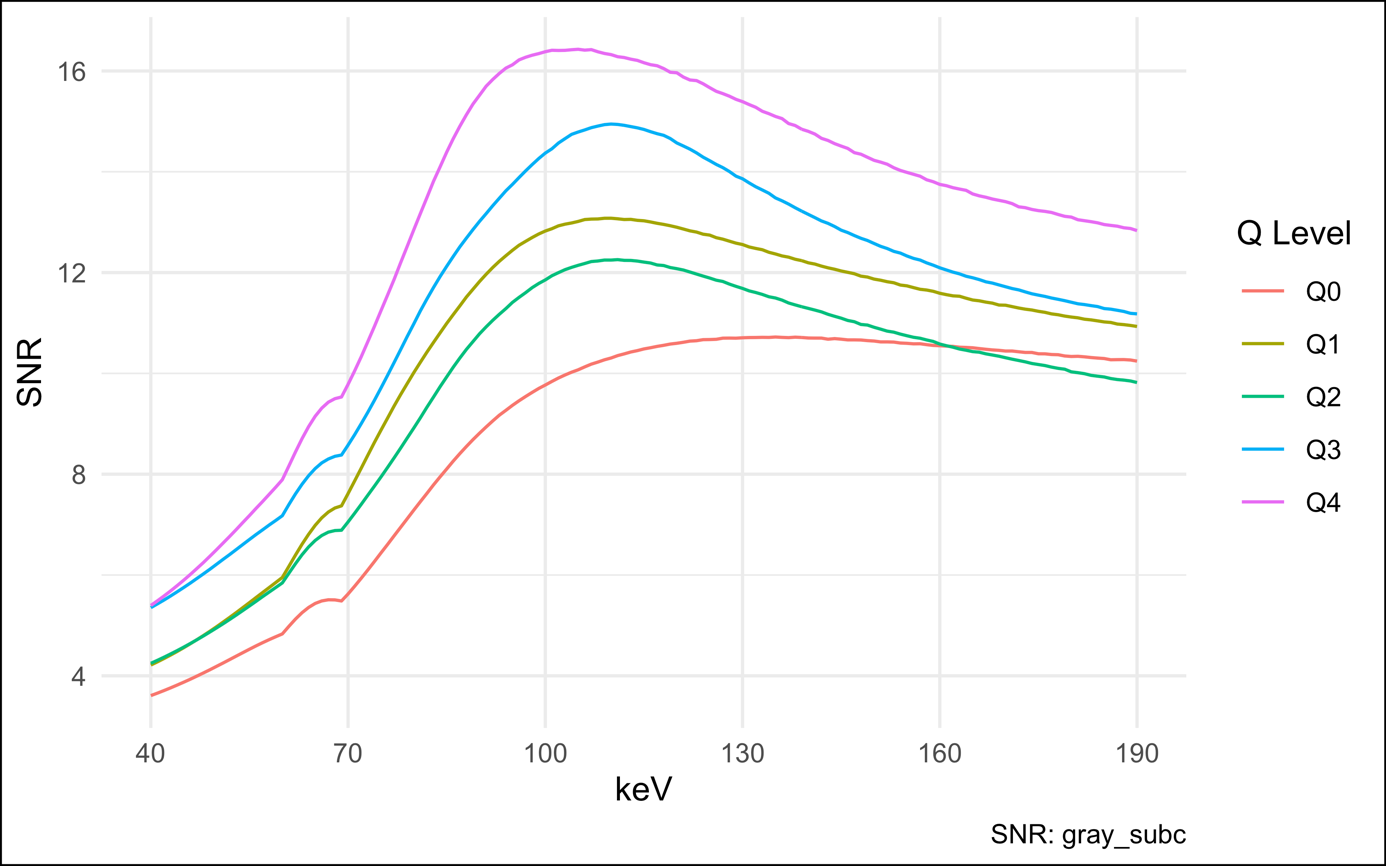


There is a significant difference between the SNR characteristics of the individual virtual monoenergetic reconstructions (Friedman test corrected p < 0.0001) in ROI (1). The small difference in noise between the different Q levels seems noticeable in the SNR - since it is a quotient. According to the noise decreasing with each Q level, the SNR increases. The maximum SNR in Q4 is found in the 135 keV VMI with 10.72 ± 9.64. The focally further reduced noise between 60 and 70 keV results in focally increased SNR.

In post hoc testing, a significant difference is found to the keV in the lower range up to 80 keV (80 keV: corrected p = 0.003753). There is no significant difference to the SNR in higher keV VMI.

Selected keV levels with all Q levels and their post hoc tests can be reviewed in the supplemental material (Excel file “PCCT_CCT_Analysis.xlsx”, sheet “analysis_snr”).

## ROI (2): Cortical Gray Matter 5 mm below the Calvaria


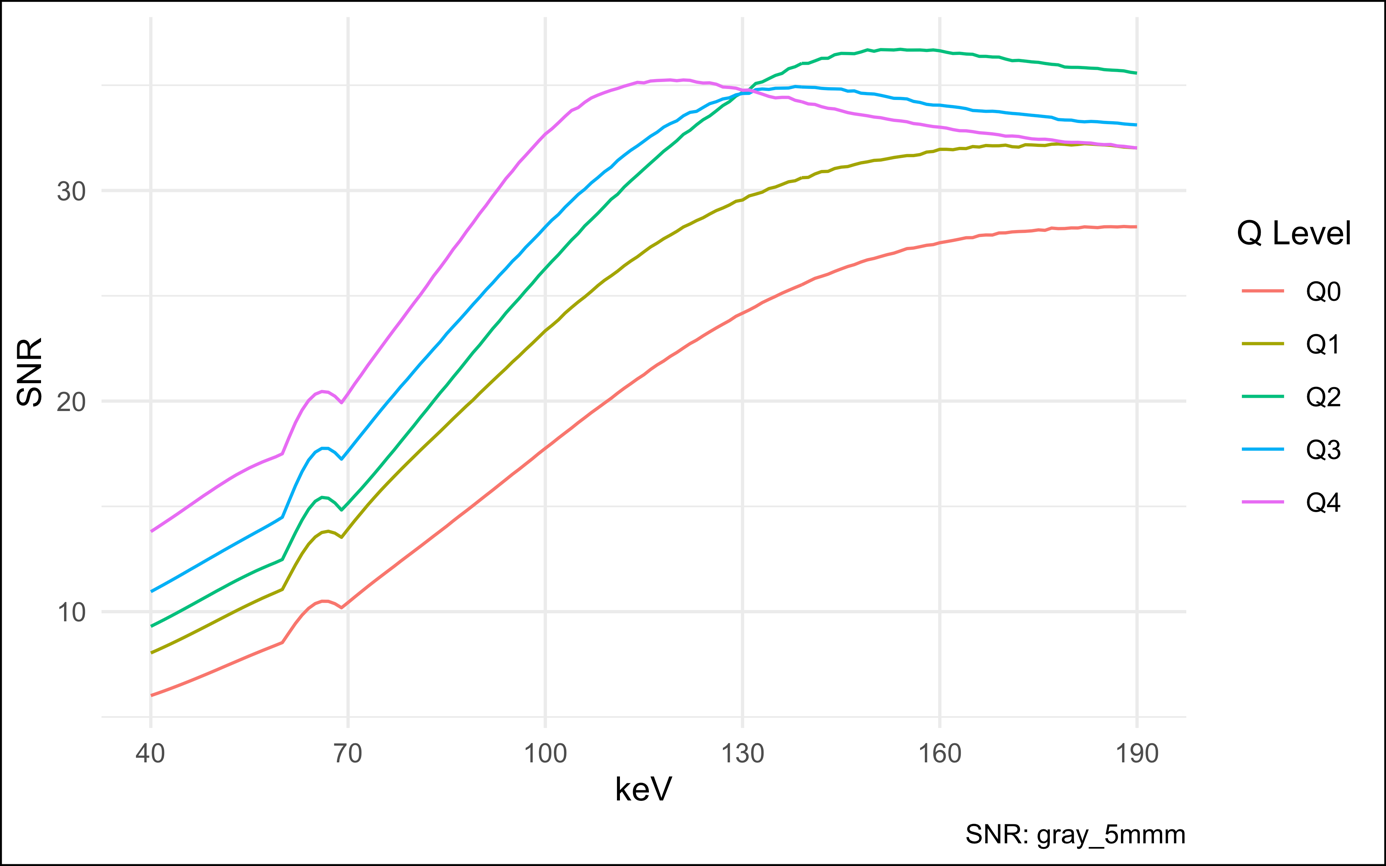


There is a significant difference between the SNR characteristics of the individual virtual monoenergetic reconstructions (Friedman test corrected p < 0.0001) in ROI (2). According to the noise decreasing with each Q level, the SNR increases. The maximum SNR in Q4 is found in the 119 keV VMI with 35.25 ± 24.47. The focally further reduced noise between 60 and 70 keV results in focally increased SNR.

In post hoc testing, a significant difference is found to the keV in the lower range up to 80 keV (80 keV: corrected p = 0.001632) and to the keV in higher range from 180 keV on (180 keV: corrected p = 0.02928).

Selected keV levels with all Q levels and their post hoc tests can be reviewed in the supplemental material (Excel file “PCCT_CCT_Analysis.xlsx”, sheet “analysis_snr”).

## ROI (3): Cortical White Matter 5 mm below the Calvaria


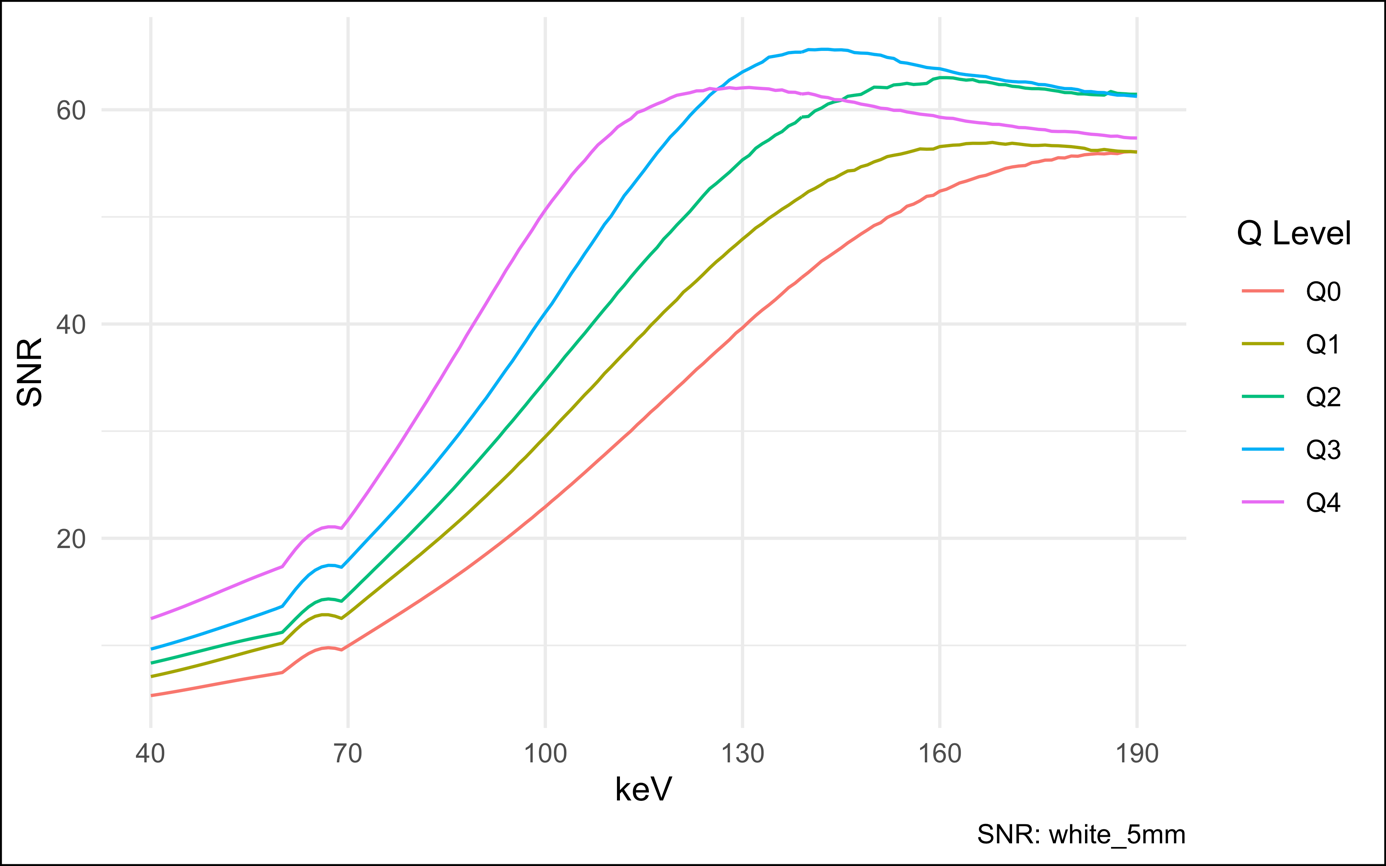


There is a significant difference between the SNR characteristics of the individual virtual monoenergetic reconstructions (Friedman test corrected p < 0.0001) in ROI (3). According to the noise decreasing with each Q level, the SNR increases. The maximum SNR in Q4 is found in the 119 keV VMI with 62.08 ± 26.01. The focally further reduced noise between 60 and 70 keV results in focally increased SNR.

In post hoc testing, a significant difference is found to the keV in the lower range up to 105 keV (105 keV: corrected p = 0.009906). There is no significant difference to the SNR in higher keV VMI.

Selected keV levels with all Q levels and their post hoc tests can be reviewed in the supplemental material (Excel file “PCCT_CCT_Analysis.xlsx”, sheet “analysis_snr”).

## ROI (4): Cortical Gray Matter 10 mm below the Calvaria


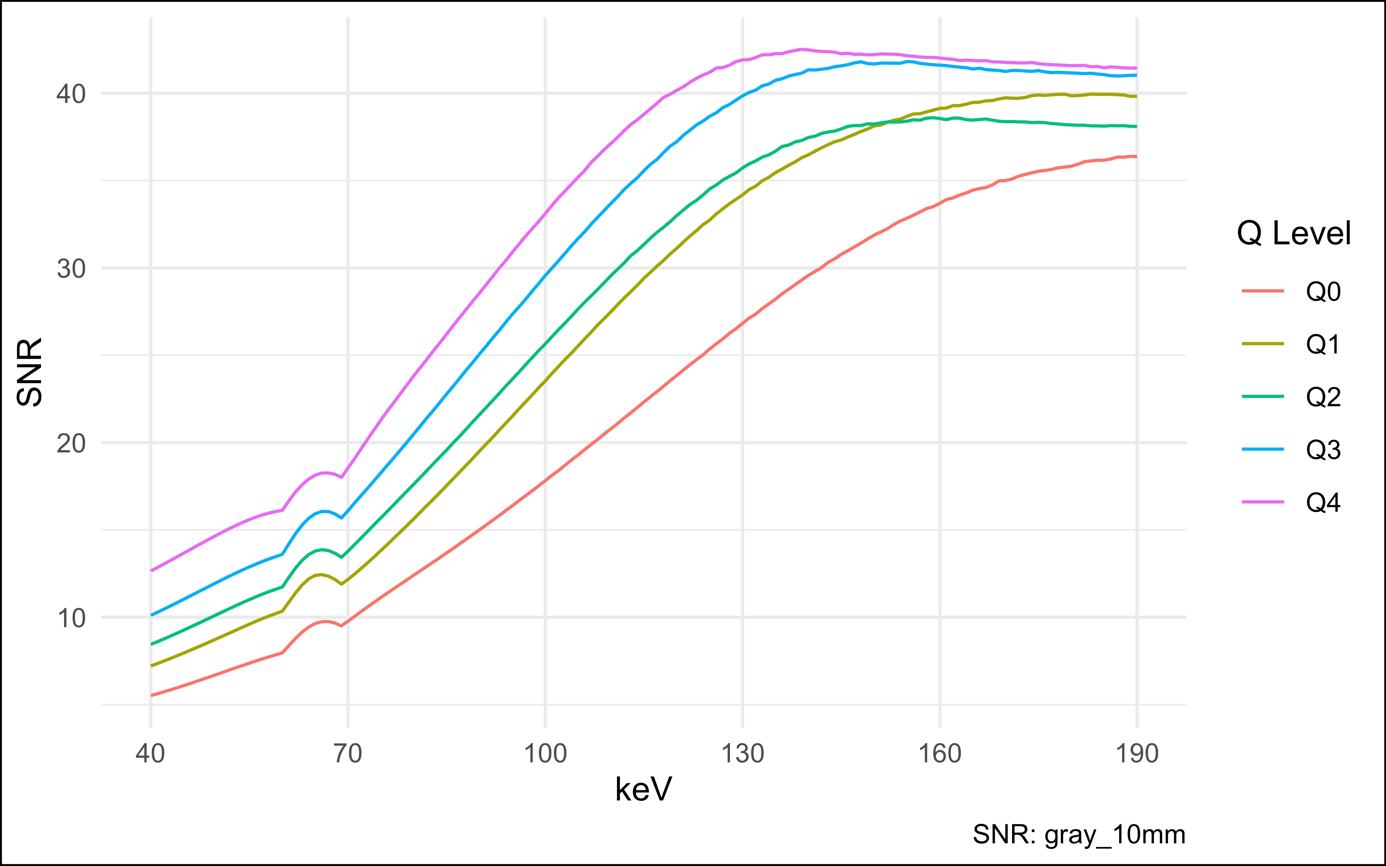


There is a significant difference between the SNR characteristics of the individual virtual monoenergetic reconstructions (Friedman test corrected p < 0.0001) in ROI (4). According to the noise decreasing with each Q level, the SNR increases. The maximum SNR in Q4 is found in the 119 keV VMI with 42.51 ± 26.66. The focally further reduced noise between 60 and 70 keV results in focally increased SNR.

In post hoc testing, a significant difference is found to the keV in the lower range up to 105 keV (105 keV: corrected p = 0.006865). There is no significant difference to the SNR in higher keV VMI.

Selected keV levels with all Q levels and their post hoc tests can be reviewed in the supplemental material (Excel file “PCCT_CCT_Analysis.xlsx”, sheet “analysis_snr”).

## ROI (5): White Matter 10 mm below the Calvaria


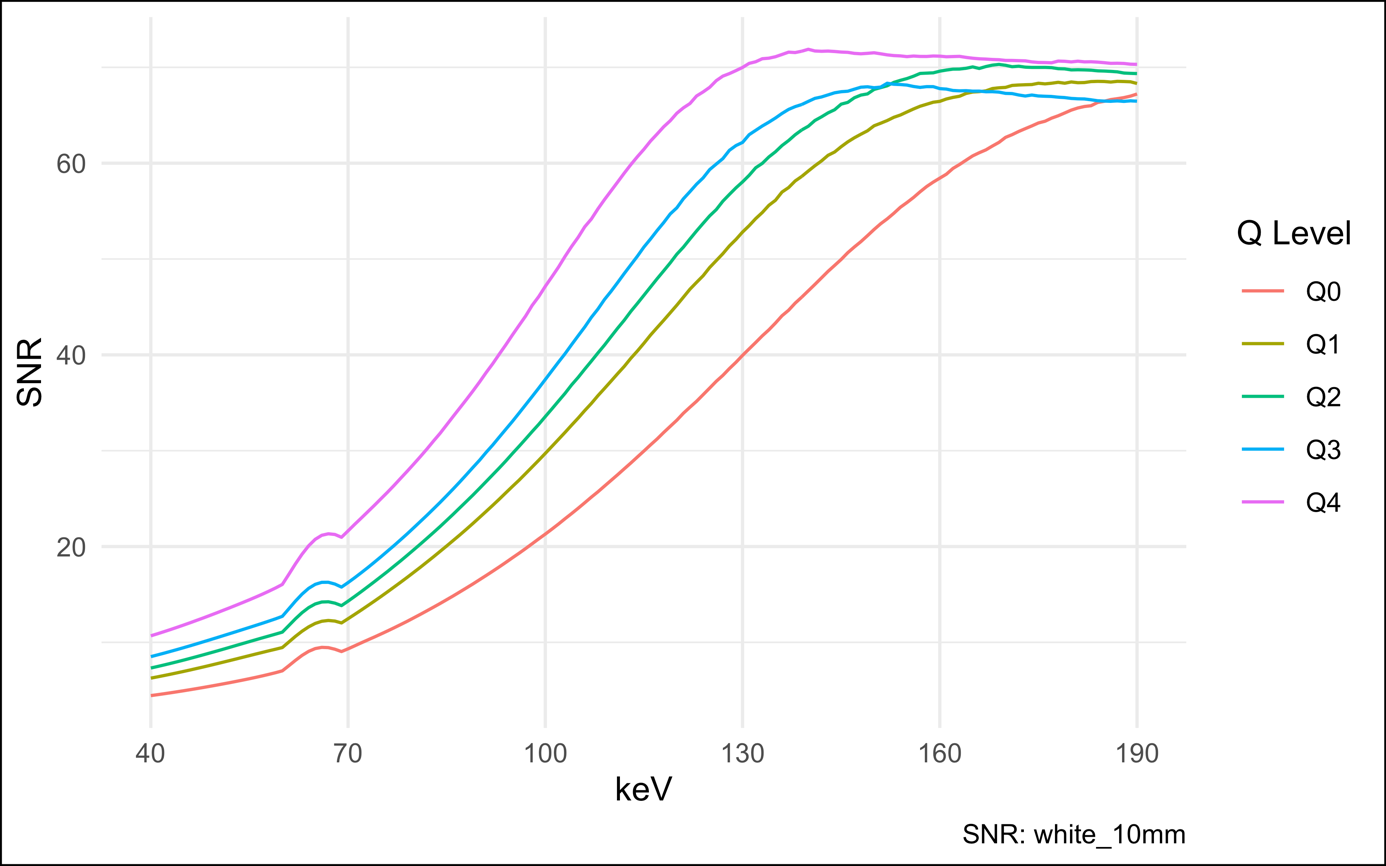


There is a significant difference between the SNR characteristics of the individual virtual monoenergetic reconstructions (Friedman test corrected p < 0.0001) in ROI (5). According to the noise decreasing with each Q level, the SNR increases. The maximum SNR in Q4 is found in the 119 keV VMI with 71.88 ± 32.07. The focally further reduced noise between 60 and 70 keV results in focally increased SNR.

In post hoc testing, a significant difference is found to the keV in the lower range up to 120 keV (120 keV: corrected p = 0.002862). There is no significant difference to the SNR in higher keV VMI.

Selected keV levels with all Q levels and their post hoc tests can be reviewed in the supplemental material (Excel file “PCCT_CCT_Analysis.xlsx”, sheet “analysis_snr”).

## ROI (6): Cortical Gray Matter 15 mm below the Calvaria


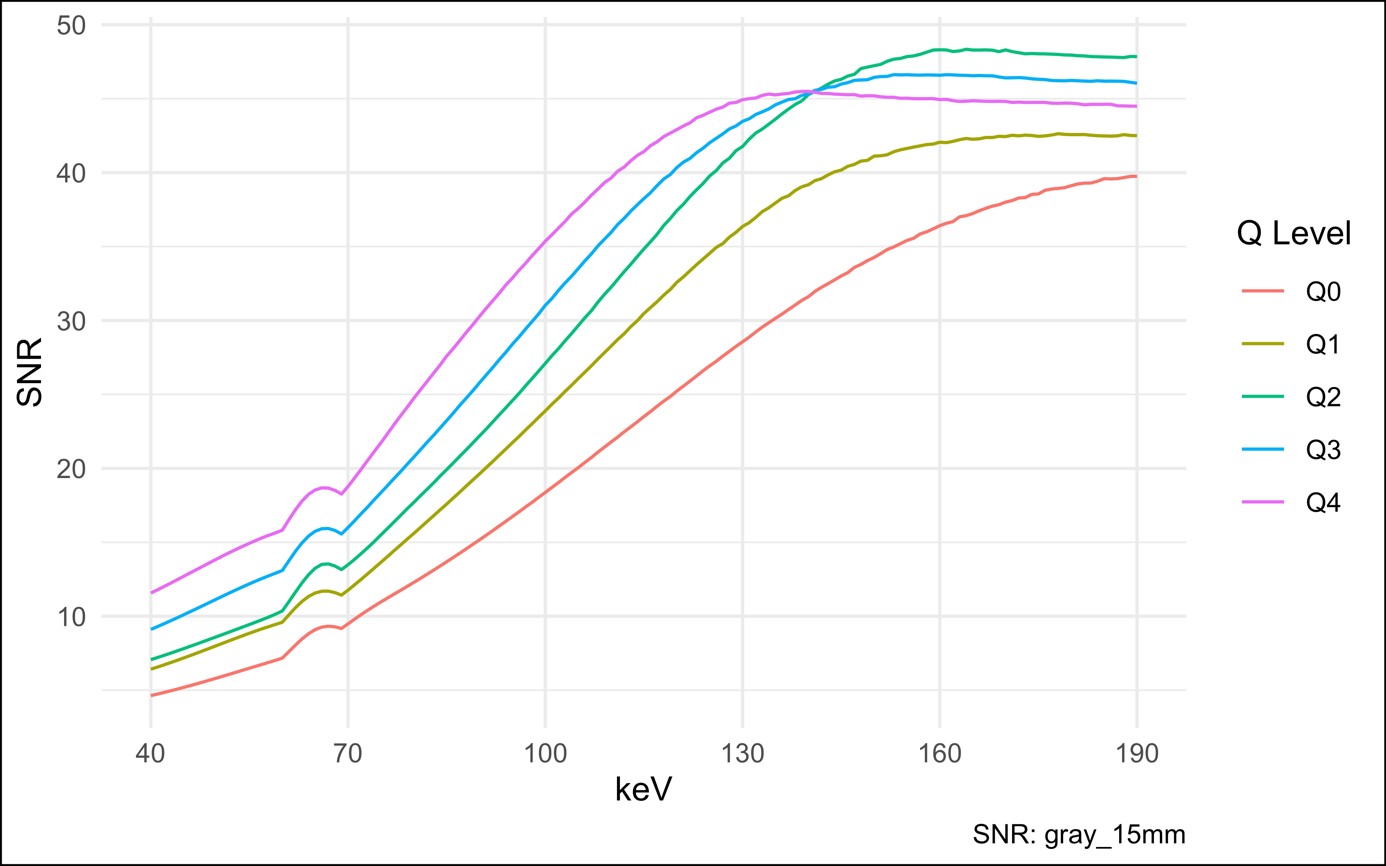


There is a significant difference between the SNR characteristics of the individual virtual monoenergetic reconstructions (Friedman test corrected p < 0.0001) in ROI (6). According to the noise decreasing with each Q level, the SNR increases. The maximum SNR in Q4 is found in the 119 keV VMI with 45.49 ± 27.22. The focally further reduced noise between 60 and 70 keV results in focally increased SNR.

In post hoc testing, a significant difference is found to the keV in the lower range up to 120 keV (120 keV: corrected p = 0.043617). There is no significant difference to the SNR in higher keV VMI.

Selected keV levels with all Q levels and their post hoc tests can be reviewed in the supplemental material (Excel file “PCCT_CCT_Analysis.xlsx”, sheet “analysis_snr”).

## ROI (7): Cortical White Matter 15 mm below the Calvaria


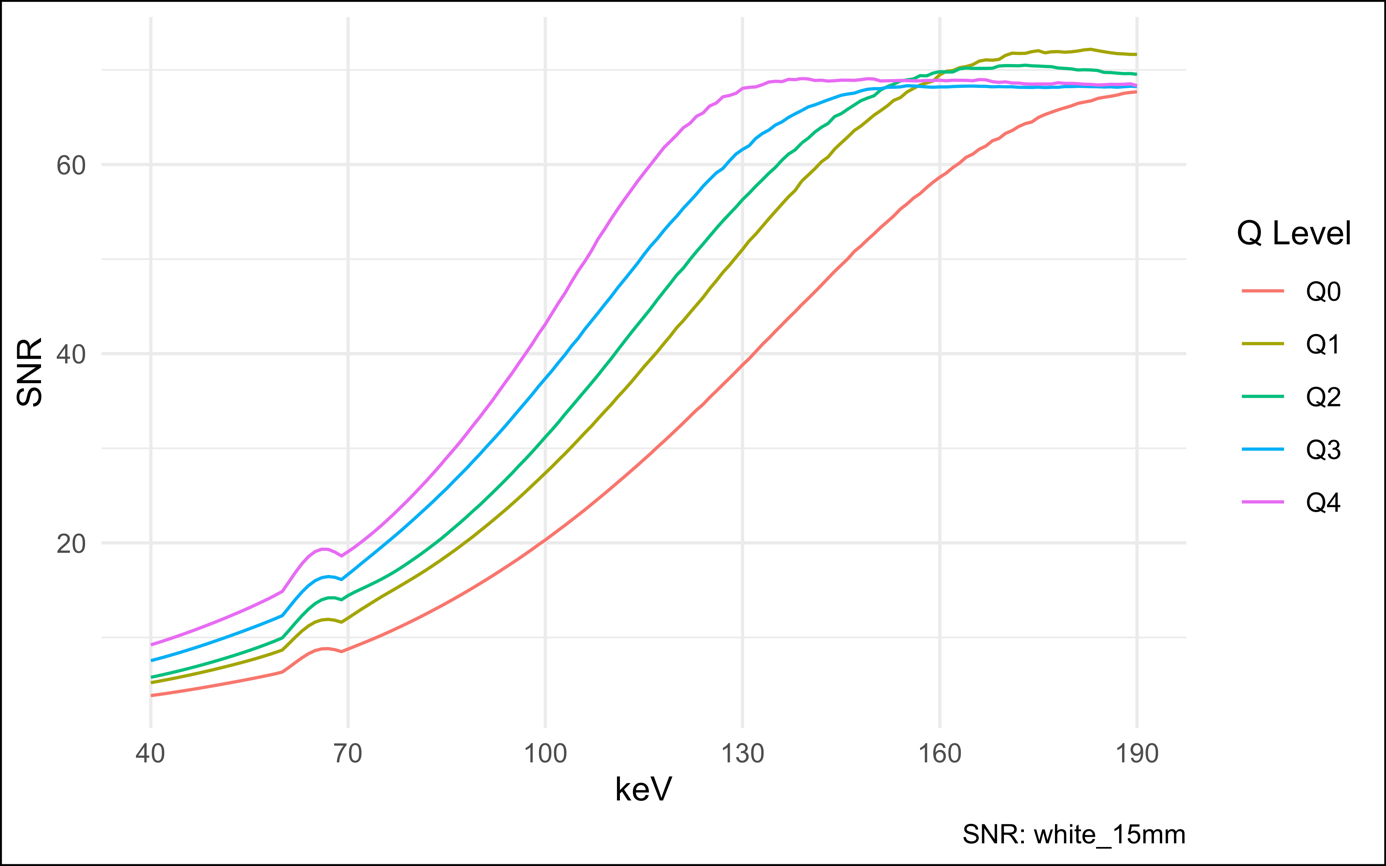


There is a significant difference between the SNR characteristics of the individual virtual monoenergetic reconstructions (Friedman test corrected p < 0.0001) in ROI (7). According to the noise decreasing with each Q level, the SNR increases. The maximum SNR in Q4 is found in the 139 keV VMI with 69.09 ± 29.40. The focally further reduced noise between 60 and 70 keV results in focally increased SNR.

In post hoc testing, a significant difference is found to the keV in the lower range up to 120 keV (80 keV: corrected p = 0.000166). There is no significant difference to the SNR in higher keV VMI.

Selected keV levels with all Q levels and their post hoc tests can be reviewed in the supplemental material (Excel file “PCCT_CCT_Analysis.xlsx”, sheet “analysis_snr”).

## ROI (8): Cortical Gray Matter 20 mm below the Calvaria


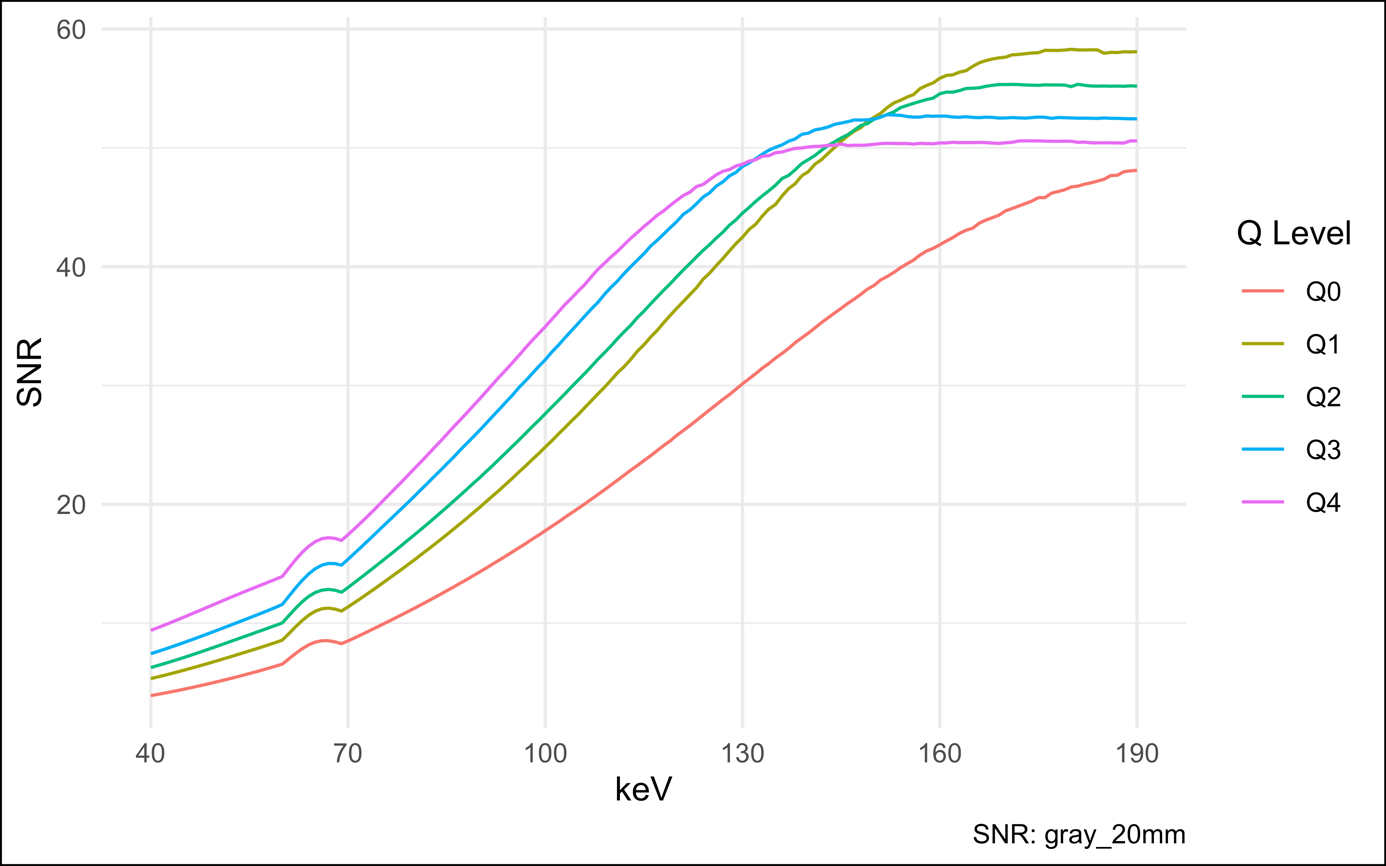


There is a significant difference between the SNR characteristics of the individual virtual monoenergetic reconstructions (Friedman test corrected p < 0.0001) in ROI (8). According to the noise decreasing with each Q level, the SNR increases. The maximum SNR in Q4 is found in the 119 keV VMI with 50.59 ± 30.30. The focally further reduced noise between 60 and 70 keV results in focally increased SNR.

In post hoc testing, a significant difference is found to the keV in the lower range up to 187 keV (187 keV: corrected p = 0.000455). There is no significant difference to the SNR in higher keV VMI.

Selected keV levels with all Q levels and their post hoc tests can be reviewed in the supplemental material (Excel file “PCCT_CCT_Analysis.xlsx”, sheet “analysis_snr”).

## ROI (9): Cortical White Matter 20 mm below the Calvaria


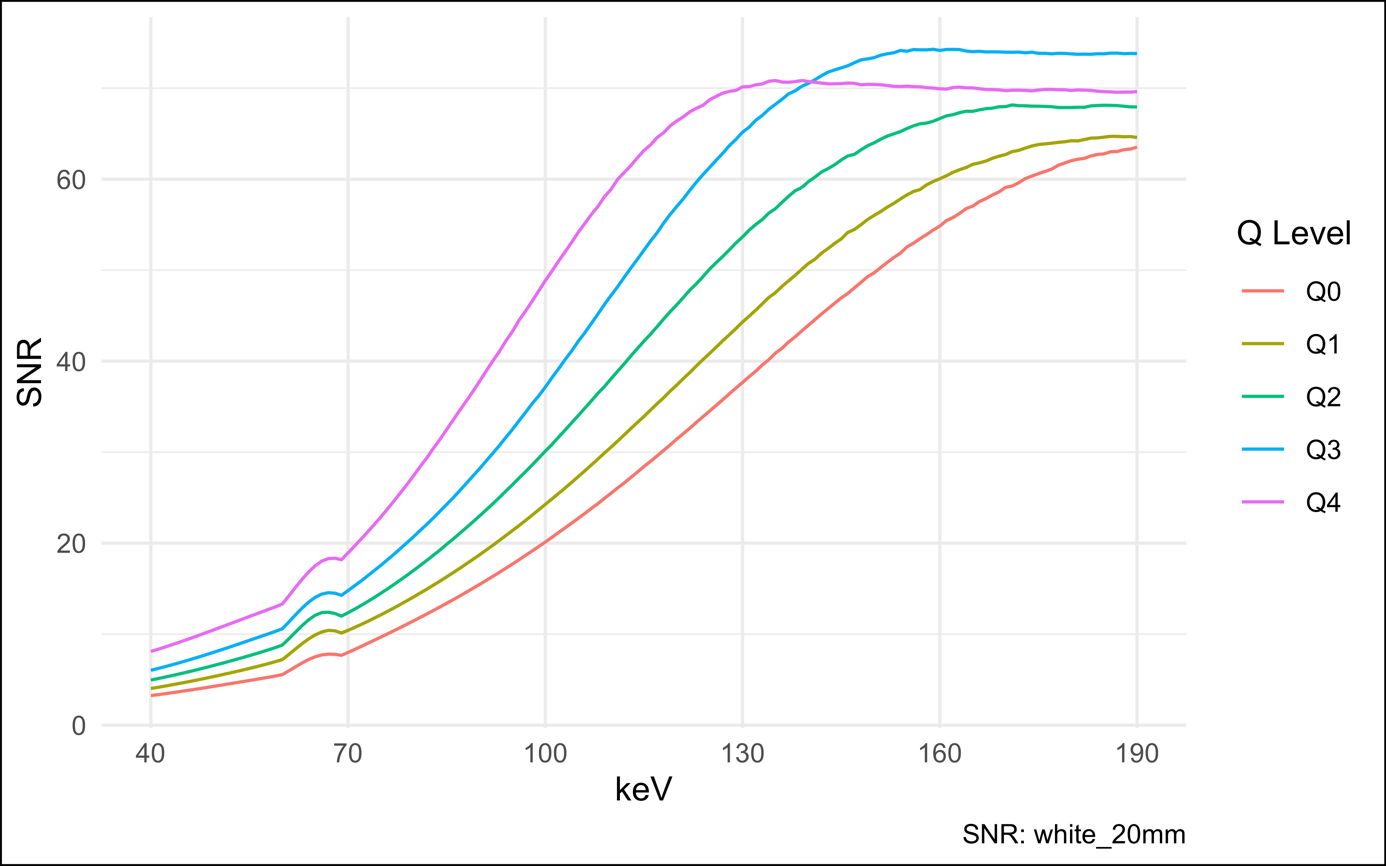


There is a significant difference between the SNR characteristics of the individual virtual monoenergetic reconstructions (Friedman test corrected p < 0.0001) in ROI (9). According to the noise decreasing with each Q level, the SNR increases. The maximum SNR in Q4 is found in the 139 keV VMI with 70.84 ± 23.63. The focally further reduced noise between 60 and 70 keV results in focally increased SNR.

In post hoc testing, a significant difference is found to the keV in the lower range up to 120 keV (120 keV: corrected p = 0.004719). There is no significant difference to the SNR in higher keV VMI.

Selected keV levels with all Q levels and their post hoc tests can be reviewed in the supplemental material (Excel file “PCCT_CCT_Analysis.xlsx”, sheet “analysis_snr”).

## ROI (10): Gray Matter in the Superior Caput Nuclei Caudati


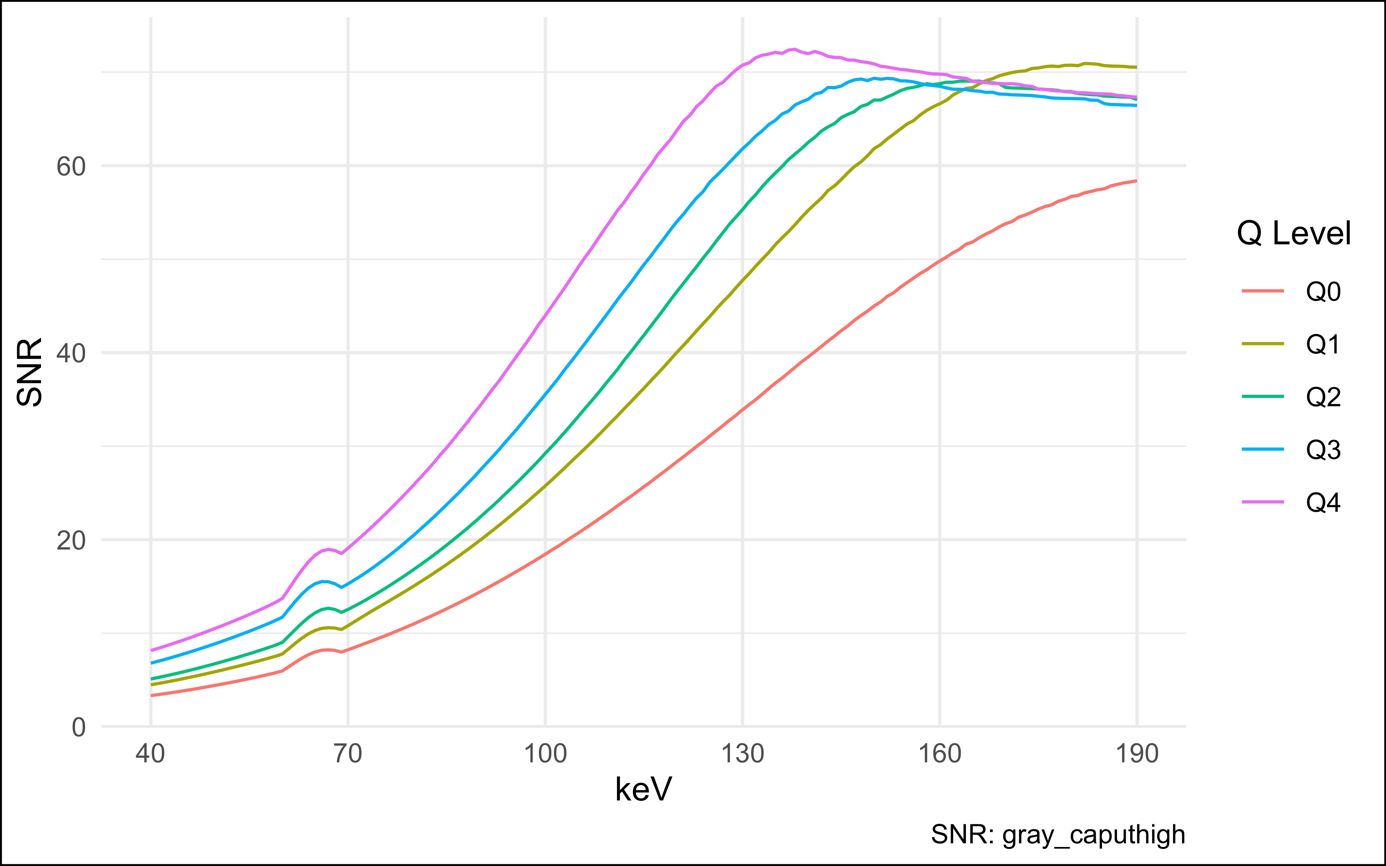


There is a significant difference between the SNR characteristics of the individual virtual monoenergetic reconstructions (Friedman test corrected p < 0.0001) in ROI (10). According to the noise decreasing with each Q level, the SNR increases. The maximum SNR in Q4 is found in the 138 keV VMI with 72.44 ± 42.69. The focally further reduced noise between 60 and 70 keV results in focally increased SNR.

In post hoc testing, a significant difference is found to the keV in the lower range up to 137 keV (137 keV: corrected p = 0.001929) and to the keV in higher range from 150 keV on (150 keV: corrected p = 0.049679).

Selected keV levels with all Q levels and their post hoc tests can be reviewed in the supplemental material (Excel file “PCCT_CCT_Analysis.xlsx”, sheet “analysis_snr”).

## ROI (11): White Matter adjacent to ROI (10) in the Superior Internal Capsule


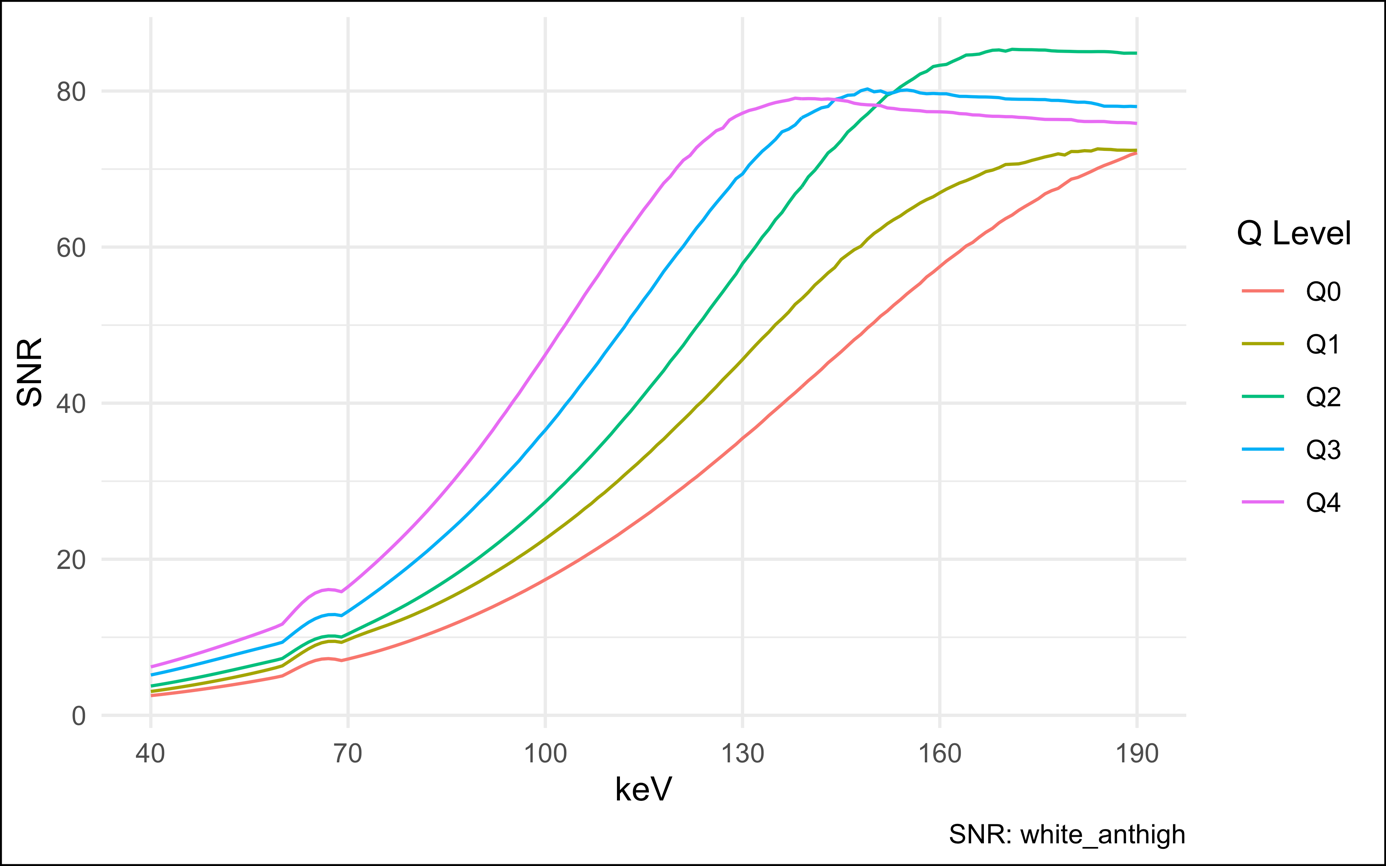


There is a significant difference between the SNR characteristics of the individual virtual monoenergetic reconstructions (Friedman test corrected p < 0.0001) in ROI (11). According to the noise decreasing with each Q level, the SNR increases. The maximum SNR in Q4 is found in the 138 keV VMI with 79.08 ± 30.11. The focally further reduced noise between 60 and 70 keV results in focally increased SNR.

In post hoc testing, a significant difference is found to the keV in the lower range up to 130 keV (130 keV: corrected p = 0.017507) and to the keV in higher range from 170 keV on (170 keV: corrected p = 0.004901).

Selected keV levels with all Q levels and their post hoc tests can be reviewed in the supplemental material (Excel file “PCCT_CCT_Analysis.xlsx”, sheet “analysis_snr”).

## ROI (12): Gray Matter in the Inferior Caput Nuclei Caudati


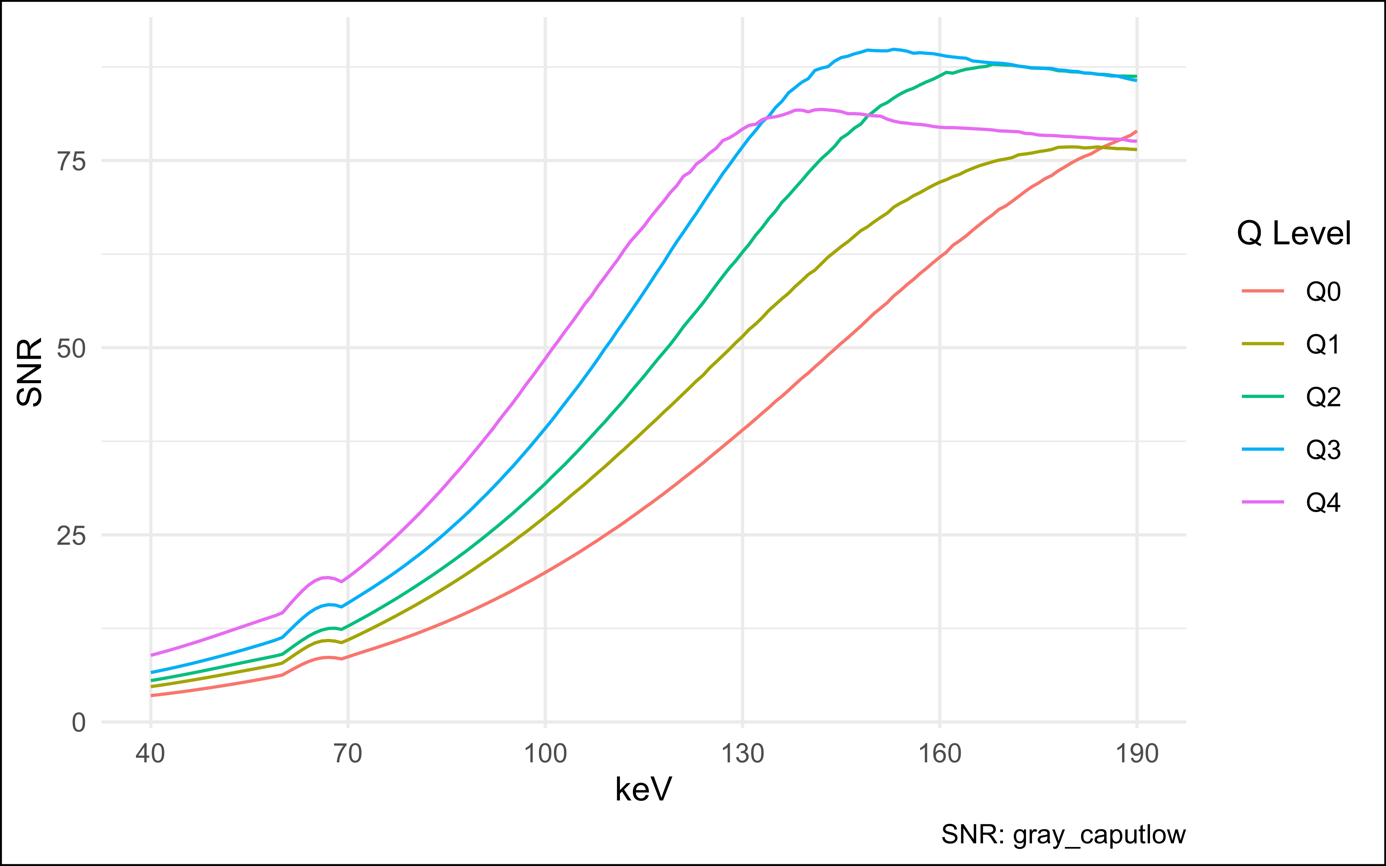


There is a significant difference between the SNR characteristics of the individual virtual monoenergetic reconstructions (Friedman test corrected p < 0.0001) in ROI (12). According to the noise decreasing with each Q level, the SNR increases. The maximum SNR in Q4 is found in the 142 keV VMI with 81.83 ± 38.73. The focally further reduced noise between 60 and 70 keV results in focally increased SNR.

In post hoc testing, a significant difference is found to the keV in the lower range up to 120 keV (120 keV: corrected p = 0.000032) and to the keV in higher range from 143 keV on (143 keV: corrected p = 0.000000).

Selected keV levels with all Q levels and their post hoc tests can be reviewed in the supplemental material (Excel file “PCCT_CCT_Analysis.xlsx”, sheet “analysis_snr”).

## ROI (13): White Matter adjacent to ROI (12) in the Anterior Callosum


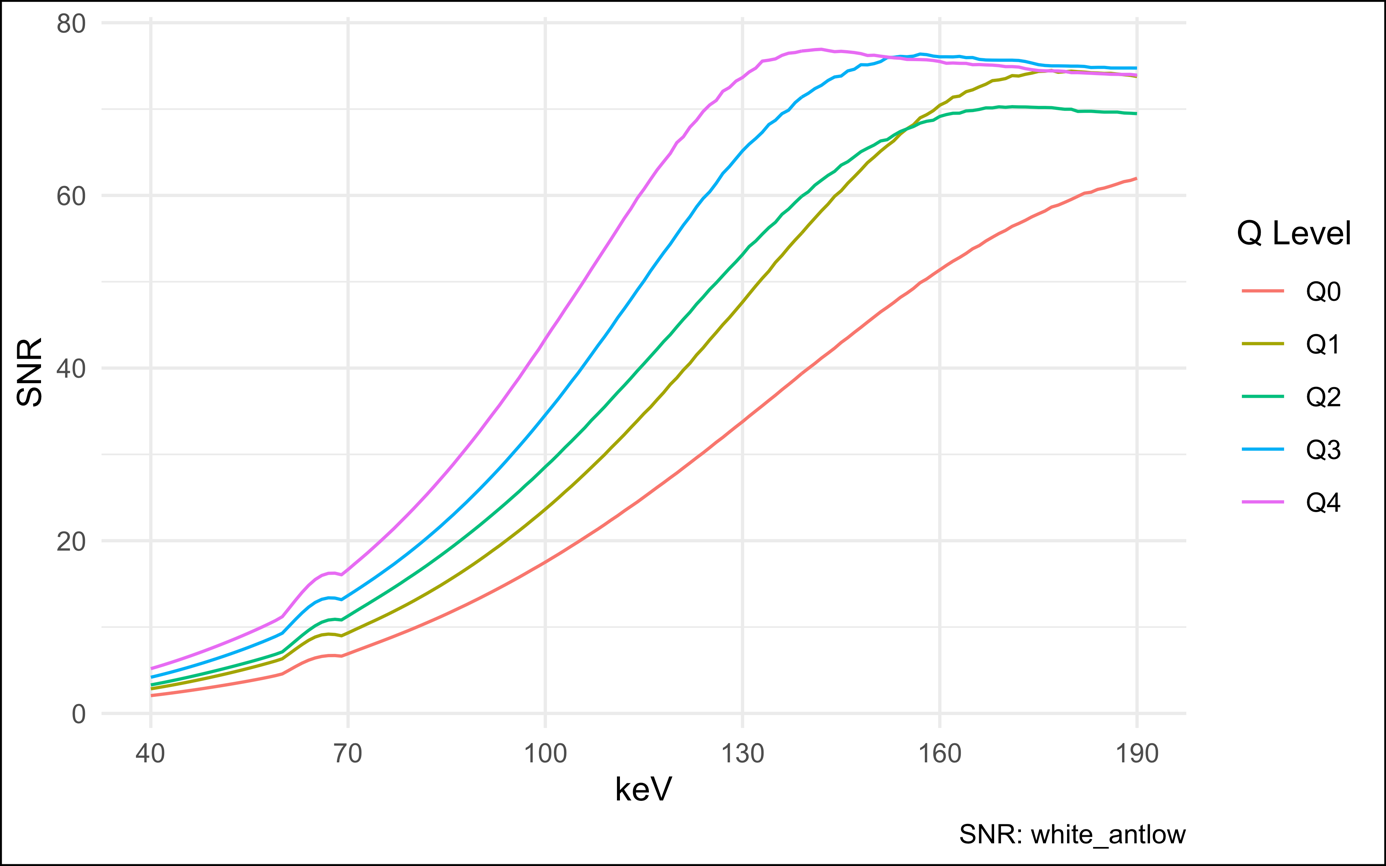


There is a significant difference between the SNR characteristics of the individual virtual monoenergetic reconstructions (Friedman test corrected p < 0.0001) in ROI (13). According to the noise decreasing with each Q level, the SNR increases. The maximum SNR in Q4 is found in the 142 keV VMI with 76.92 ± 38.57. The focally further reduced noise between 60 and 70 keV results in focally increased SNR.

In post hoc testing, a significant difference is found to the keV in the lower range up to 130 keV (130 keV: corrected p = 0.014177) and to the keV in higher range from 160 keV on (160 keV: corrected p = 0.007996).

Selected keV levels with all Q levels and their post hoc tests can be reviewed in the supplemental material (Excel file “PCCT_CCT_Analysis.xlsx”, sheet “analysis_snr”).

## ROI (14): Gray Matter in the Posterior Thalamus


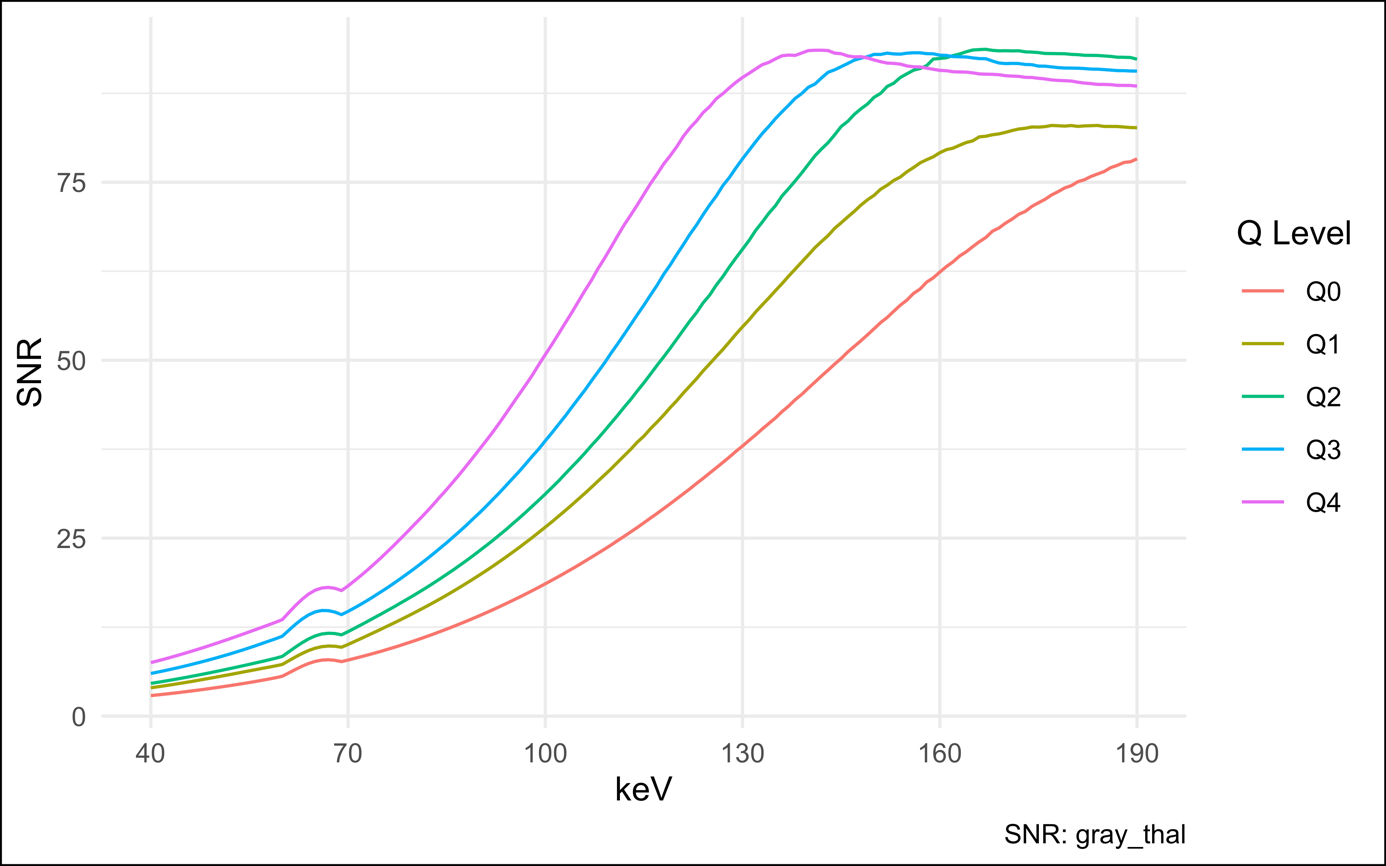


There is a significant difference between the SNR characteristics of the individual virtual monoenergetic reconstructions (Friedman test corrected p < 0.0001) in ROI (14). According to the noise decreasing with each Q level, the SNR increases. The maximum SNR in Q4 is found in the 142 keV VMI with 93.55 ± 30.09. The focally further reduced noise between 60 and 70 keV results in focally increased SNR.

In post hoc testing, a significant difference is found to the keV in the lower range up to 140 keV (140 keV: corrected p = 0.011038) and to the keV in higher range from 143 keV on (143 keV: corrected p = 0.000000).

Selected keV levels with all Q levels and their post hoc tests can be reviewed in the supplemental material (Excel file “PCCT_CCT_Analysis.xlsx”, sheet “analysis_snr”).

## ROI (15): White Matter in the Posterior Internal Capsule


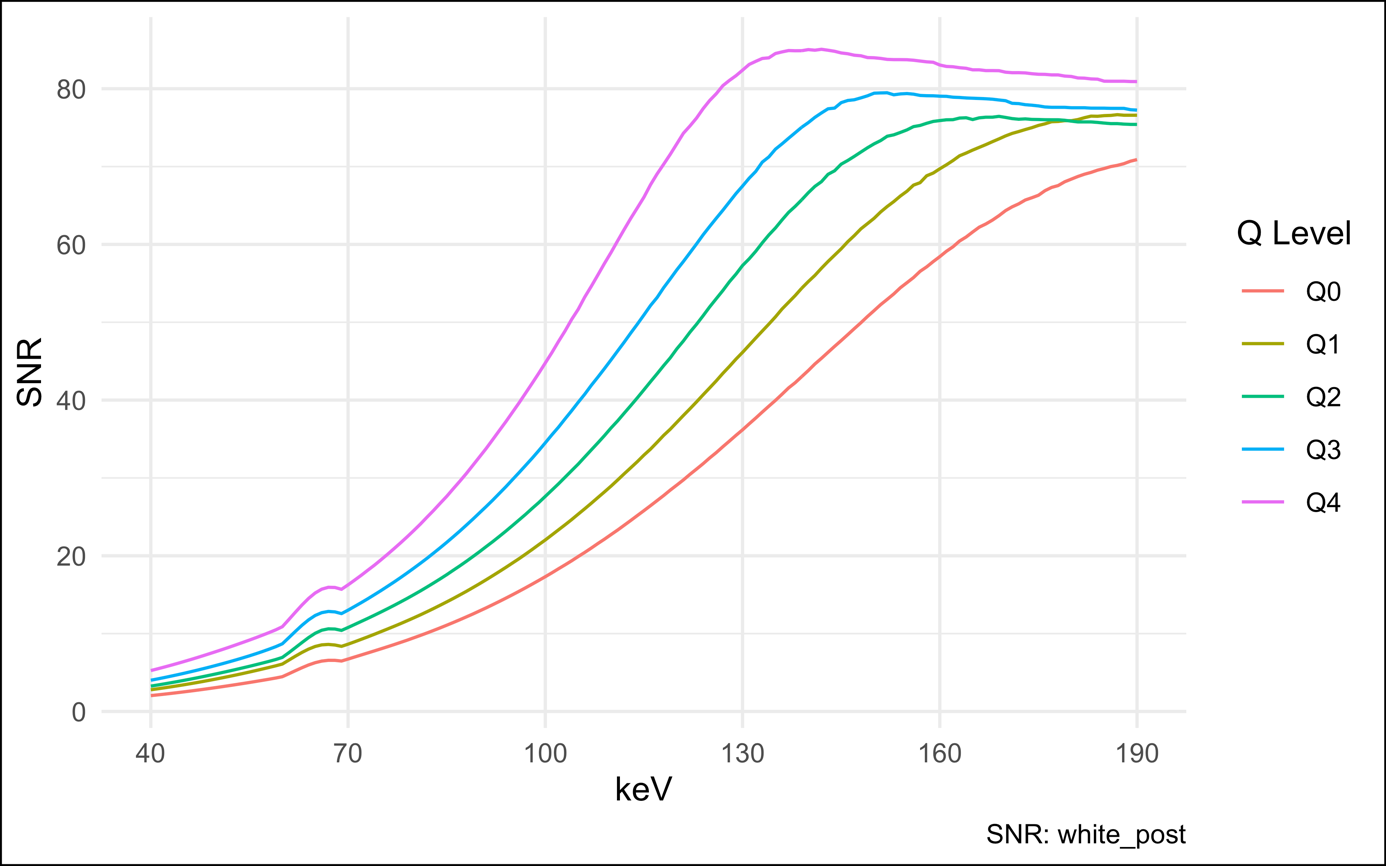


There is a significant difference between the SNR characteristics of the individual virtual monoenergetic reconstructions (Friedman test corrected p < 0.0001) in ROI (15). According to the noise decreasing with each Q level, the SNR increases. The maximum SNR in Q4 is found in the 142 keV VMI with 85.07 ± 32.49. The focally further reduced noise between 60 and 70 keV results in focally increased SNR.

In post hoc testing, a significant difference is found to the keV in the lower range up to 120 keV (120 keV: corrected p = 0.000003) and to the keV in higher range from 146 keV on (146 keV: corrected p = 0.020457).

Selected keV levels with all Q levels and their post hoc tests can be reviewed in the supplemental material (Excel file “PCCT_CCT_Analysis.xlsx”, sheet “analysis_snr”).

## ROI (16): White Matter in the Pons between the Petrous Bones


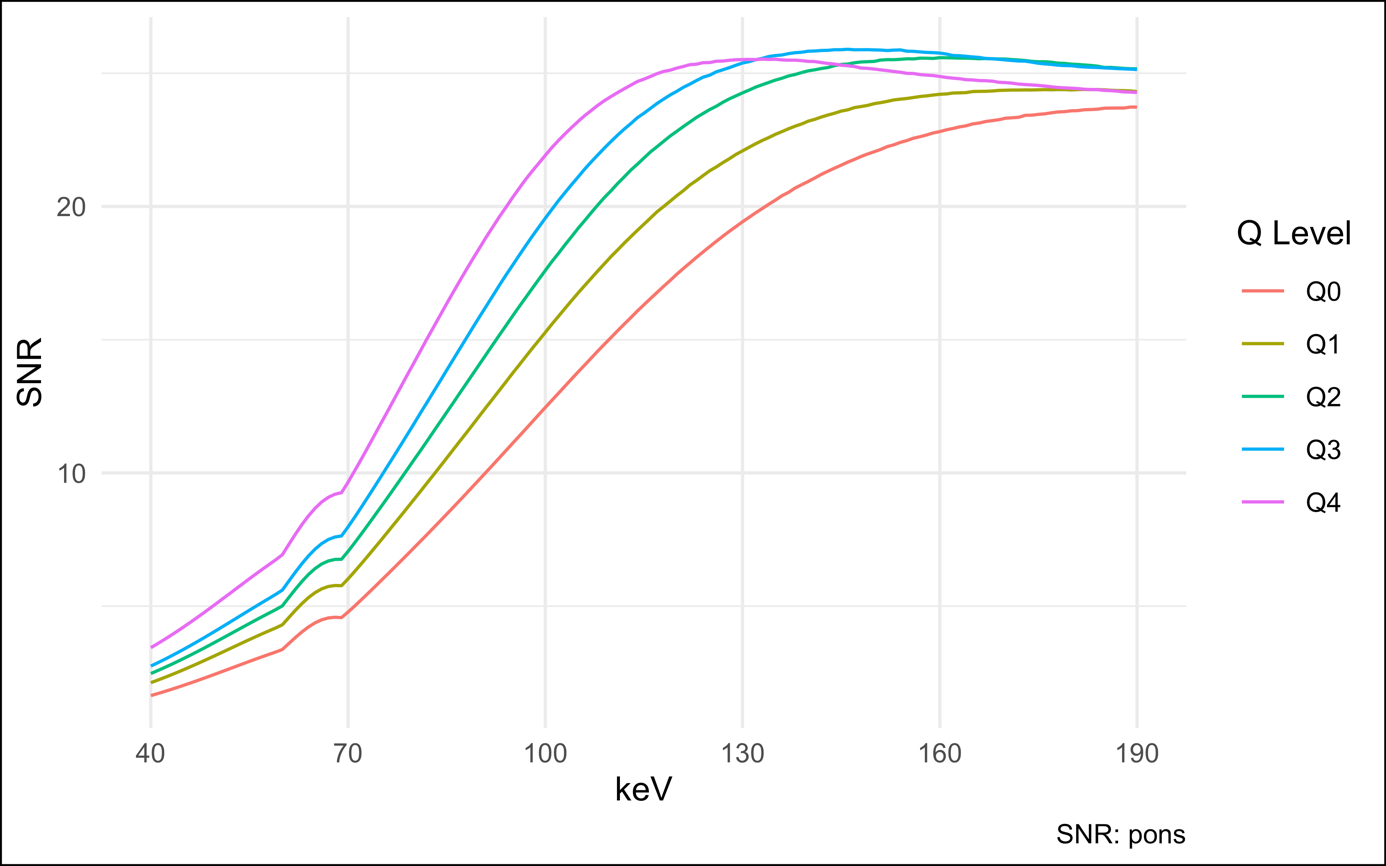


There is a significant difference between the SNR characteristics of the individual virtual monoenergetic reconstructions (Friedman test corrected p < 0.0001) in ROI (16). According to the noise decreasing with each Q level, the SNR increases. The maximum SNR in Q4 is found in the 133 keV VMI with 25.53 ± 8.36. The focally further reduced noise between 60 and 70 keV results in focally increased SNR.

In post hoc testing, a significant difference is found to the keV in the lower range up to 110 keV (80 keV: corrected p = 0.00204) and to the keV in higher range from 160 keV on (160 keV: corrected p = 0.0272).

Selected keV levels with all Q levels and their post hoc tests can be reviewed in the supplemental material (Excel file “PCCT_CCT_Analysis.xlsx”, sheet “analysis_snr”).

# Results: Contrast to Noise Ratio (CNR)

## CNR Directly below the Calvaria: Gray Matter ROI (1) and White Matter ROI (3)


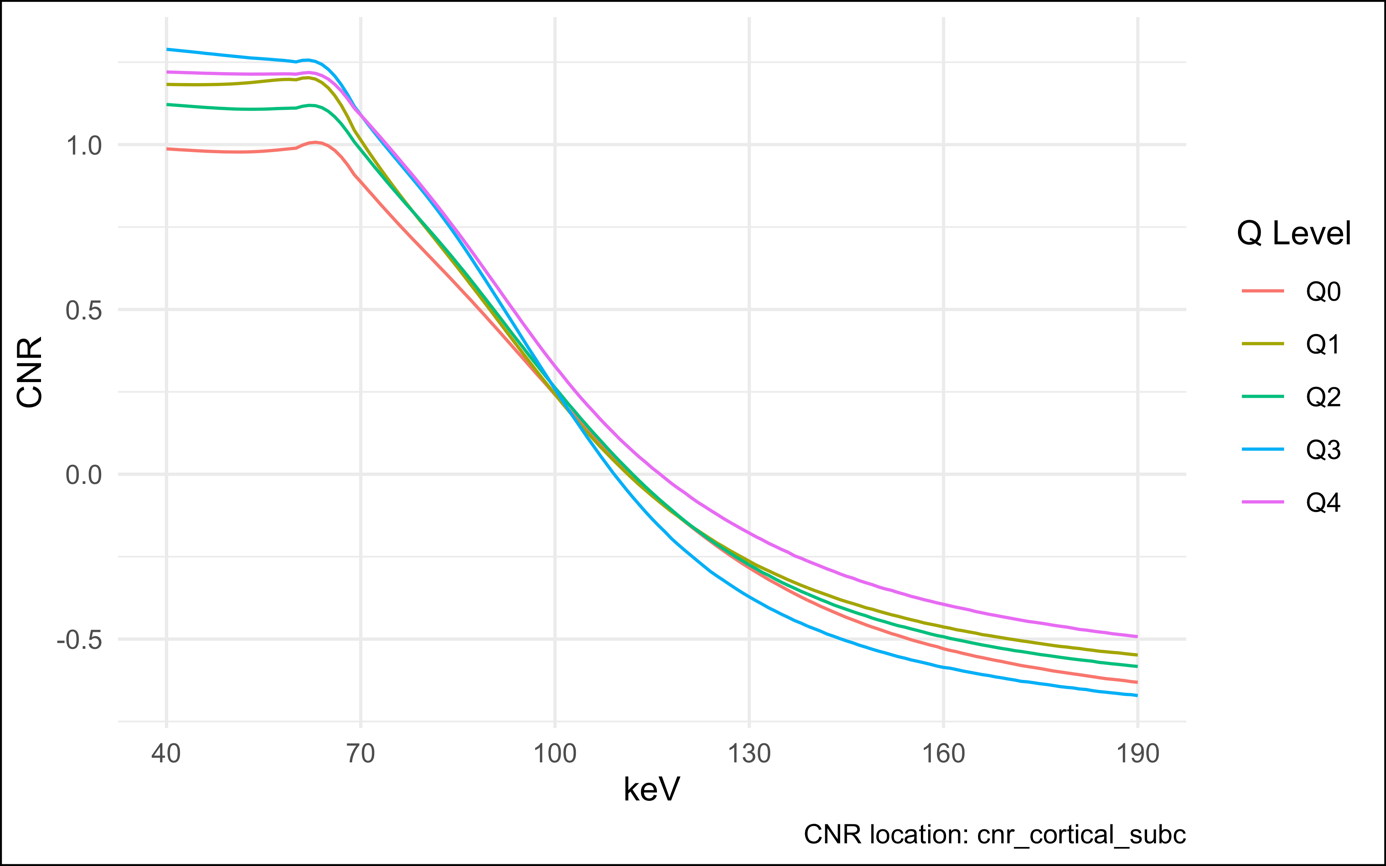


There is a significant difference between the CNR characteristics of the individual virtual monoenergetic reconstructions (Friedman test corrected p < 0.0001) in the gray and white matter directly below the calvaria. Interestingly, the highest CNR in the low keV is achieved with the third level of iterative reconstruction (Q3). Only from the middle keV onwards Q4 produces the highest values. The maximum CNR in Q3 is found in the 40 keV VMI with 1.29 ± 0.98. The focally further reduced noise between 60 and 70 keV also results in focally increased CNR.

In post hoc testing, a significant difference is found to the keV in the higher range from 75 keV on (75 keV: corrected p = 0.006140).

Selected keV levels with all Q levels and their post hoc tests can be reviewed in the supplemental material (Excel file “PCCT_CCT_Analysis.xlsx”, sheet “analysis_cnr”).

## CNR 5 mm below the Calvaria: Gray Matter ROI (2) and White Matter ROI (3)


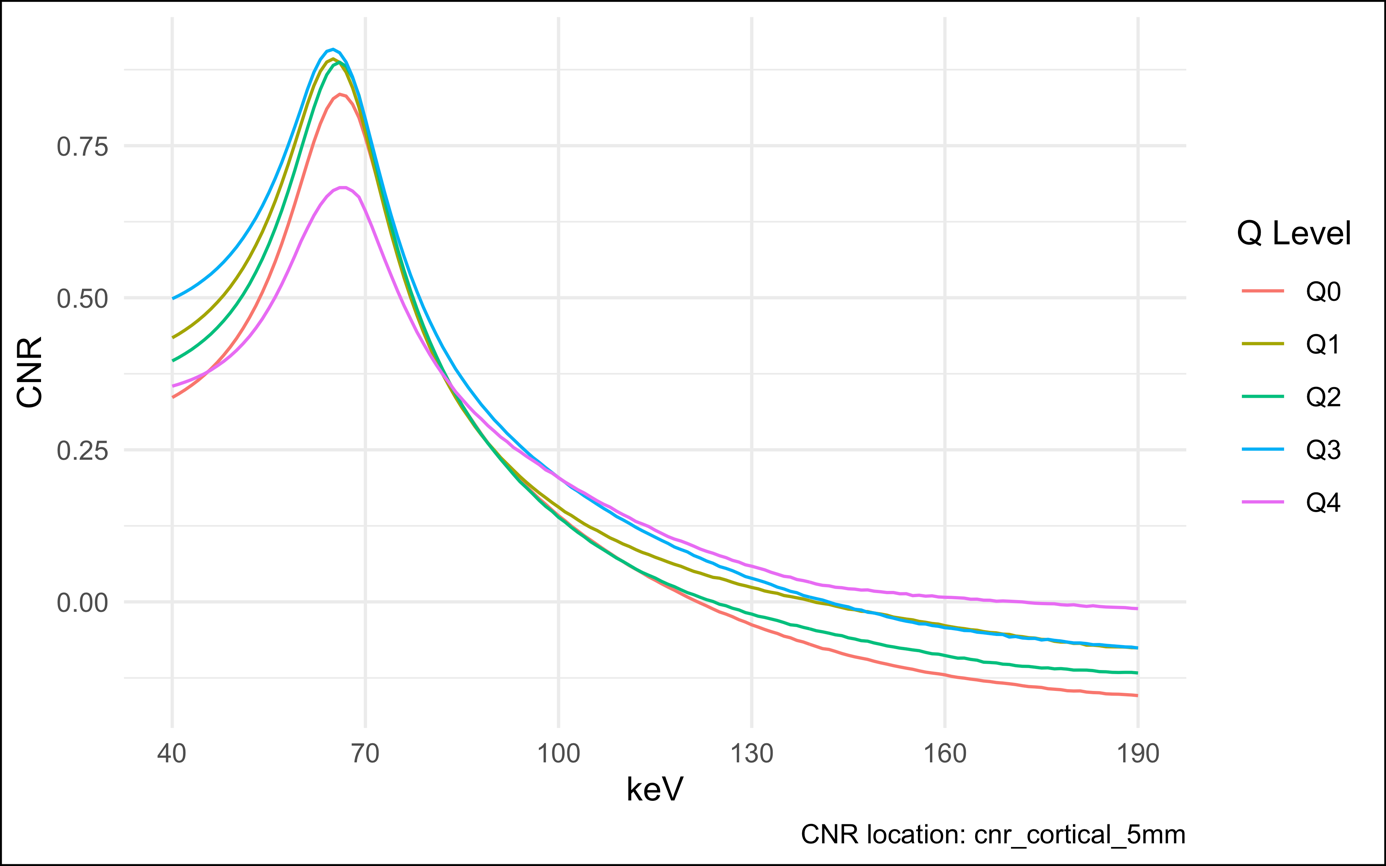


There is a significant difference between the CNR characteristics of the individual virtual monoenergetic reconstructions (Friedman test corrected p < 0.0001) in the gray and white matter 5 mm below the calvaria. Interestingly – like directly below the calvaria – the highest CNR in the low keV is achieved with the third level of iterative reconstruction (Q3). Only from the middle keV onwards Q4 produces the highest values. The maximum CNR in Q3 is found in the 65 keV VMI with 0.91 ± 0.68.

In post hoc testing, a significant difference is found to the keV in the lower range up to 60 keV on (60 keV: corrected p = 0.024880) and to the keV in the higher range from 70 keV on (70 keV: corrected p = 0.047650).

Selected keV levels with all Q levels and their post hoc tests can be reviewed in the supplemental material (Excel file “PCCT_CCT_Analysis.xlsx”, sheet “analysis_cnr”).

## CNR 10 mm below the Calvaria: Gray Matter ROI (4) and White Matter ROI (5)


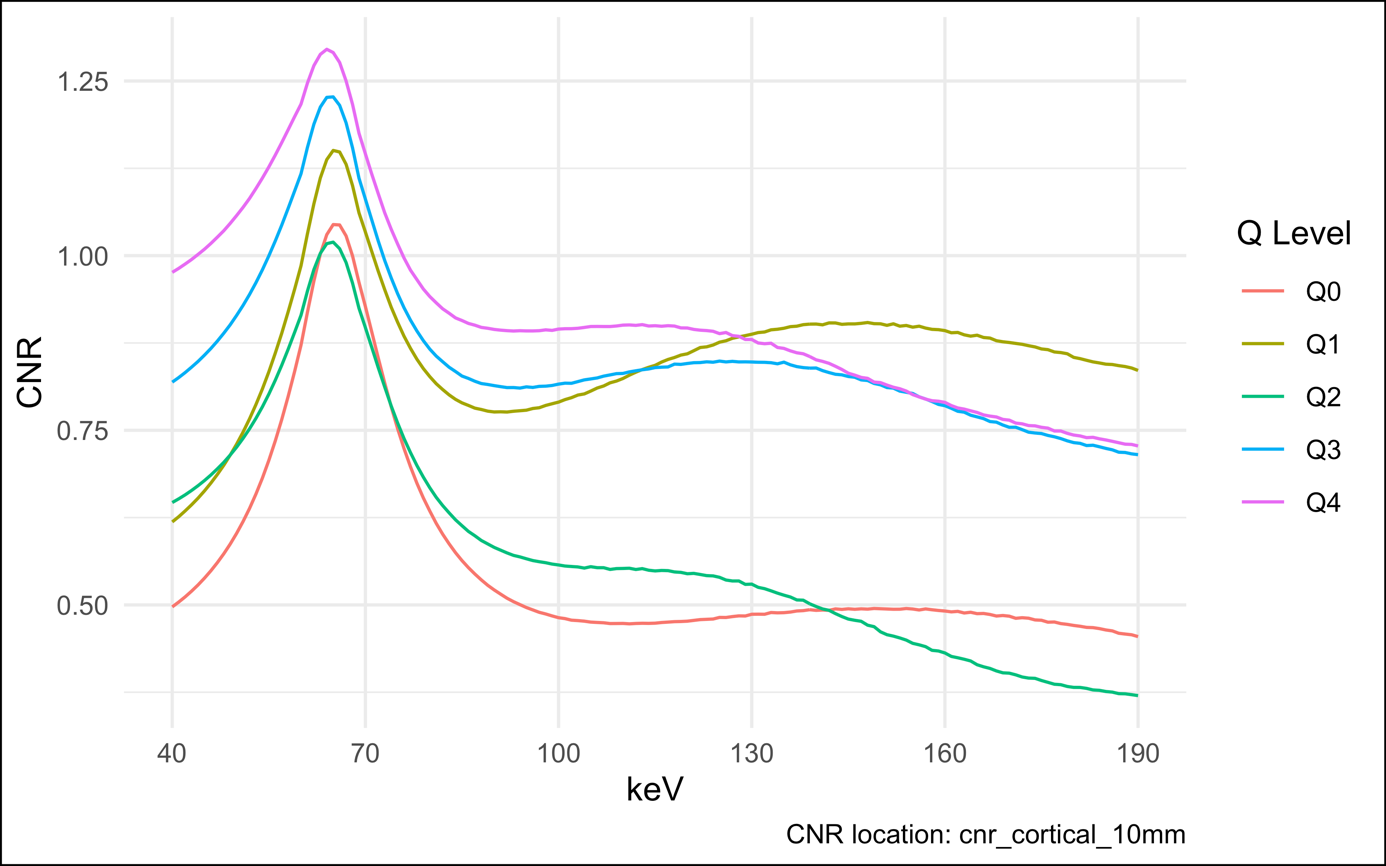


There is a significant difference between the CNR characteristics of the individual virtual monoenergetic reconstructions (Friedman test corrected p < 0.0001) in the gray and white matter 10 mm below the calvaria. The maximum CNR in Q4 is found in the 64 keV VMI with 1.30 ± 0.71.

In post hoc testing, a significant difference is found to the keV in the lower range up to 59 keV on (59 keV: corrected p = 0.042213) and to the keV in the higher range from 68 keV on (68 keV: corrected p = 0.035794).

Selected keV levels with all Q levels and their post hoc tests can be reviewed in the supplemental material (Excel file “PCCT_CCT_Analysis.xlsx”, sheet “analysis_cnr”).

## CNR 15 mm below the Calvaria: Gray Matter ROI (6) and White Matter ROI (7)


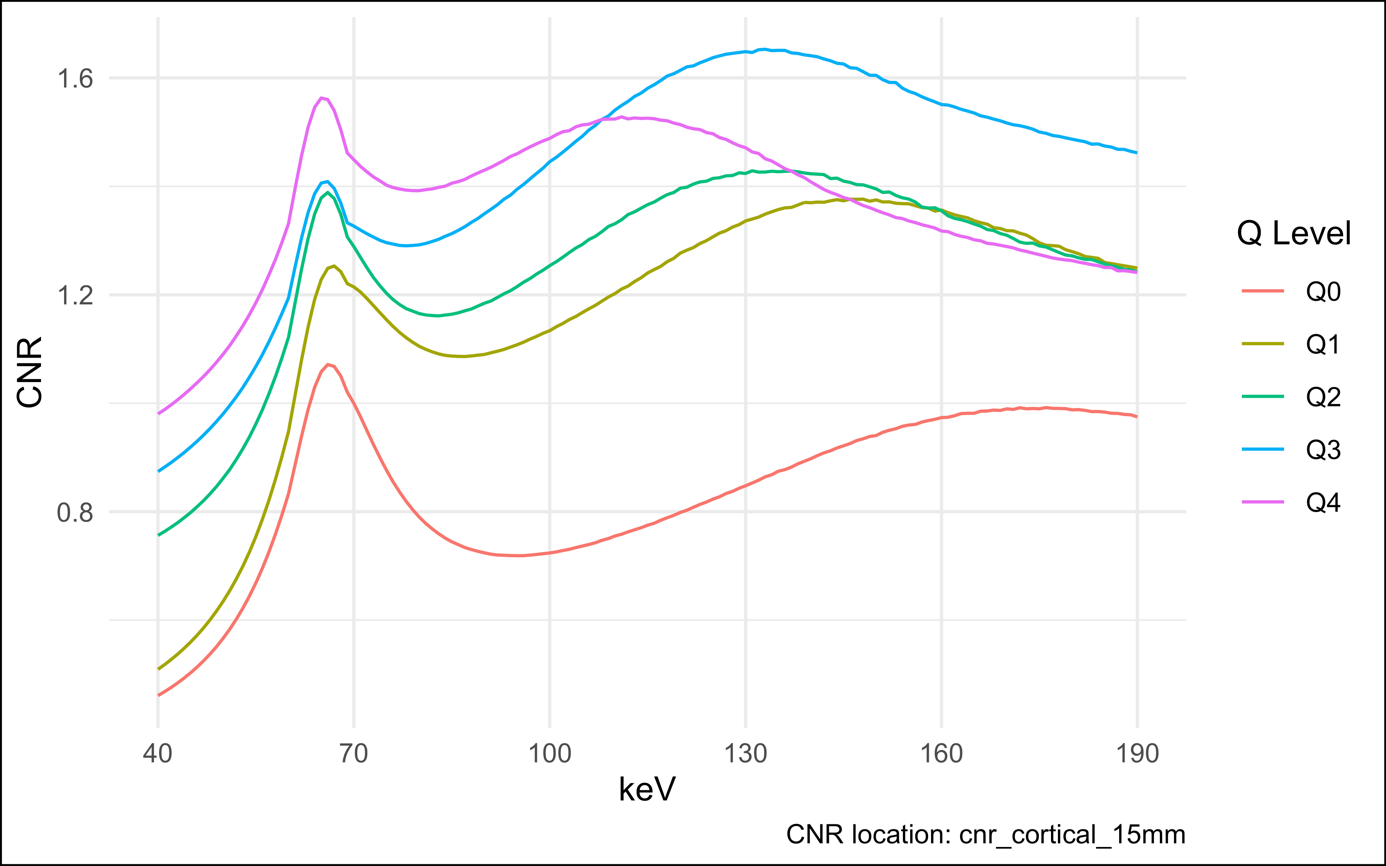


There is a significant difference between the CNR characteristics of the individual virtual monoenergetic reconstructions (Friedman test corrected p < 0.0001) in the gray and white matter 15 mm below the calvaria. As already indicated at 10 mm below the calvaria, one now recognizes a two-peaked distribution with a maximum between 60 and 70 keV, the second maximum follows from 100 keV and is located somewhat differently depending on the Q level. The maximum CNR is found in the Q3 133 keV VMI with 1.65 ± 1.59, a big standard deviation. The dispersion is much smaller at the first maximum between 60 and 70 keV (see the following table, the maximum in the range is highlighted bold).

| CNR location | keV | QIR | mean | sd |
| --- | --- | --- | --- | --- |
| cnr_cortical_15mm | 62 | 4 | 1.457027496 | 0.94318466 |
| cnr_cortical_15mm | 63 | 4 | 1.509213278 | 0.978728375 |
| cnr_cortical_15mm | 64 | 4 | 1.54575967 | 0.997235586 |
| cnr_cortical_15mm | **65** | **4** | **1.562880233** | 0.994543391 |
| cnr_cortical_15mm | 66 | 4 | 1.56001765 | 0.967904139 |
| cnr_cortical_15mm | 67 | 4 | 1.539640482 | 0.92277592 |
| cnr_cortical_15mm | 68 | 4 | 1.504356079 | 0.867178638 |

In post hoc testing, there is no significant difference between the overall maximum in Q3 133 keV and the focal maximum between 60 and 70 keV.

Selected keV levels with all Q levels and their post hoc tests can be reviewed in the supplemental material (Excel file “PCCT_CCT_Analysis.xlsx”, sheet “analysis_cnr”).

## CNR 20 mm below the Calvaria: Gray Matter ROI (8) and White Matter ROI (9)


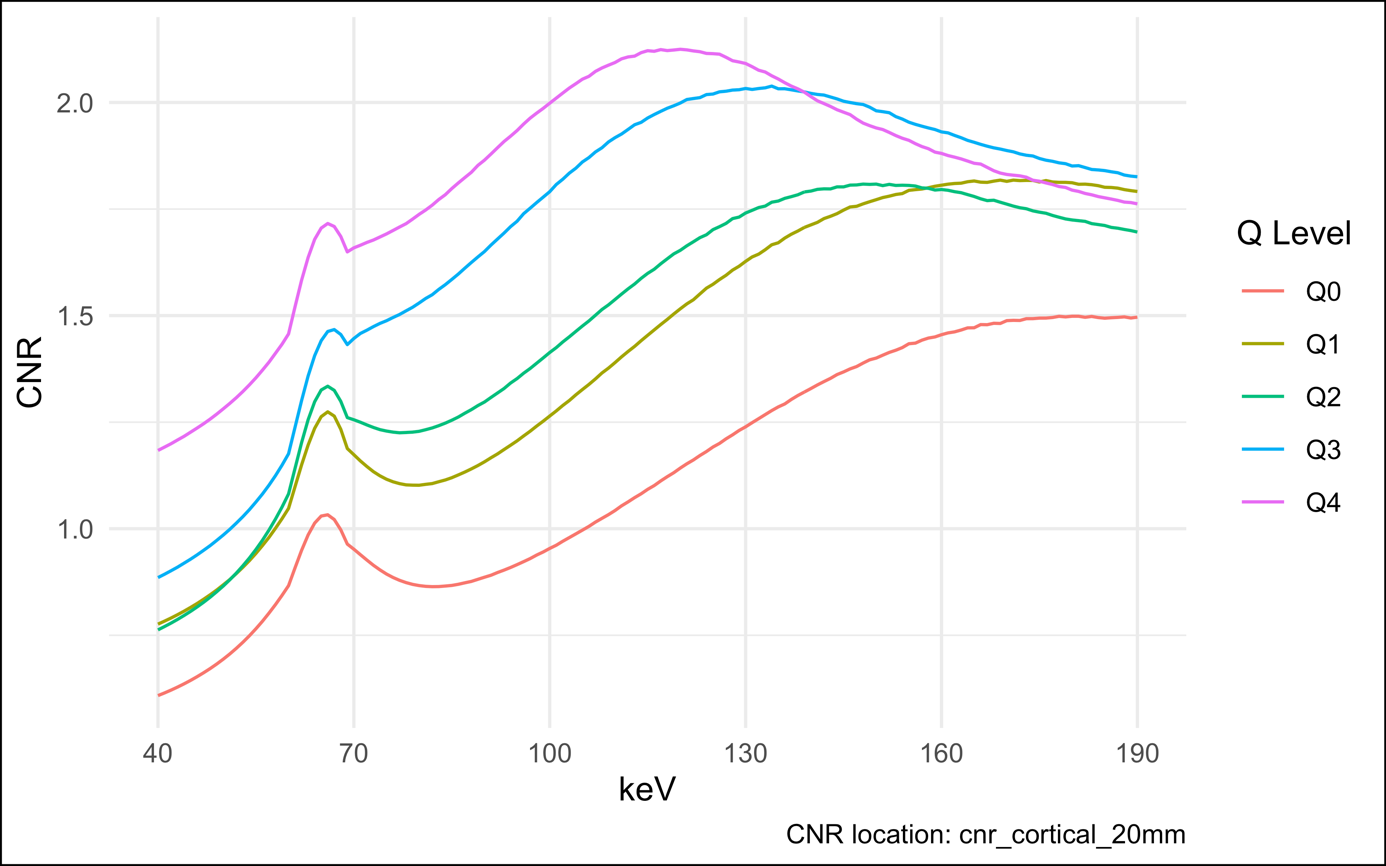


There is a significant difference between the CNR characteristics of the individual virtual monoenergetic reconstructions (Friedman test corrected p < 0.0001) in the gray and white matter 20 mm below the calvaria. Again there is a two-peaked distribution with a maximum between 60 and 70 keV, the second maximum follows from 100 keV and is located somewhat differently depending on the Q level: the lower the level, the further it is shifted to the right. The maximum CNR is found in the Q4 120 keV VMI with 2.12 ± 1.82, again a big standard deviation. The dispersion is smaller at the first maximum between 60 and 70 keV (see the following table, the maximum in the range is highlighted bold).

| CNR location | keV | QIR | mean | sd |
| --- | --- | --- | --- | --- |
| cnr_cortical_20mm | 63 | 4 | 1.636493901 | 1.088163688 |
| cnr_cortical_20mm | 64 | 4 | 1.678924421 | 1.099666258 |
| cnr_cortical_20mm | 65 | 4 | 1.705321345 | 1.102007431 |
| cnr_cortical_20mm | **66** | **4** | **1.716099495** | 1.095830981 |
| cnr_cortical_20mm | 67 | 4 | 1.709078762 | 1.080934425 |
| cnr_cortical_20mm | 68 | 4 | 1.685637365 | 1.058725987 |
| cnr_cortical_20mm | 69 | 4 | 1.649473994 | 1.030376205 |

In post hoc testing, there is no significant difference between the overall maximum in Q4 120 keV and the focal maximum between at 66 keV.

Selected keV levels with all Q levels and their post hoc tests can be reviewed in the supplemental material (Excel file “PCCT_CCT_Analysis.xlsx”, sheet “analysis_cnr”).

## CNR at the Superior Caudate Head: Gray Matter ROI (10) and White Matter ROI (11)


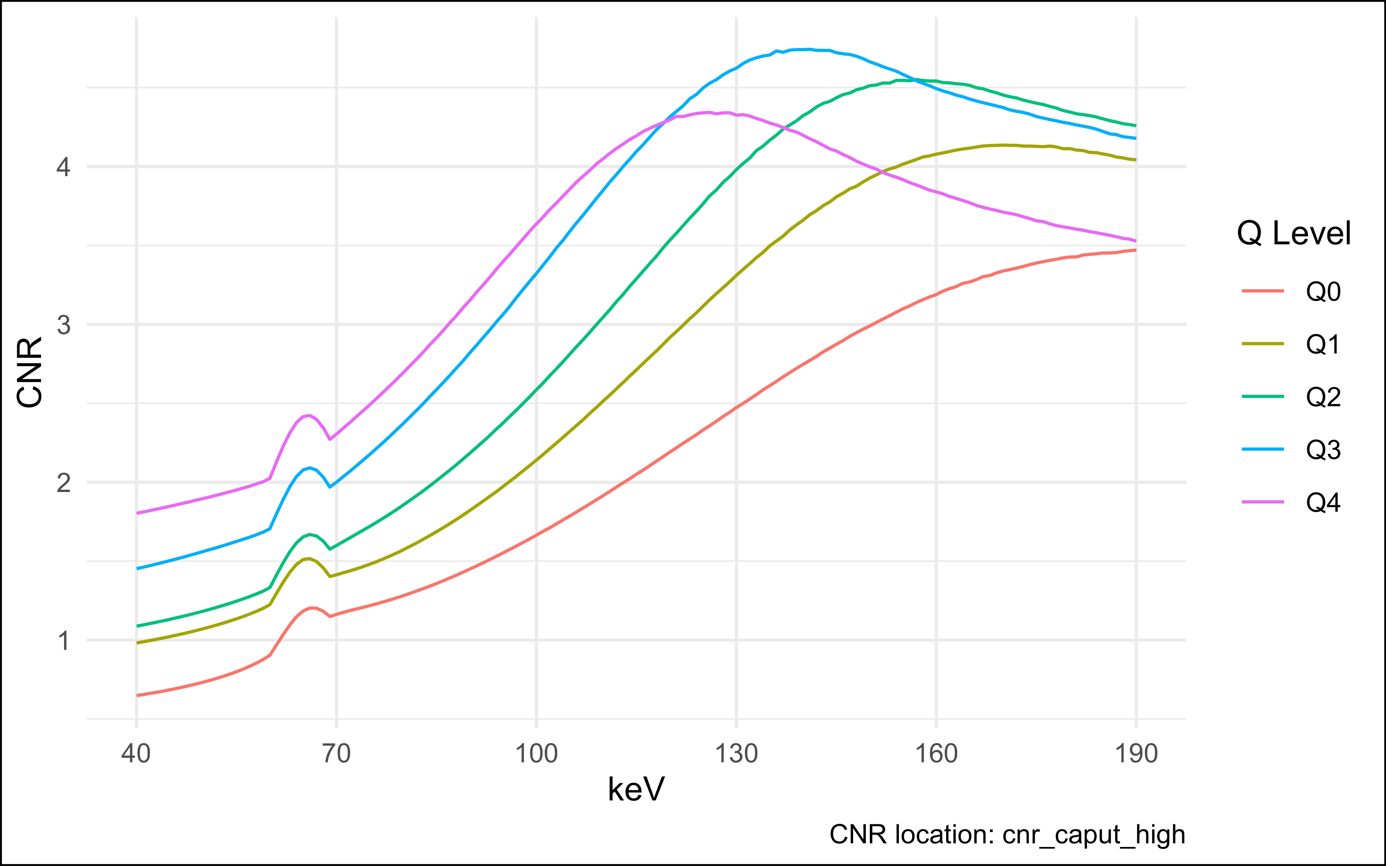


There is a significant difference between the CNR characteristics of the individual virtual monoenergetic reconstructions (Friedman test corrected p < 0.0001) in the gray and white matter at the superior caudate head. Again there is a two-peaked distribution with a maximum between 60 and 70 keV, the second maximum follows from 100 keV and is located somewhat differently depending on the Q level: the lower the level, the further it is shifted to the right. The maximum CNR is found in the Q3 141 keV VMI with 4.74 ± 0.59, here a relatively small dispersion. The first maximum between 60 and 70 keV is found in Q4 (see the following table, the maximum in the range is highlighted bold).

| CNR location | keV | QIR | mean | sd |
| --- | --- | --- | --- | --- |
| cnr_caput_high | 63 | 4 | 2.312998045 | 0.652726175 |
| cnr_caput_high | 64 | 4 | 2.37827193 | 0.675253374 |
| cnr_caput_high | 65 | 4 | 2.415848563 | 0.695302773 |
| cnr_caput_high | **66** | **4** | **2.422879575** | 0.710171611 |
| cnr_caput_high | 67 | 4 | 2.398600819 | 0.723834677 |
| cnr_caput_high | 68 | 4 | 2.345971376 | 0.733425938 |
| cnr_caput_high | 69 | 4 | 2.271747383 | 0.738980437 |

Selected keV levels with all Q levels and their post hoc tests can be reviewed in the supplemental material (Excel file “PCCT_CCT_Analysis.xlsx”, sheet “analysis_cnr”).

## CNR at the Inferior Caudate Head: Gray Matter ROI (10) and White Matter ROI (11)


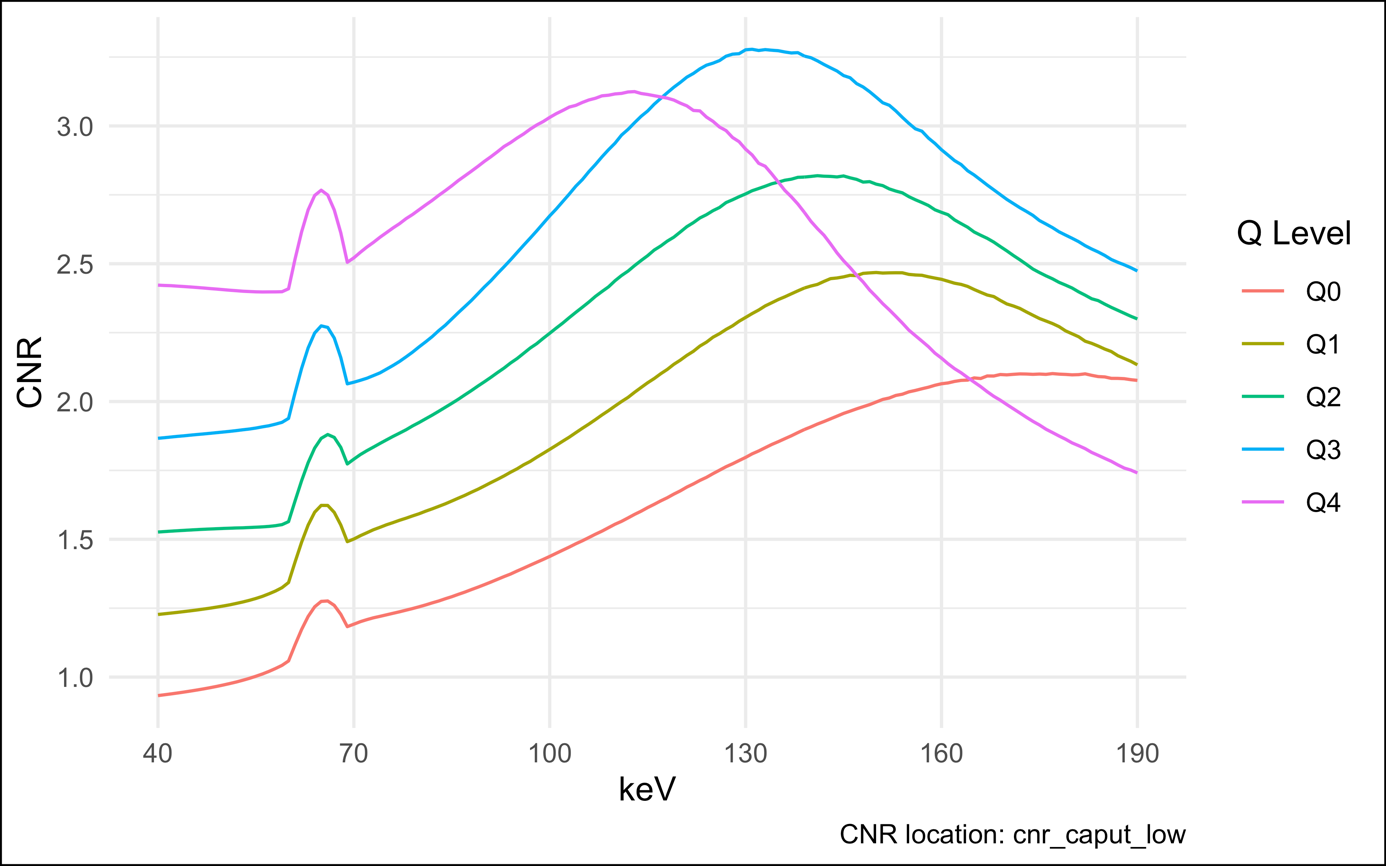


There is a significant difference between the CNR characteristics of the individual virtual monoenergetic reconstructions (Friedman test corrected p < 0.0001) in the gray and white matter at the inferior caudate head. Again there is a two-peaked distribution with a maximum between 60 and 70 keV, the second maximum follows from 100 keV and is located somewhat differently depending on the Q level: the lower the level, the further it is shifted to the right. The maximum CNR is found in the Q3 131 keV VMI with 3.28 ± 2.23. The first maximum between 60 and 70 keV is found in Q4 (see the following table, the maximum in the range is highlighted bold).

| CNR location | keV | QIR | mean | sd |
| --- | --- | --- | --- | --- |
| cnr_caput_low | 62 | 4 | 2.615644767 | 0.677591535 |
| cnr_caput_low | 63 | 4 | 2.695408215 | 0.688116256 |
| cnr_caput_low | 64 | 4 | 2.747703152 | 0.693838444 |
| cnr_caput_low | **65** | **4** | **2.766821105** | 0.693462368 |
| cnr_caput_low | 66 | 4 | 2.749269949 | 0.690787462 |
| cnr_caput_low | 67 | 4 | 2.695085693 | 0.682991512 |
| cnr_caput_low | 68 | 4 | 2.612134685 | 0.674719501 |

Selected keV levels with all Q levels and their post hoc tests can be reviewed in the supplemental material (Excel file “PCCT_CCT_Analysis.xlsx”, sheet “analysis_cnr”).

## CNR at the Thalamus: Gray matter ROI (14) and White Matter ROI (15)


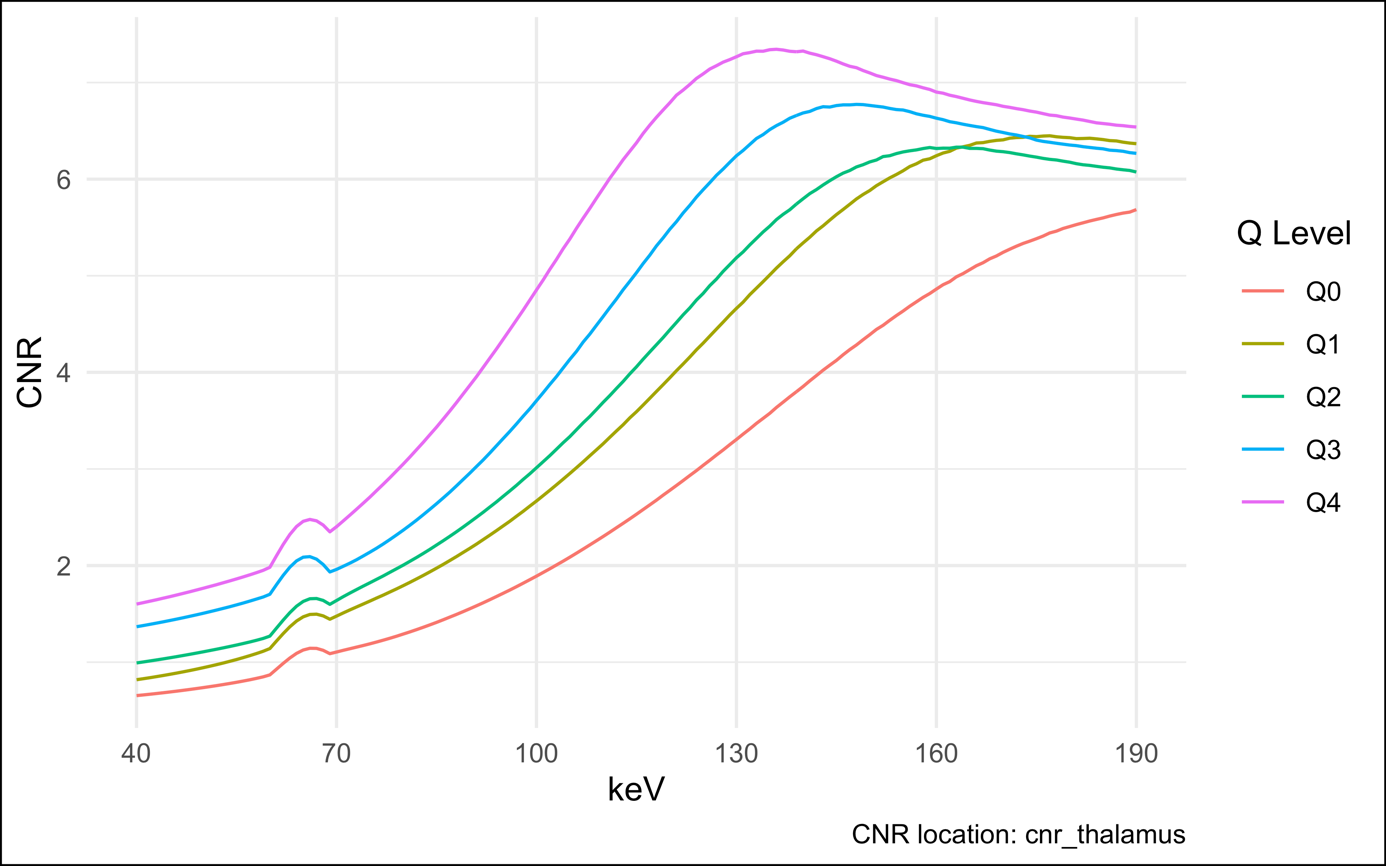


There is a significant difference between the CNR characteristics of the individual virtual monoenergetic reconstructions (Friedman test corrected p < 0.0001) in the gray and white matter at the thalamus. Again there is a two-peaked distribution with a maximum between 60 and 70 keV, the second maximum follows from 130 keV and is located somewhat differently depending on the Q level: the lower the level, the further it is shifted to the right. The maximum CNR is found in the Q4 136 keV VMI with 7.34 ± 2.79. The first maximum between 60 and 70 keV is again found in Q4 (see the following table, the maximum in the range is highlighted bold).

| CNR location | keV | QIR | mean | sd |
| --- | --- | --- | --- | --- |
| cnr_thalamus | 63 | 4 | 2.32206605 | 0.5980178 |
| cnr_thalamus | 64 | 4 | 2.404190882 | 0.616255145 |
| cnr_thalamus | 65 | 4 | 2.4579793 | 0.631616747 |
| cnr_thalamus | **66** | **4** | **2.47748471** | 0.644045304 |
| cnr_thalamus | 67 | 4 | 2.462978886 | 0.652295974 |
| cnr_thalamus | 68 | 4 | 2.419222706 | 0.656071011 |
| cnr_thalamus | 69 | 4 | 2.349098319 | 0.651940993 |

Selected keV levels with all Q levels and their post hoc tests can be reviewed in the supplemental material (Excel file “PCCT_CCT_Analysis.xlsx”, sheet “analysis_cnr”).

In post hoc testing, a significant difference is found between the overall maximum at 136 keV an to the keV in the lower range up to 120 keV on (120 keV: corrected p = 0.000032) and to the keV in the higher range from 150 keV on (150 keV: corrected p = 0.000000).

# Results: Comparative graphs of the CNR

| 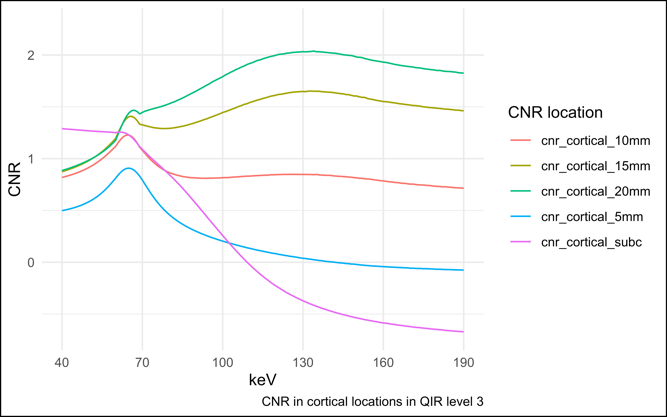 | 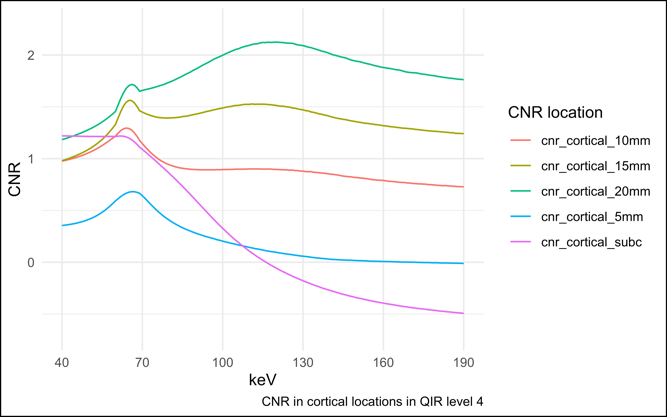 |
| --- | --- |

These graphs again represent the curves of CNR in the different locations. On the left side there is always Q3, on the right Q4. In the last figure all CNR locations are summarized again in the same scale.

| 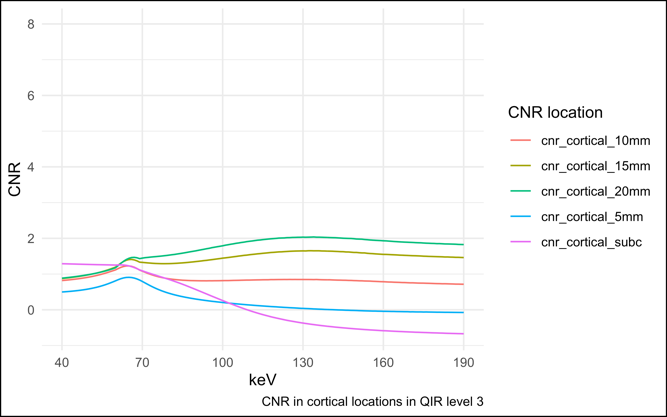 | 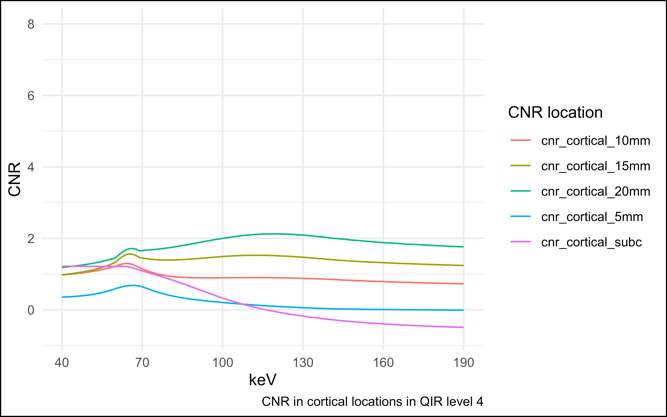 |
| --- | --- |
| 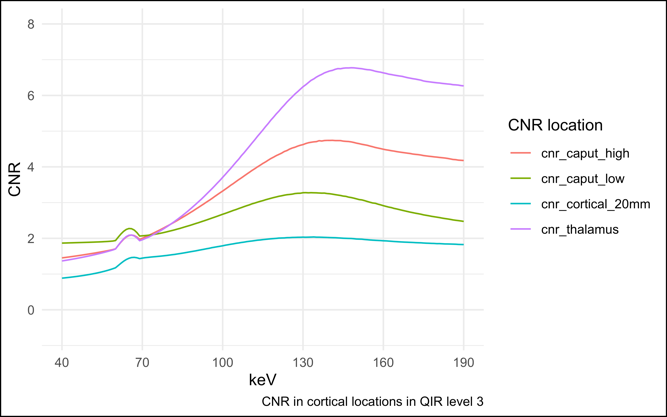 | 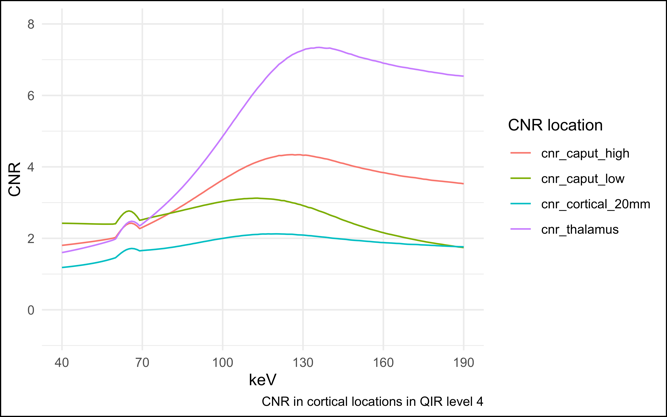 |

# Literature

Michael, A. E., Boriesosdick, J., Schoenbeck, D., Lopez-Schmidt, I., Kroeger, J. R., Moenninghoff, C., . . . Niehoff, J. H. (2022). Photon Counting CT Angiography of the Head and Neck: Image Quality Assessment of Polyenergetic and Virtual Monoenergetic Reconstructions. *Diagnostics (Basel), 12*(6). doi:10.3390/diagnostics12061306

Michael, A. E., Boriesosdick, J., Schoenbeck, D., Woeltjen, M. M., Saeed, S., Kroeger, J. R., . . . Niehoff, J. H. (2022). Image-Quality Assessment of Polyenergetic and Virtual Monoenergetic Reconstructions of Unenhanced CT Scans of the Head: Initial Experiences with the First Photon-Counting CT Approved for Clinical Use. *Diagnostics, 12*(2), 265. Retrieved from <https://www.mdpi.com/2075-4418/12/2/265>

Neuhaus, V., Abdullayev, N., Große Hokamp, N., Pahn, G., Kabbasch, C., Mpotsaris, A., . . . Borggrefe, J. (2017). Improvement of Image Quality in Unenhanced Dual-Layer CT of the Head Using Virtual Monoenergetic Images Compared With Polyenergetic Single-Energy CT. *Invest Radiol, 52*(8), 470-476. doi:10.1097/rli.0000000000000367

Pomerantz, S. R., Kamalian, S., Zhang, D., Gupta, R., Rapalino, O., Sahani, D. V., & Lev, M. H. (2013). Virtual monochromatic reconstruction of dual-energy unenhanced head CT at 65-75 keV maximizes image quality compared with conventional polychromatic CT. *Radiology (Easton PA), 266*(1), 318-325. doi:10.1148/radiol.12111604

R Core Team. (2021). R: A Language and Environment for Statistical Computing. Retrieved from <https://www.R-project.org/>
